# Pathogenic variants in the autophagy-tethering factor EPG5 drive neurodegeneration through mitochondrial dysfunction and innate immune activation

**DOI:** 10.1038/s41467-026-73538-7

**Published:** 2026-05-26

**Authors:** Kritarth Singh, Hormos Salimi Dafsari, Olivia Gillham, Haoyu Chi, Ivet Mandzhukova, Ioanna Kourouzidou, Preethi Sheshadri, Chih-Yao Chung, Valeria Pingitore, Fleur Vansenne, David L. Selwood, Diana Pendin, Gyorgy Szabadkai, Manolis Fanto, Heinz Jungbluth, Michael R. Duchen

**Affiliations:** 1 Department of Cell and Developmental Biology and Consortium for Mitochondrial Research, University College London, Gower Street, London, UK; 2 Department of Pediatrics, Faculty of Medicine and University Hospital Cologne, University of Cologne, Cologne, Germany; 3 Max-Planck-Institute for Biology of Aging and Cologne Excellence Cluster for Ageing-associated Diseases, Cologne, Germany; 4 Department of Paediatric Neurology, Evelina London Children’s Hospital, Guy’s & St Thomas’ NHS Foundation Trust, London, UK; 5 Department of Health and Biomedical Sciences, Universidad Loyola Andalucía, Seville, Spain; 6 Drug Discovery, UCL Wolfson Institute for Biomedical Research, University College London, London, UK; 7 Department of Genetics, University Medical Center Groningen, University of Groningen, Groningen, Netherlands; 8 Neuroscience Institute, National Research Council, Padua, Italy; 9 Department of Biomedical Sciences, University of Padua, Padua, Italy; 10 Department of Basic and Clinical Neuroscience, IoPPN, King’s College London, London, UK; 11 Randall Centre for Cell and Molecular Biophysics, Muscle Signalling Section, Faculty of Life Sciences and Medicine (FoLSM), King’s College London, London, UK

**Keywords:** Calcium signalling, Neurodegeneration, Mechanisms of disease, Mitophagy, Cellular neuroscience

## Abstract

The autophagy-tethering factor ectopic P-granule 5 autophagy protein (EPG5) plays a key role in autophagosome-lysosome fusion. Impaired autophagy associated with pathogenic variants in *EPG5* causes a rare devastating multisystem disorder known as Vici syndrome, which features neurodevelopmental defects, severe progressive neurodegeneration and immunodeficiency. The pathophysiological mechanisms driving disease presentation and progression are only partially understood. In patient-derived fibroblasts and iPS cells differentiated to cortical neurons, we find that impaired mitophagy leads to mitochondrial bioenergetic dysfunction. Physiological cytosolic Ca^2+^ transients result in unexpected mitochondrial Ca^2+^ overload despite a decrease in mitochondrial membrane potential. This is attributed to downregulation of MICU1. Ca^2+^ signals cause mitochondrial depolarisation, mtDNA release and activation of the cGAS-STING pathway, reversed by pharmacological inhibition of the mitochondrial permeability transition pore (mPTP) or of the STING pathway. Thus, we identify a pathophysiological cascade driving disease progression associated with EPG5 deficiency, including impaired mitochondrial bioenergetics, mitochondrial Ca^2+^ overload, vulnerability to mPTP opening and activation of innate immune signalling, signposting multiple potential therapeutic targets.

## Abstract (web summary)

Mutations in the autophagy protein EPG5 cause multisystem disease, including neurodegeneration. This study shows that impaired mitophagy drives mitochondrial Ca^2+^ overload with permeability transition pore opening, leading to mtDNA escape and cGAS–STING activation, highlighting therapeutic targets for Vici syndrome.

## Introduction

Autophagy is a fundamental, evolutionarily conserved intracellular homoeostatic process with essential roles in metabolic adaptation, defence against infections and the quality control of defective proteins and organelles including mitochondria^1^. Ectopic P-granules 5 autophagy tethering factor (EPG5), initially identified in *Caenorhabditis elegans*, functions as a tethering protein in concert with specific SNARE complexes to facilitate specific fusion events between autophagosomes and lysosomes and the formation of degradative autolysosomes^2^. Recessive pathogenic variants in the human *EPG5* gene cause a spectrum of neurodevelopmental disorders termed as *EPG5*-related disorders (*EPG5*-RD) ranging from the severe early-onset multisystem disorder Vici syndrome to relatively milder neurodevelopmental manifestations with progressive neurodegeneration later in life^3,4^. Vici syndrome patients are characterised by the key diagnostic features of microcephaly, callosal agenesis, cataracts, hypopigmentation, cardiomyopathy, (combined) immunodeficiency and failure to thrive. The presenting neurodevelopmental disorder typically progresses to severe progressive neurodegeneration and multisystem involvement with a median life expectancy of approximately 24 months^5^. While most bi-allelic truncating variants in *EPG5* that cause loss of function are associated with Vici syndrome, bi-allelic missense variants in *EPG5* can lead to less severe clinical presentations. An attenuated EPG5 function is predicted to underlie the phenotypic variability seen in *EPG5*-RD^6,7^. However, it is unclear whether the residual function of pathogenic *EPG5* variants correlates directly to a corresponding defect in autophagy alone or whether other downstream cellular processes are also disrupted. Interestingly, preliminary observations indicate an increased frequency of adult-onset neurodegenerative disorders in (putative) heterozygous *EPG5* variant carriers, suggesting a potential dosage effect^7^.

Previous studies have reported that the histopathological appearance of *EPG5*-related Vici syndrome, often including marked ultrastructural mitochondrial abnormalities and decreased respiratory chain enzyme activity, may mimic primary mitochondrial disorders^3,8^ and some patients have been suspected to have a mitochondrial cytopathy before the causative pathogenic *EPG5* variants were genetically confirmed^9,10^. Our recent work has demonstrated that impaired mitophagy and the resulting mitochondrial dysfunction play a significant role in *EPG5*-related Vici syndrome and that some features of the disorder may be a direct consequence of mitochondrial dysfunction^7^. Impaired mitochondrial energy metabolism has been recognised as a key factor in the pathogenesis of many common adult-onset neurodegenerative disorders including amyotrophic lateral sclerosis (ALS), Parkinson’s and Alzheimer’s disease^11–14^. Mitochondrial dysfunction resulting from impaired mitochondrial quality control can lead to impaired ATP homoeostasis, oxidative stress, impaired Ca^2+^ buffering and altered mitochondrial Ca^2+^ signalling, all of which contribute to neuronal dysfunction and cell death characteristic of many neurodegenerative disorders^15,16^.

Mitochondria actively maintain neuronal Ca^2+^ homoeostasis through Ca^2+^ uptake and efflux pathways during physiological cytosolic [Ca^2+^] ([Ca^2+^]_c_) signals^17,18^. Mitochondrial Ca^2+^ uptake is mediated by a Ca^2+^-selective ion channel, the mitochondrial calcium uniporter (MCU) located in the inner mitochondrial membrane (IMM). This hetero-oligomeric channel is composed of the pore-forming protein, MCU^19,20^, a scaffold, essential MCU regulator (EMRE)^21^, and Ca^2+^-sensitive gatekeepers, MICU1, MICU2, and MICU3^22–24^. The dimer of MICU proteins in concert with EMRE exerts a tight control on mitochondrial Ca^2+^ entry during changes in local [Ca^2+^]_c_^25,26^. A rise in mitochondrial matrix [Ca^2+^] ([Ca^2+^]_m_) increases the rate of TCA cycle-driven NADH generation and the rate of oxidative ATP production^27,28^. Accumulation of Ca^2+^ in mitochondria is balanced by Ca^2+^ efflux through the NLCX exchanger which normally maintains a low [Ca^2+^]_m_^29,30^. Supraphysiological accumulation of Ca^2+^, however, can trigger the opening of a large conductance channel in the IMM, the mitochondrial permeability transition pore (mPTP), resulting in the collapse of ΔΨ_m_, ATP hydrolysis by the F_O_F_1_ ATPase and mitochondrial osmotic swelling^31,32^. This mitochondrial catastrophe seems to be a common final path driving mitochondrial Ca^2+^ overload-induced excitotoxic injury and neuronal cell death as evident in *MICU1*-KO mice^33^ and implicated in neurodegenerative diseases, such as ALS^34^, Alzheimer’s^35^ and Parkinson’s disease^36^ as well as in several muscular dystrophies and myopathies^37^.

Under physiological conditions, mitochondrial damage–associated molecular patterns (DAMPs), including mtDNA and mtRNA, are generally inactivated during intrinsic apoptosis, as widespread mPTP opening and mitochondrial outer membrane permeabilisation (MOMP) coincide with rapid activation of the apoptotic caspase cascade, resulting in immunologically silent cell death^38,39^. In contrast, accumulating evidence indicates that chronic or sublethal mitochondrial stress can promote the release of mtDNA from a limited subset of mitochondria without triggering overt apoptosis^40,41^. Once in the cytosol, mtDNA is recognised as a genotoxic danger signal by the cyclic GMP–AMP synthase (cGAS)–stimulator of interferon genes (STING) pathway, leading to activation of type I/III interferon and NF-κB–dependent inflammatory signalling^42^. Mitochondrial Ca²⁺ overload has emerged as a key upstream driver of this process^43^. Mitochondrial Ca^2+^ overload may also promote the release of mtDNA through limited mPTP opening in a subset of damaged mitochondria while avoiding overt cell death, similar to mechanisms described in cellular senescence^41^. Importantly, mtDNA-driven innate immune activation has been implicated in low-grade, chronic inflammatory responses that contribute to progressive neuronal dysfunction and loss, particularly in the context of impaired mitophagy, a pathological feature shared by several late-onset neurodegenerative disorders^44–46^.

Mitochondrial Ca^2+^ overload culminating in the loss of mitochondrial function, Ca^2+^-dependent cell death, inflammation, and progressive cell loss has been implicated in major neurodegenerative diseases^47^. Dysregulated mitochondrial Ca^2+^ homoeostasis may underlie progressive infantile epileptic encephalopathy found in nearly two-thirds of patients with *EPG5*-RD^48^ while the associated chronic inflammatory response may explain the elevated cytokine levels reported in Vici syndrome patients^49^. However, direct evidence defining the molecular mechanism that link aberrant mitochondrial Ca^2+^ signalling leading to mitochondrial dysfunction and sustained inflammatory activation remains limited and poorly understood.

Our recent work demonstrated that impaired mitophagy flux, resulting from defective autophagosome-lysosome fusion, permits the progressive accumulation of dysfunctional mitochondria and represents a major pathogenic mechanism underlying the neurodegeneration and inflammatory dysregulation seen in patients with Vici syndrome and other *EPG5*-RDs^7^. We now identify impaired mitochondrial Ca^2+^ homoeostasis as the causative mechanism driving mtDNA-driven STING- type I IFN inflammatory response in patient-derived fibroblasts with truncating and missense *EPG5* variants. iPSC-derived cortical neurons carrying a founder *EPG5* pathogenic variant showed increased sensitivity to excitotoxicity in response to low concentrations of glutamate that were innocuous in control cells, with responses characterised by mitochondrial Ca^2+^ overload, delayed calcium deregulation (DCD), loss of ΔΨ_m_ and neuronal cell death. We demonstrate that impaired mitochondrial bioenergetic function and Ca^2+^ overload found in patient-derived cells and *EPG5* mutant neurons were attributable to the downregulation of MICU1 and increased susceptibility to mPTP opening. Remarkably, pharmacological inhibition of mPTP opening attenuated inflammatory signals and rescued mitochondrial bioenergetic function in patient-derived cells and *EPG5* mutant neurons. These findings highlight a key pathophysiological mechanism in Vici syndrome and other *EPG5*-RD and signpost potential therapeutic strategies for the rapidly expanding phenotypic and genetic spectrum of patients with *EPG5*- and other autophagy-related disorders.

## Results

### Mitochondrial bioenergetic function is impaired in patient-derived fibroblasts bearing pathogenic *EPG5* variants

**Fig. 1:**
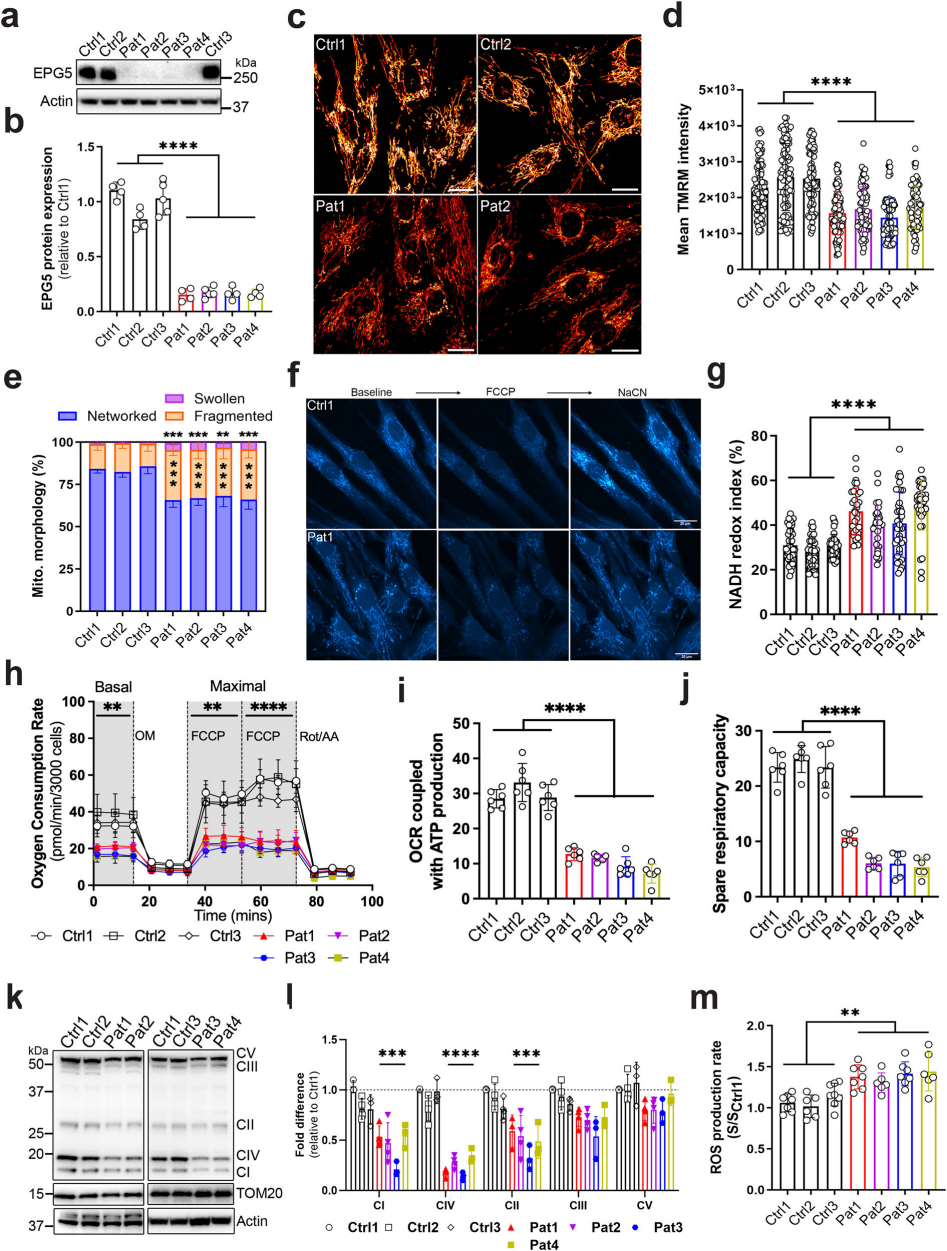
Patient-derived fibroblasts with pathogenic *EPG5* variants show impaired mitochondrial bioenergetic function and respiratory defect. **a** Immunoblot of EPG5 in controls and patient-derived fibroblasts. Actin was used as a loading control. **b** EPG5 abundance normalised to Ctrl1. (*n* = 4, ****p = 1.67 × 10⁻⁸). **c** Representative TMRM images of ΔΨ_m_ (mitochondrial membrane potential) in Ctrl1, Ctrl2, Pat1 and Pat2 fibroblasts. Scale bars, 20 μm. **d** Quantification of steady-state ΔΨ_m_, (*n* = 90, 95, 85, 88, 87, 95 and 84 cells for Ctrl1, Ctrl2, Ctrl3, Pat1, Pat2, Pat3 and Pat4 respectively, ****p = 2.33 × 10^-5^). **e** Mitochondrial morphology (networked, fragmented and swollen) represented as percentage of total mitochondria, (*n* = 3, **p = 0.0034, ***p = 0.0002, 0.0008, 0.0002, 0.0007, 0.0002, 0.0008, 0.0002). **f** Representative NADH autofluorescence images of Ctrl1 and Pat1 fibroblasts at baseline and after FCCP and NaCN, Scale bars: 20 μm. **g** NADH redox index plotted as percentage of the minimum (FCCP) and maximum (NaCN) signal, (*n* = 40, 34, 35, 33, 34, 38 and 39 cells for Ctrl1, Ctrl2, Ctrl3, Pat1, Pat2, Pat3 and Pat4 respectively, ****p = 3.24 × 10⁻^7^). **h** Normalised OCR traces of control and patient fibroblasts, (*n* = 6 runs, **p = 0.0043 and 0.0038, ****p = 1.08 × 10⁻⁸). **i**,**j** Normalised ATP-linked respiration and spare respiratory capacity of controls and patient fibroblasts, (*n* = 6 wells, ****p = 1.73 × 10⁻⁸, 8.66 × 10⁻^7^). **k** Immunoblot of OXPHOS subunits expression from whole-cell lysates. TOM20 and Actin, loading controls. **l** Quantification of OXPHOS proteins relative to control, (*n* = 4, ***p = 0.0003 and 0.0001, ****p = 5.42 × 10⁻⁶). **m** DHE oxidation rate plotted as ROS production rate and normalised to Ctrl1, (*n* = 7, **p = 0.0015). Data in (**b**, **d**, **e**, **g–j**, **l**, **m**) are mean ± SD and individual data points represents independent experiments. Statistics were by one- or two-way ANOVA with Šídák or Holm–Šídák post hoc tests.

To characterise the impact of pathogenic *EPG5* variants on mitochondrial metabolism and bioenergetic function, we examined human dermal fibroblasts derived from patients (Supplementary Table 1) either carrying homozygous missense mutations (p.Gln336Arg, p.Arg1621Gln, hereafter referred to as patient 1 and patient 2, respectively) or compound heterozygous and homozygous truncating mutations (p.Arg299*/Pro1827Ala, p.Phe1604Glyfs*20, hereafter referred to as patient 3 and patient 4, respectively) as well as healthy control cell lines from donors matched for age and sex. Protein expression analysis by immunoblotting in control and patient fibroblasts revealed an almost complete absence of normal EPG5 protein in all patient cells (Fig. 1a, b), even in missense mutations, while in comparison the *EPG5* mRNA expression showed a significant reduction in patient cells (Supplementary Fig. 1a).

In order to assess mitochondrial bioenergetic function in patient fibroblasts, we first used equilibration of tetramethyl rhodamine methyl ester (TMRM) to measure ΔΨ_m_ by confocal imaging (Fig. 1c and Supplementary Fig. 1b). Single cell analysis of mitochondrial TMRM fluorescence intensity showed reduced ΔΨ_m_ in all the patient cells compared to controls (Fig. 1d). Quantitative analysis of mitochondrial volume occupancy by co-labelling the cells with Calcein AM, to measure cytosolic area, showed no change between the cell lines (Supplementary Fig. 1c). However, quantification of mtDNA copy number revealed a significant increase in mtDNA copy number in all patient fibroblasts (Supplementary Fig. 1d). Morphometric analysis of TMRM-labelled mitochondria showed that a large proportion of the mitochondrial network was fragmented while a small pool showed swollen morphology in patient cells (Fig. 1e). To investigate the cause of the reduced ΔΨ_m_ in patient fibroblasts, we measured the redox state of the mitochondrial NADH pool, the main substrate for the mitochondrial electron transport chain (ETC), using NAD(P)H autofluorescence imaging^50^. The resting level of NADH autofluorescence was quantified through the experimental determination of the “redox index,” a ratio of the signal representing the maximally oxidised pool (response to 1 μM FCCP) and the maximally reduced pool (the response to 1 mM NaCN) (Fig. 1f and Supplementary Fig. 1e). Patient fibroblasts exhibited a significant increase in the NADH redox state (i.e., reduced (NADH)/ oxidised  (NAD^+^)) in comparison to control cells, suggesting an impaired function of the mitochondrial ETC (Fig. 1g) consistent with the previous reports^3^. To confirm this, the oxygen consumption rate was measured using the Seahorse XFe96 extracellular flux analyser (Fig. 1h). Both the ATP-linked respiratory rate and spare respiratory capacity were substantially reduced in patient cells compared to controls (Fig. 1i, j).

To further identify the cause of the mitochondrial bioenergetic defect observed in the patient cells, steady-state levels of ETC components were analysed by SDS-PAGE (Fig. 1k) and the native assembly of these components into respiratory supercomplexes by BNGE and immunoblotting using the Total OXPHOS Antibody Cocktail, which contains antibodies against representative subunits of all five OXPHOS complexes (NDUFB8, SDHB, UQCRC2, MTCO1, and ATP5A). (Supplementary Fig. 1f). The expression levels of CI, CII and CIV were all significantly reduced as was the assembly of CI, CIII and CIV compared to control cells except for CV which remained unaffected in all patient cells (Fig. 1l and Supplementary Fig. 1g). Patient cells also showed an increased rate of production of intracellular reactive oxygen species (ROS), measured using dihydroethidium (DHE) fluorescence (Fig. 1m). Together, these data show an impaired mitochondrial bioenergetic function and a respiratory chain defect associated with the decreased expression and assembly of the OXPHOS complexes in patient fibroblasts with EPG5 deficiency.

### Pathogenic *EPG5* variants causes dysregulation of mitochondrial calcium signalling

**Fig. 2:**
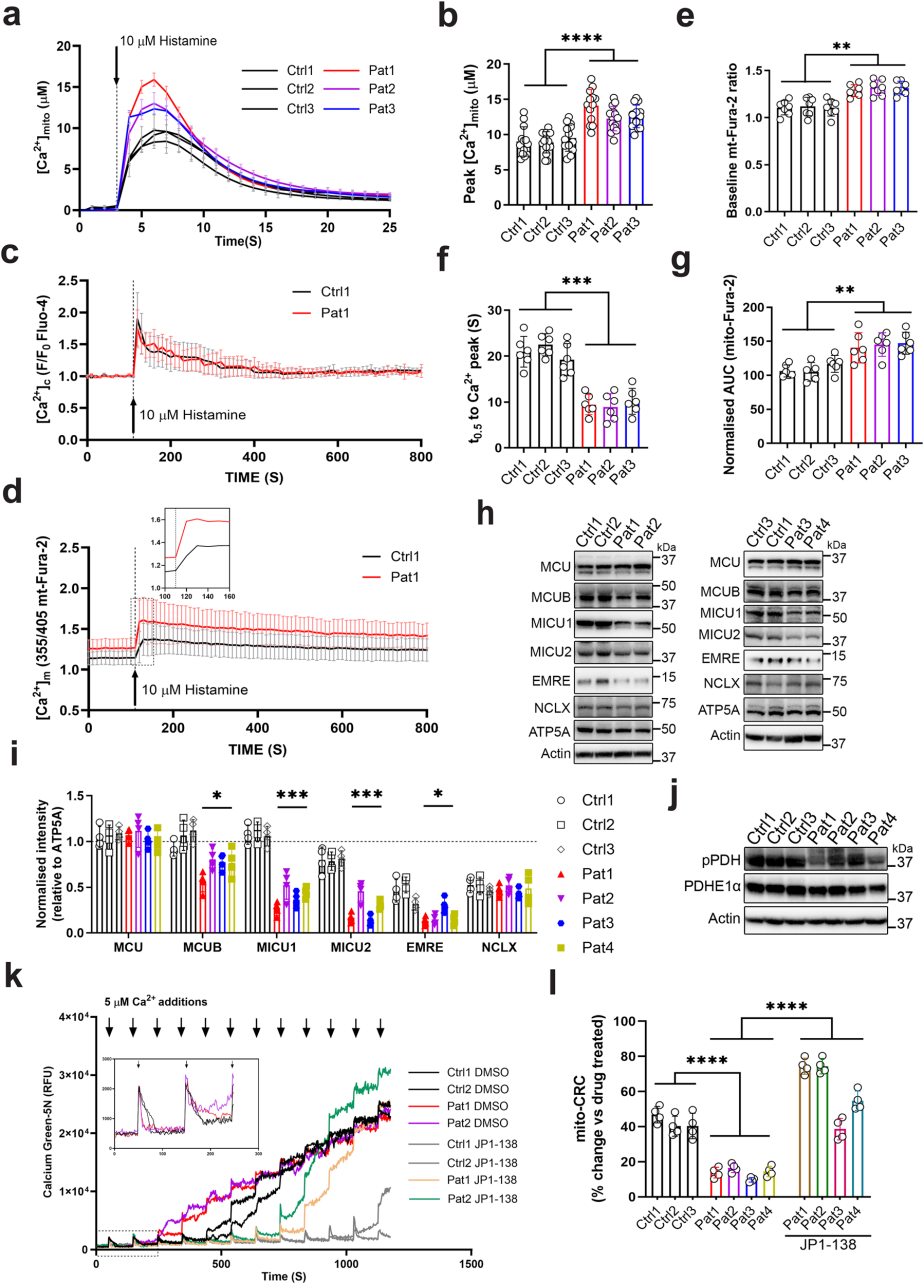
EPG5-mutant fibroblasts show impaired mitochondrial Ca^2+^ signalling. **a** Mean change ± SD in mitochondrial Ca^2+^ ([Ca^2+^]_m_) uptake measured by mt-AEQ in response to 10 μM histamine, (*n* = 15 runs). **b** Maximum [Ca^2+^]_m_ induced by 10 μM histamine in control and patient fibroblasts, (*n* = 15, ****p = 2.6 × 10⁻⁵). **c**, **d** Mean cytolsolic Ca^2+^ ([Ca^2+^]_c_) and [Ca^2+^]_m_ traces in Ctrl1 and Pat1 fibroblasts co-loaded with Fluo-4 AM and mito-Fura-2 AM after stimulation with 10 μM histamine, (*n* = 6 runs). **e** Resting [Ca^2+^]_m_ measured with mito-Fura-2 AM before histamine stimulation, (*n* = 6, **p = 0.0065). **f** Time to half-maximal [Ca^2+^]_m_ rise after histamine stimulation, (*n* = 6, ***p = 0.0001). **g** Area under the mito-Fura-2 AM traces, representing total [Ca^2+^]_m_ uptake over time (*n* = 6, **p = 0.0094). **h** Immunoblots of proteins involved in mitochondrial Ca^2+^ signalling in control and patient fibroblasts, ATP5A and Actin, loading controls. **i** Protein abundance relative to ATP5A, normalised to Ctrl1, (*n* = 4, *p = 0.0243 and 0.0105, ***p = 0.0002 and 0.0005). **j** Representative immunoblots of pPDH (PDH-E1α pS293) and total PDH (PDH-E1α). Actin, loading control. **k** Calcium retention capacity of isolated mitochondria from control and patient fibroblasts. Mean traces show extramitochondrial Ca^2+^ measured with Calcium Green-5N after repetitive 5 μM CaCl_2_ boluses in the presence or absence of JP1-138 (100 nM, added at 0 s), inset, Ca^2+^ uptake rate in control and patient mitochondria. **l** Mitochondrial Ca^2+^ retention capacity calculated as percentage inhibition relative to untreated mitochondria, (*n* = 4, ****p = 1.6 × 10⁻⁸, 3.73 × 10⁻¹³). Data in (**a–g**, **i**, **l)** are mean ± SD and data points represent independent experiments. Statistics were by one- or two-way ANOVA with Šídák or Holm–Šídák post hoc tests.

The Ca^2+^-dependent regulation of mitochondrial metabolism, mediated by Ca^2+^ entry into mitochondria through the MCU complex, plays a key role in driving respiratory chain activity that maintains the rate of ATP synthesis in response to increased energy demand^51^. As mitochondrial Ca^2+^ uptake is potential-dependent, we investigated whether impaired mitochondrial Ca^2+^ uptake due to the reduced ΔΨ_m_ might amplify the bioenergetic defect in the patient cells. We therefore measured the mitochondrial matrix [Ca^2+^]_m_ response in control and patient cells expressing mitochondria-targeted aequorin (mt-AEQ) following a challenge with 10 µM histamine (Fig. 2a). Surprisingly, despite the reduction in mitochondrial membrane potential, mitochondrial Ca^2+^ uptake was increased in patient cells with significantly larger peak of [Ca^2+^]_m_ upon physiological histamine stimulation (Fig. 2b). This striking finding suggested that the increase in histamine-induced [Ca^2+^]_m_ in patient fibroblasts could be due to altered endoplasmic reticulum (ER) Ca^2+^ content and ER-mitochondria proximity or dysregulation of the MCU/NCLX complex. To test this, cells were labelled with Fluo-4 AM and mito-Fura-2 AM^52^ to simultaneously monitor the changes in [Ca^2+^]_c_ and [Ca^2+^]_m_ (Fig. 2c, d). Application of 10 µM histamine to both cell types produced a comparable rise in [Ca^2+^]_c_ with no significant changes in total Ca^2+^ released from the ER (Supplementary Fig. 2a) or its clearance as measured by the time taken from peak [Ca^2+^]_c_ to half the final baseline value (Supplementary Fig. 2b). However, the increase in both resting and histamine-stimulated [Ca^2+^]_m_ was confirmed by ratiometric measurement of mito-Fura-2 intensity in patient cells (Fig. 2e–g). Notably, the initial rate of histamine-induced mitochondrial Ca^2+^ uptake was also significantly increased in patient fibroblasts (Fig. 2f). We further examined the ER-mitochondria contact sites by analysing the fraction of mitochondrial surface involved in contact with ER by transmission electron microscopy (TEM) (Supplementary Fig. 2c). We found no significant change in the frequency and total number of contacts in the 0-20 nm range, the gap width relevant for Ca^2+^ transfer, between both the cell types (Supplementary Fig. 2d).

The increased velocity of mitochondrial Ca^2+^ uptake observed in patient fibroblasts prompted us to measure the expression levels of regulatory components of the MCU complex (Fig. 2h). MCU and NCLX protein levels were not altered but surprisingly, the expression of MICU1, MICU2 and EMRE was significantly reduced in patient cells indicating that the ‘gatekeeper’ function of MICU1 and MICU2 in mitochondrial Ca^2+^ uptake is compromised in patient cells allowing rapid entry of Ca^2+^ into the mitochondria (Fig. 2i). We further tested whether ectopic expression of MICU1-HA in patient fibroblasts reduces resting [Ca^2+^]_m_ loading and increases the sensitivity to elevated [Ca^2+^]_c_ (Supplementary Fig. 2e, f). Ratiometric measurement of basal mito-Fura-2 intensity from patient 1 fibroblast showed an elevated mt-Fura-2 ratio consistent with the low MICU1 abundance and impaired gatekeeping function (Supplementary Fig. 2g). Ectopic expression of MICU1 substantially reduced basal mito-Fura-2 ratio to levels comparable to control fibroblasts (Supplementary Fig. 2h). To measure mitochondrial Ca²⁺ uptake in response to cytosolic Ca²⁺ elevations, control and patient fibroblasts co-expressing mt-AEQ were stimulated with histamine (Supplementary Fig. 2i). The histamine-evoked peak [Ca²⁺]_m_ was significantly lower in MICU1-expressing patient fibroblasts than in naïve patient cells (Supplementary Fig. 2j), indicating restoration of the MCU Ca²⁺ uptake threshold. Mitochondrial Ca^2+^ signalling directly impacts TCA cycle intermediates by the allosteric activation of pyruvate dehydrogenase (PDH) and α-ketoglutarate dehydrogenase (αKGDH). Accumulation of mitochondrial Ca^2+^ activates PDH phosphatase (PDP1), which dephosphorylates the PDH E1α subunit and thereby increases PDH activity to convert pyruvate to acetyl-CoA^28^. Immunoblotting analysis of phosphorylated PDH (p-PDH E1α, inactive) revealed a significantly reduced p-PDH E1α/PDH ratio in patient cells compared to controls (Fig. 2j and Supplementary Fig. 2k), consistent with the chronic elevation of resting [Ca^2+^]_m_ and enhanced PDP activity in EPG5-deficient patient cells. Remarkably, a high resting [Ca^2+^]_m_ and the associated mitochondrial bioenergetic deficiency are the characteristic cellular features of fibroblasts derived from patients with *MICU1* loss-of-function mutations, a rare childhood disorder characterised by proximal myopathy, cognitive impairment and a progressive neurodegeneration^53,54^.

Supraphysiological mitochondrial Ca^2+^ accumulation can trigger the opening of mPTP and Ca^2+^ dependent cell death^32^. We therefore asked whether diminished expression of the MCU regulatory proteins renders patient cells vulnerable to [Ca^2+^]_m_ overload and mPTP opening. To examine this, isolated mitochondria from control and patient fibroblasts were challenged with a series of 5 μM Ca^2+^ boluses to evoke [Ca^2+^]_m_ overload. We found that mitochondria from patient cells tolerated fewer Ca^2+^ pulses before the precipitous increase in fluorescence signal indicating mPTP opening (Fig. 2k). Consistent with the above results, the rate of Ca^2+^ uptake was also increased in patient cells in this assay, as measured by the time taken from peak Ca^2+^ to half the final baseline value (Supplementary Fig. 2l). Interestingly, preincubation with JP1-138, a highly specific novel mitochondrial cyclophilin D (CypD) targeting molecule and a potent inhibitor of PTP^55^, significantly increased the mitochondrial retention capacity in patient cells (Fig. 2k, l). We further examined whether MICU1 expression protects against [Ca²⁺]_m_ overload and mPTP opening. Isolated mitochondria from control and patient fibroblasts, with or without MICU1 expression, were challenged with sequential Ca²⁺ boluses (Supplementary Fig. 2m). Quantitative analysis of Calcium Green-5N fluorescence showed that mitochondria from MICU1-expressing patient fibroblasts tolerated significantly more Ca²⁺ pulses before mPTP opening (Supplementary Fig. 2n). Together, these data demonstrate that low abundance of MICU1 decreases the threshold for Ca^2+^ uptake and renders EPG5-deficient patient cells more vulnerable to [Ca^2+^]_m_ overload and mPTP opening.

### Mitochondrial calcium overload triggers mtDNA release in patient-derived fibroblasts

**Fig. 3:**
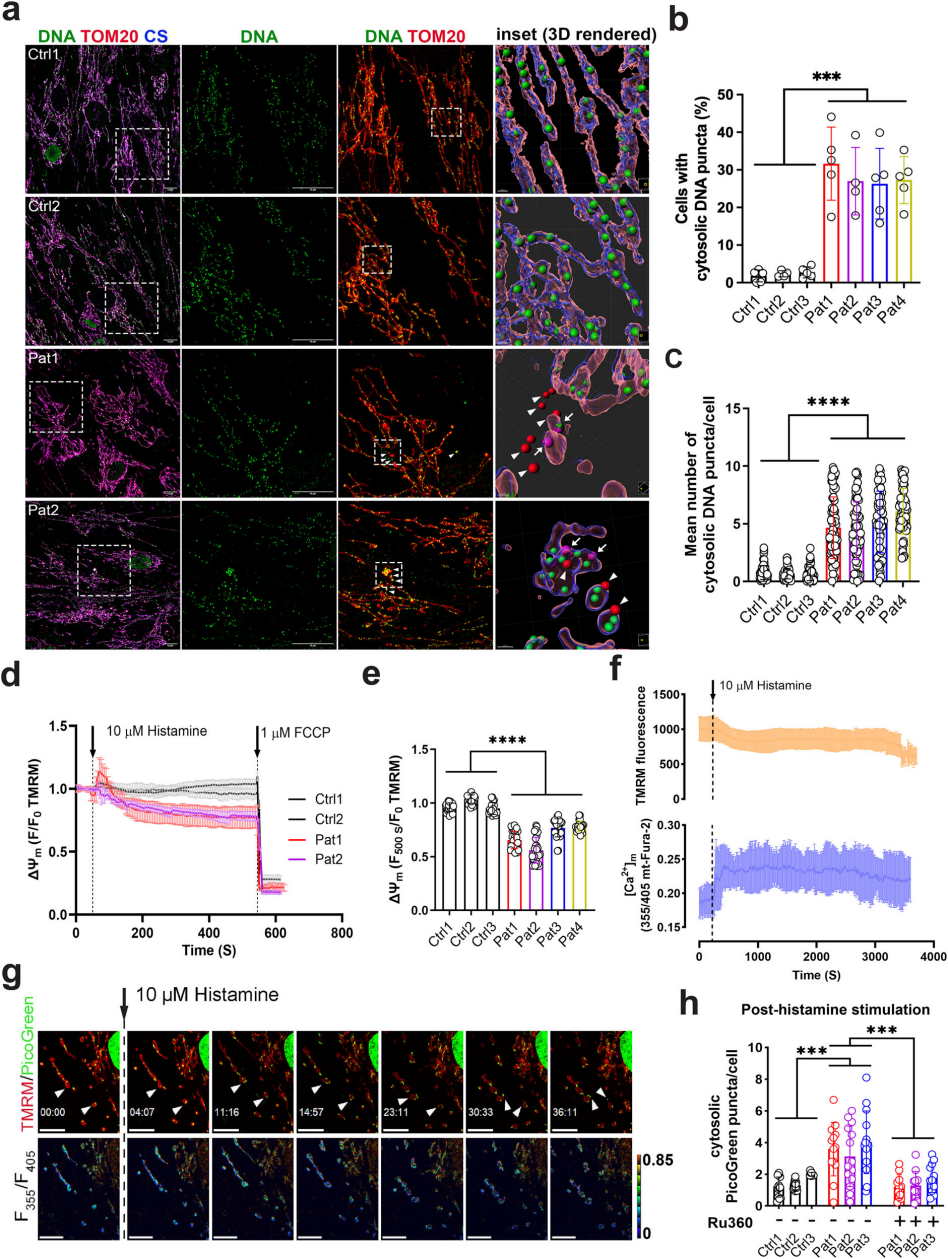
Mitochondrial Ca^2+^ overload induces cytosolic mtDNA release in patient fibroblasts. **a** Representative super-resolution Airyscan images of control and patient fibroblasts immunolabelled for DNA (green), TOM20 (outer mitochondrial membrane, red) and citrate synthetase (mitochondrial matrix, blue). Insets highlight regions where DNA does not co-localise with TOM20 in patient cells. Three-dimensional reconstructions show outer mitochondrial membrane (red), inner mitochondrial membrane (blue) and mtDNA (green). In control fibroblasts, mtDNA puncta are confined within mitochondria, whereas patient fibroblasts display cytosolic mtDNA puncta (arrowheads). mtDNA nucleoids partially externalised beyond the outer and inner mitochondrial membranes are shown in magenta and indicated by arrows. Scale bars, 10 μm (overview) and 0.5 μm (insets). **b** Percentage of control and patient fibroblasts containing cytosolic DNA puncta, (*n* = 5, ***p = 0.000861). **c** Quantification of cytosolic DNA puncta per cell, (*n* = 71 cells for Ctrl1–3 and 69, 70, 61 and 67 cells for Pat1–4, respectively, ****p = 3.03 × 10⁻^7^). **d** Mean change ± SD in TMRM fluorescence (ΔΨ_m_) in response to 10 μM histamine and FCCP-induced depolarisation in control and patient fibroblasts (*n* = 22 runs for Ctrl1–3 and 24 runs for Pat1–4). **e** Mitochondrial depolarisation measured 500 s after 10 μM histamine stimulation (*n* = 22 for Ctrl1–3 and 24 for Pat1–4, ****p = 5.02 × 10⁻^5^). **f** Mean traces of TMRM fluorescence intensity and [Ca^2+^]_m_ change following 10 μM histamine stimulation in Pat1 fibroblasts co-labelled with TMRM, mito-Fura-2 and PicoGreen, (*n* = 15 runs). **g** Time-lapse confocal snapshots of patient fibroblasts co-labelled with TMRM (red) and PicoGreen (green; upper panels) or mito-Fura-2 AM (ratiometric; lower panels), quantified in (**f**). Elapsed time after histamine stimulation is indicated; arrowheads denote mtDNA externalisation events (Supplementary movie 2). Scale bars, 5 μm. **h** Quantification of PicoGreen-positive mtDNA puncta released into the cytosol following 10 μM histamine stimulation, ± Ru360 pretreatment (*n* = 14 cells per group, ***p = 0.0006, 0.0003). Data in (**b**–**h**) are mean ± SD, points represent independent experiments. Statistics were performed using one- or two-way ANOVA with Šídák post hoc tests.

Pathological excessive mitochondrial Ca^2+^ uptake can irreversibly lead to bioenergetic collapse, mitochondrial swelling, and mPTP opening. This catastrophic loss of mitochondrial function is a major trigger for acute cell death^31^. Alternatively, limited mPTP opening in a subset of damaged mitochondria may lead to a chronic low-grade innate inflammatory response. Previous studies have suggested that mPTP opening may allow mtDNA release into the cytosol thus driving activation of innate immune signalling and inflammation, a disease hallmark of ALS and other chronic metabolic pathologies^46,56^. We therefore asked whether [Ca^2+^]_m_ overload in patient cells drives this pathway. Control and patient fibroblasts immunolabelled with anti-TOM20, anti-Citrate synthetase (CS) and DNA antibodies to label OMM, matrix and mtDNA nucleoids, respectively, were imaged using Airyscan microscopy which provides near super-resolution imaging, sufficient to visualise the integrity of the mitochondrial outer and inner membrane and mtDNA nucleoids (Fig. 3a). Both cell types displayed mtDNA nucleoids contained within a continuous matrix and OMM colocalizing together (Supplementary Fig. 3a). However, we found cytosolic localisation of DNA puncta in over 30% of patient cells as well as a significant increase in the average cytosolic DNA puncta per cell but none at all in control cells (Fig. 3b, c). Notably, patient cells also displayed a fragmented mitochondrial network with swollen morphology (Fig. 3a). The presence of cytosolic DNA puncta in patient cells under normal conditions supported our hypothesis that increased resting [Ca^2+^]_m_ in patient cells could cause chronic mtDNA release.

To recapitulate the progressive chain of events which could lead to [Ca^2+^]_m_ overload-induced mtDNA release, we first monitored ΔΨ_m_ in both control and patient fibroblasts following challenge with 10 µM histamine (Fig. 3d). Control cells maintained ΔΨ_m_ over time until the uncoupler-induced loss of ΔΨ_m_. In contrast, patient cells exhibited a steep decrease in membrane potential after the histamine challenge leading to more than a 30% reduction in ΔΨ_m_ over time (Fig. 3e). Loss of ΔΨ_m_ is one of the hallmark features of [Ca^2+^]_m_ overload-induced mPTP opening. Therefore, we next co-labelled the cells with TMRM, mito-Fura-2 and PicoGreen (a potential-dependent DNA binding probe) to monitor the intra-mitochondrial dynamics of ΔΨ_m_, [Ca^2+^]_m_ and mtDNA respectively, over a prolonged time interval of 30 min to 1 h. Live-cell imaging of control fibroblasts following histamine challenge showed a slow increase in [Ca^2+^]_m_ which coincided with a small increase in ΔΨ_m_ which was maintained over time (Supplementary Fig. 3b,c). An increased fragmentation of the mitochondrial network was also observed after histamine stimulation (Supplementary movie 1 and Supplementary Fig. 3d) suggesting a Ca^2+^-induced remodelling of mitochondrial morphology as previously reported^57^. In comparison to control cells, histamine challenge in patient cells caused a rapid increase in peak [Ca^2+^]_m_ followed by a simultaneous fall in ΔΨ_m_ and mitochondrial swelling (Fig. 3f, g and Supplementary movie 2). Analyses of individual mitochondria in patient cells revealed the release of PicoGreen-labelled mtDNA events that coincided with the depolarisation and swelling of mitochondria (Fig. 3h) that were never observed in control cells.

To determine whether blocking mitochondrial Ca²⁺ uptake could prevent cytosolic mtDNA release, patient fibroblasts were pretreated with Ru360, a potent and selective MCU inhibitor^58^. Ru360 pretreatment markedly attenuated both the rate and amplitude of histamine-induced mitochondrial Ca²⁺ uptake in control and patient fibroblasts (Supplementary Fig. 3e-g), confirming effective inhibition of MCU-dependent Ca²⁺ entry. Under these conditions, live cell imaging of Ru360-treated patient fibroblasts co-labelled with TMRM, mito-Fura-2, and PicoGreen revealed a strongly attenuated mitochondrial Ca^2+^ signal accompanied by a small, sustained decrease in ΔΨ_m_ following histamine stimulation (Supplementary Fig. 3h, i and Supplementary movie 3). Quantitative analysis of individual mitochondria showed a substantial reduction in the frequency of cytosolic mtDNA release under these conditions (Fig. 3h). Together, these data indicate that mitochondrial Ca²⁺ overload is a key trigger of cytosolic mtDNA release and demonstrate a progressive cascade of events starting from uncontrolled [Ca^2+^]_m_ uptake and overload to loss of ΔΨ_m_ and mitochondrial swelling that culminates in mPTP opening and mtDNA release.

### mtDNA release drives cGAS-STING activation and interferon response in patient fibroblasts

**Fig. 4:**
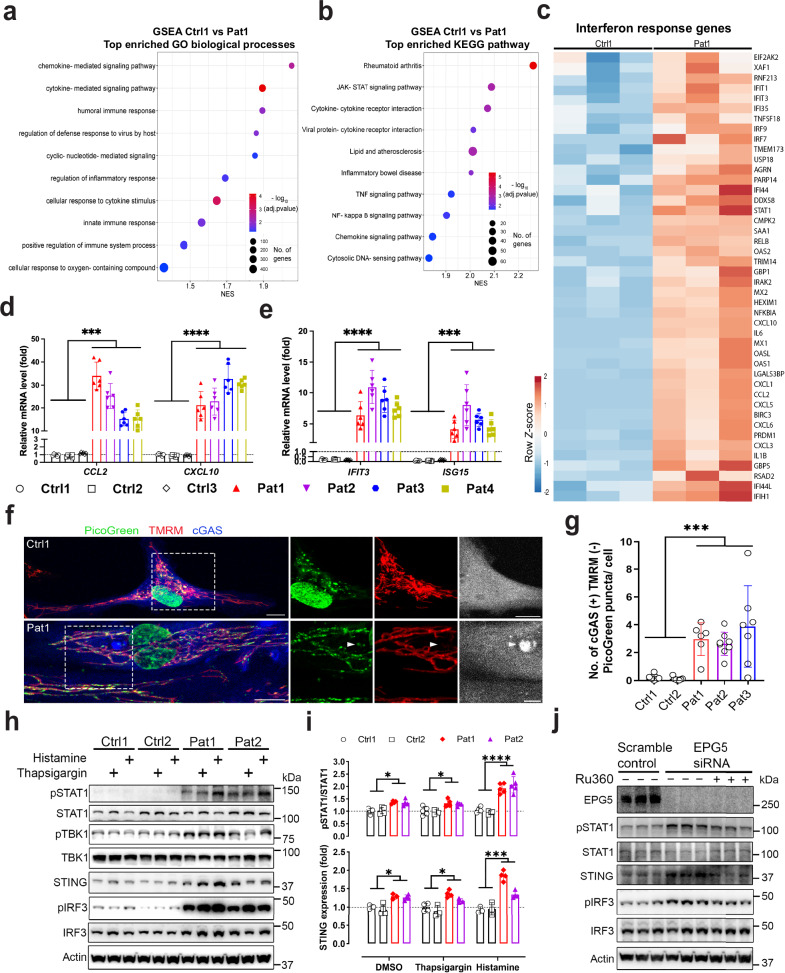
Activation of cGAS-STING pathway and interferon response triggered by cytosolic release of mtDNA in patient fibroblasts. **a**, **b** Pathway analysis of RNA-seq data from Pat1 compared to Ctrl1 fibroblasts. Dot plots and gene set enrichment analysis (GSEA) were performed using Gene Ontology (GO) biological process and KEGG pathway gene sets. Adjusted *p* values were corrected for multiple testing using the Benjamini–Hochberg method, NES, normalised enrichment score. **c** Heatmap of RNA-seq data showing the top 50 upregulated differentially expressed type I/III interferon (IFN) genes in Ctrl1 and Pat1 fibroblasts, (*n* = 3). **d**, **e** qRT–PCR analysis of interferon-stimulated gene expression across all control and patient fibroblasts, (*n* = 5, 6, ***p = 0.000103, 0.000130, ****p = 0.000021, 0.000045). **f** Representative confocal images of Ctrl1 and Pat1 fibroblasts transfected with BFP–cGAS and co-labelled with TMRM (red) and PicoGreen (green). Insets shows cytosolic mtDNA nucleoids lacking TMRM signal that co-localise with cGAS puncta (grey) in patient cells (arrowheads). Scale bars, 10 μm (overview) and 5 μm (insets). **g** Quantification of cGAS-positive, TMRM-negative PicoGreen puncta per cell, (*n* = 10, ***p = 0.0004). **h** Immunoblots of STING-dependent type-I IFN signalling proteins from whole-cell lysates of control and patient fibroblasts treated with 10 μM histamine and 1 μM thapsigargin for 24 h. Actin, loading control. **i** Ratio of phosphorylated STAT1 (pTyr701) to total STAT1 normalised to the Ctrl1 ratio, (*n* = 4, *p = 0.0124 and 0.0161, ****p = 0.000007) and STING protein abundance normalised to control, (*n* = 4, *p = 0.0191, 0.0155, ***p = 0.0006). **j** Representative immunoblots of STING-dependent interferon signalling proteins from hTERT-immortalised control and *EPG5*-siRNA fibroblasts treated with 10 μM Ru360 for 12 h. Actin, loading control. Data in (**d**, **e**, **g**, **i**) are expressed as mean ± SD and data points represents independent experiments. Statistics were performed using one- or two-way ANOVA with Šídák or Holm–Šídák post hoc tests.

To characterise the cell-intrinsic changes in gene expression which may impact cellular function after mtDNA release, we performed bulk RNA sequencing (RNA-seq) on a control (control 1) and a pair of patient fibroblasts (patient 1 and patient 3). Gene set enrichment analysis (GSEA) of the gene signature enriched in patient fibroblasts revealed the top upregulated genes involved in the immune response (Fig. 4a, b and Supplementary Fig. 4a, b). Among the most significantly upregulated pathways, nine from the Gene Ontology biological process and seven from the KEGG pathway are related to immune activation and response. A gene expression profile of the differentially expressed genes associated with the enriched pathways, collected using a significance level of false discovery rate (FDR) < 0.05, uncovered a striking upregulation of the type I/III IFN signature gene set in the patient fibroblasts (Fig. 4c and Supplementary Fig. 4c). Among the list of 45 IFN-stimulated genes (ISGs), we observed an upregulation of the genes involved in direct antiviral activity (*IFIT1*, *IFIT3*, *IFI44*, *OASL*), members of the ISG transcription factors (*IRF9* and *STAT1*) which are activated downstream of type I/III IFN receptor and chemokines (*CCL2* and *CXCL10*) and pro-inflammatory cytokines (*TNF* and *IL-1B*) involved in adaptive immune response. Upregulation of these ISGs and pro-inflammatory genes was also confirmed in the remaining patient fibroblasts with pathogenic *EPG5* variants (Fig. 4d, e and Supplementary Fig. 4d) suggesting that the induction of the IFN response pathway is likely a common feature of EPG5 deficiency.

To investigate the signalling events upstream of IFN induction in patient fibroblasts, we asked if the presence of cytosolic mtDNA could induce type I IFN by activating the canonical cGAS-STING pathway. cGAS is predominantly a nuclear protein, however, immunofluorescence analysis revealed a significant increase in the intensity of the cytosolic cGAS in patient cells (Supplementary Fig. 4e, f) indicating its cytosolic relocalization in response to immunogenic intracellular DNA^59^. Live-cell imaging of fibroblasts over-expressing BFP-cGAS and co-labelled with TMRM and PicoGreen showed cytosolic cGAS puncta colocalizing with free mtDNA devoid of mitochondria in patient cells (Fig. 4f, g) supporting the notion that cytosolic cGAS observed in patient cells binds to mtDNA after its release from mitochondria. Cytosolic detection of mtDNA by cGAS activates classic STING signalosomes at the Golgi apparatus which in turn activates TBK1, a central kinase involved in the integration of innate immune signals from cytosolic DNA/RNA sensors to IFN induction by activating interferon-regulatory factors (IRFs)^60^. In control and patient fibroblasts treated with histamine or thapsigargin, to increase [Ca^2+^]_c_ and amplify [Ca^2+^]_m_ overload, immunoblotting for active pTBK1 showed a substantial increase both under resting and histamine/thapsigargin-stimulated conditions in patient compared to control cells (Fig. 4h and Supplementary Fig. 4g, h). Consistent with TBK1 activation, a significant increase in STING expression was also detected in patient fibroblasts which further increased with histamine challenge (Fig. 4i and Supplementary Fig. 4k). Notably, we observed a robust activation of downstream transcription factors IRF3 and STAT1 in patient cells under resting condition which increased by more than two-fold after histamine challenge (Fig. 4i and Supplementary Fig. 4i, j). To directly assess the contribution of mitochondrial Ca²⁺ uptake to STING-dependent type I IFN signalling, *EPG5* was knocked down in hTERT-immortalised skin fibroblasts, and mitochondrial Ca²⁺ entry was inhibited using Ru360 (Fig. 4j). Compared with control cells, *EPG5*-knockdown (*EPG5*-siRNA) fibroblasts displayed strong activation of IRF3 and STAT1-mediated signalling, accompanied by increased STING expression. Importantly, Ru360 treatment significantly attenuated IRF3 and STAT1 activation and reduced STING protein levels in *EPG5*-siRNA cells (Supplementary Fig. 4l–n). In parallel, qRT-PCR analysis demonstrated a significant reduction in the expression of interferon-stimulated genes (ISGs) downstream of STING-dependent type I IFN signalling following Ru360 treatment (Supplementary Fig. 4o, p). Together, these results support the RNA-Seq and mRNA expression data and indicate that cGAS-STING signalling is constitutively active in patient-derived fibroblasts and is further amplified by [Ca^2+^]_m_ overload during physiological Ca^2+^ signalling.

### mPTP inhibition or EPG5 rescue reduces STING-dependent IFN signalling in EPG5-deficient fibroblasts

Given that the constitutive activation of cGAS-STING signalling drives the downstream IFN pathway in patient fibroblasts, we reasoned that STING inhibition might curtail this inflammatory response. Treatment with H-151, a covalent inhibitor that blocks activation-induced palmitoylation of STING^61^, dampened the IFN response signalling in patient cells by reducing STING levels and normalising active IRF3 and STAT1 comparable to the levels seen in control cells (Supplementary Fig. 5a–d). However, treatment with G140, a cGAS-specific inhibitor^62^ showed only a marginal effect on STING, pIRF3 and pSTAT1 levels in patient cells (Supplementary Fig. 5a–d). Furthermore, H-151 treatment also substantially reduced the expression levels of ISGs in patient cells (Supplementary Fig. 5e, f) indicating that STING inhibition might represent a potential therapeutic target in patients with pathogenic *EPG5* variants.

We next asked whether the STING-IFN response pathway might act in a feedback manner to further impair mitochondrial bioenergetic function in patient fibroblasts. Patient fibroblasts exhibited no significant change in resting ΔΨ_m_ after 24 h treatment with H-151 (Supplementary Fig. 5g). Similarly, the NADH redox index remained unaltered in all the patient cells (Supplementary Fig. 5h). Long-term treatment with H-151 for three days failed to show any significant increase in resting and maximal respiration compared to untreated patient cells (Supplementary Fig. 5i–l). These results suggest that the inflammatory signature observed in patient fibroblasts is a consequence of impaired mitochondrial Ca^2+^ homoeostasis but does not contribute to the underlying bioenergetic defect of *EPG5*-related disorders.

**Fig. 5:**
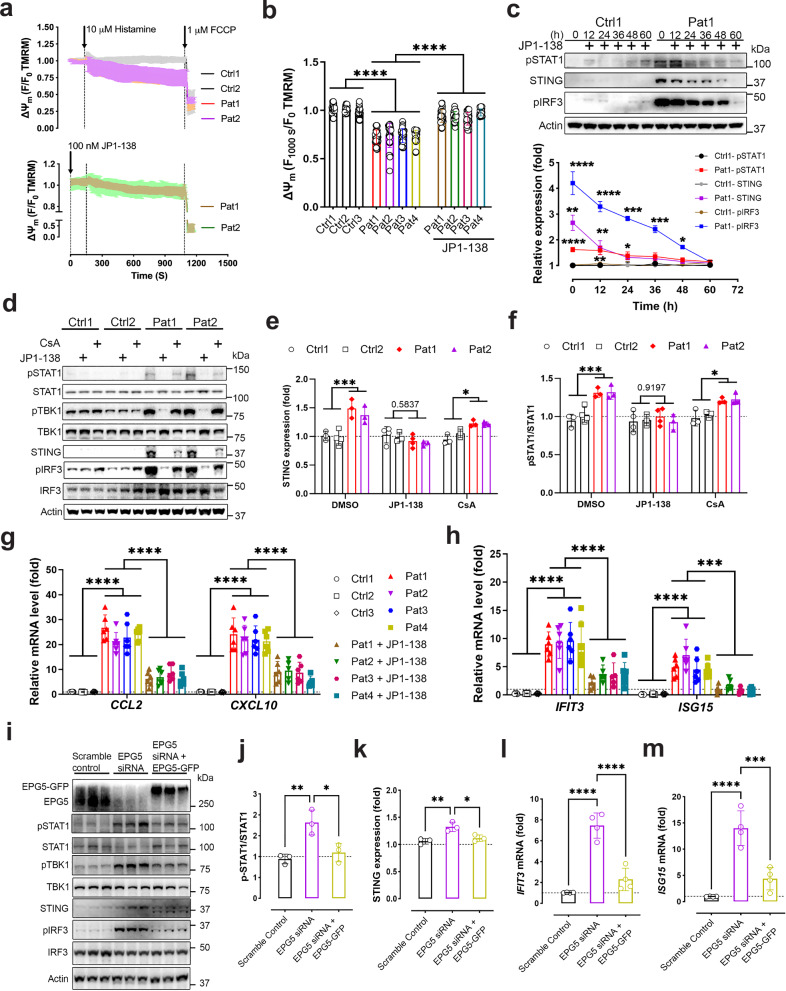
Attenuation of STING-dependent interferon response by JP1-138 treatment in patient fibroblasts. **a** Mean change ± SD in ΔΨ_m_, measured by TMRM fluorescence in response to 10 μM histamine challenge and FCCP-induced depolarisation in the absence (upper) and presence of JP1-138 (100 nM) (lower) in control and patient fibroblasts, (*n* = 20 runs). **b** Quantification of mitochondrial depolarisation at 1000 s after histamine stimulation, (*n* = 20, ****p = 1.44 × 10⁻⁸, 1.43 × 10⁻⁹). **c** Immunoblots of cGAS-STING signalling proteins from Ctrl1 and Pat1 fibroblasts treated with JP1-138 (100 nM) for the indicated times. Actin, loading control. Relative abundance of phosphorylated STAT1 (pTyr701), STING and phosphorylated IRF3 (pSer396) normalised to control, (*n* = 3, ****p = 4.9 × 10⁻⁵, **p = 0.0049, *p = 0.0235, **p = 0.0090, 0.0013, ****p = 3.03 × 10⁻⁹, 1.15 × 10⁻⁹, ***p = 0.0002, 0.0007, *p = 0.0361). **d** Immunoblots of STING-dependent type-I IFN signalling proteins from control and patient fibroblasts treated with CsA (1 μM) or JP1-138 (100 nM) for 3 days. Actin, loading control. **e** Quantification of STING protein levels normalised to control, (*n* = 4, *p = 0.0265, ***p = 0.0004)**. f** Ratio of pSTAT1 (p Tyr701) to total STAT1 normalised to Ctrl1 ratio, (*n* = 4, *p = 0.0197, ***p = 0.0007). **g**,**h** qRT–PCR analysis of interferon-stimulated gene expression in control and patient fibroblasts ± JP1-138 (100 nM) for 3 days (*n* = 6, ****p = 4.89 × 10^-12^, 2.14 × 10^-8^, 8.22 × 10^-10^, 6.25 × 10^-7^, 2.93 × 10^-7^, 4.93 × 10^-5^, 7.83 × 10^-9^, ***p = 0.0001). **i** Immunoblots of STING-dependent IFN signalling proteins from control siRNA and *EPG5*-siRNA fibroblasts ± EPG5-GFP nucleofection. Actin, loading control. **j**, **k** Quantification of pSTAT1/STAT1 ratio (*n* = 3, *p = 0.0180, **p = 0.0074) and STING protein abundance normalised to control siRNA, (*n* = 3, *p = 0.0214, **p = 0.0071). **l**, **m** qRT–PCR analysis of interferon-stimulated genes in control siRNA and *EPG5* siRNA fibroblasts ± EPG5-GFP nucleofection, (*n* = 4, ****p = 1.27 × 10^-5^, 7.59 × 10^-5^, 5.32 × 10^-5^, ***p = 0.0005,). Data in (**a–c**, **e**, **f**, **g**, **h**, **j–m**) are mean ± SD and points represent independent experiments. Statistics were performed using one-way ANOVA with Tukey’s test or two-way ANOVA with Holm–Šídák post hoc tests.

Based on our observation that mPTP inhibition by JP1-138 led to more than 50% increase in mitochondrial calcium retention capacity of patient cells (Fig. 2k, l), we hypothesised that treatment with JP1-138 could also suppress downstream activation of the STING-dependent type I IFN signalling by protecting patient cells from [Ca^2+^]_m_ overload and mtDNA release. To test this, we again monitored ΔΨ_m_ in both control and patient fibroblasts following a challenge with 10 µM histamine (Fig. 5a and Supplementary Fig. 6a). As expected, patient cells showed a rapid loss of ΔΨ_m_ in comparison to control cells. However, preincubation with JP1-138 completely rescued the histamine-induced mitochondrial depolarisation in patient cells (Fig. 5b) suggesting that JP1-138 treatment increases [Ca^2+^]_m_ buffering capacity in intact patient fibroblasts. Next, control and patient fibroblasts were treated with JP1-138 for different time points and immunoblotted to detect changes in the activation of the STING-dependent IFN signalling (Fig. 5c). Interestingly, JP1-138 treatment was associated with a time-dependent decrease in the expression of STING, pIRF3 and pSTAT1 reaching levels seen in control cells after 60 h. Using this time point, we further tested the efficacy of JP1-138 in supressing STING signalling by comparing it to another classic mPTP inhibitor, cyclosporin A (CsA) (Fig. 5d and Supplementary Fig. 6b). Treatment with JP1-138 (100 nM) restored the activation of TBK1, IRF3 and STAT1 and reduced the expression of STING to normal levels in patient cells (Fig. 5e, f and Supplementary Fig. 6c–f). In comparison, CsA treatment showed only a partial response and failed to effectively suppress the STING signalling when used at a higher concentration (1 µM) demonstrating the specificity and potent activity of JP1-138 in comparison to CsA. Consistent with these findings, JP1-138 treatment also significantly reduced the expression levels of ISGs in patient cells (Fig. 5g, h).

To further corroborate these results, we examined whether ectopic restoration of EPG5 expression attenuates STING-dependent IFN signalling in *EPG5*-knockdown immortalised fibroblasts (Fig. 5i). As expected, EPG5 depletion led to increased TBK1-dependent STING expression and the downstream activation of IRF3 and STAT1. Importantly, ectopic expression of EPG5-GFP reversed these effects, normalising STING levels and reducing IRF3 and STAT1 activation (Fig. 5j, k and Supplementary Fig. 6g, h). Consistent with these changes, mRNA levels of ISGs were significantly reduced in *EPG5*-siRNA cells upon re-expression of wild-type EPG5 (Fig. 5l, m and Supplementary Fig. 6i–k). Together, these data show that pharmacological inhibition of mPTP opening with JP1-138 protects EPG5-deficient patient cells from [Ca^2+^]_m_ overload-associated mitochondrial dysfunction and downstream activation of STING-dependent inflammatory signalling. In parallel, restoration of EPG5 expression is sufficient to suppress aberrant STING-dependent type-I IFN signalling in EPG5-deficient fibroblasts.

### JP1-138 suppresses mtDNA release and improves mitochondrial bioenergetic function in patient fibroblasts

**Fig. 6:**
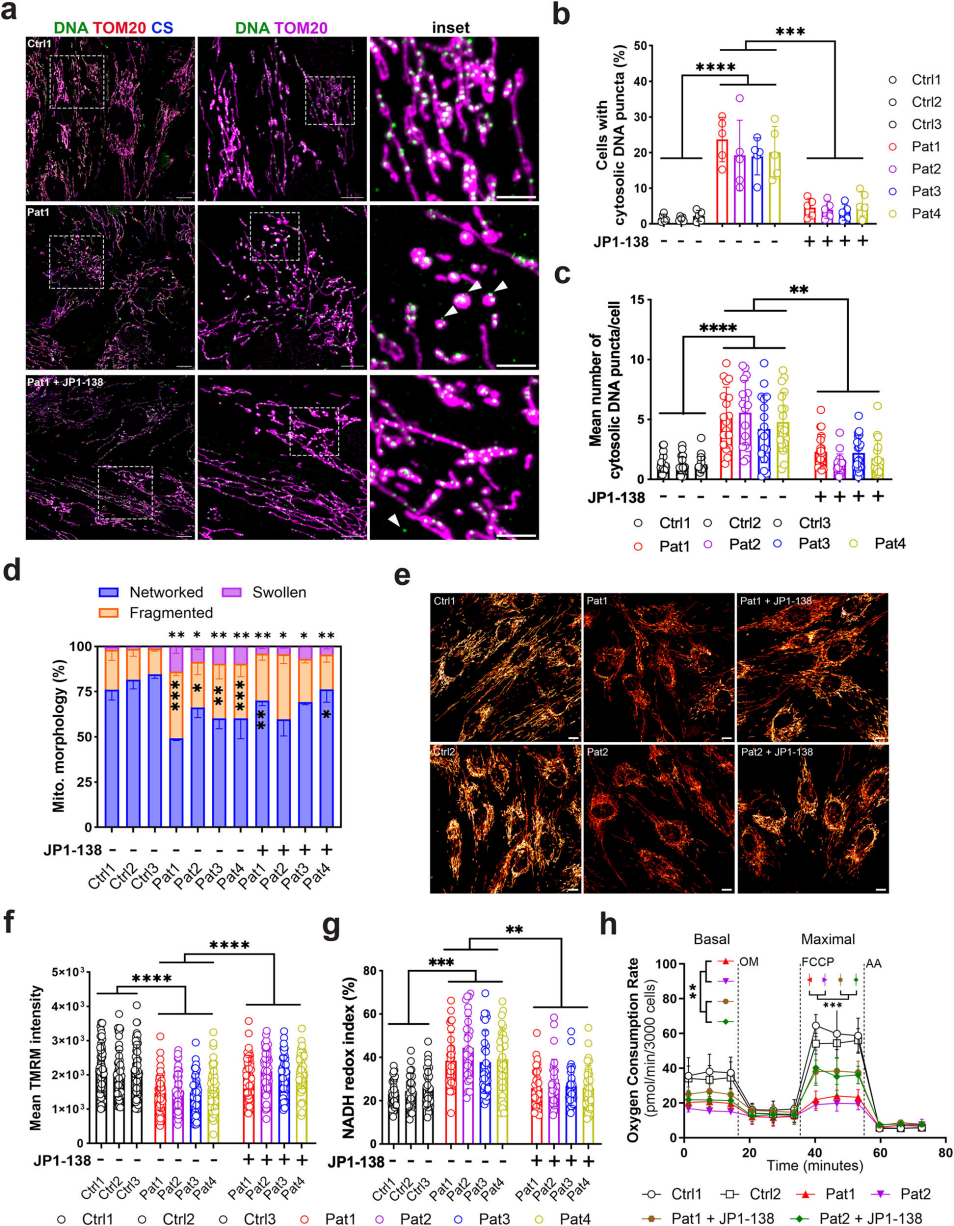
JP1-138 treatment reduces cytosolic mtDNA release and improves mitochondrial bioenergetic function in patient fibroblasts. **a** Representative Airyscan images of Ctrl1 and Pat1 fibroblasts treated with JP1-138 (100 nM) or DMSO for 3 days and immunolabelled for DNA (green), TOM20 (red) and citrate synthase (blue). Insets highlight regions in patient cells where DNA does not co-localise with TOM20 (pseudocolored magenta, white arrowheads) which is reduced by JP1-138 treatment. Scale bars, 10 μm (overview), 5 and 2.5 μm (insets). **b** Percentage of control and patient fibroblasts with cytosolic DNA puncta after JP1-138 treatment, (*n* = 5, ***p = 0.0001, ****p = 2.99 × 10⁻⁵). **c** Quantification of cytosolic DNA puncta per cell (*n* = 20 cells for each control line, 18 cells for each patient line and 19 cells for each patient line treated with JP1-138, **p = 0.0021, ****p = 9.65×10^−5^). **d** Mitochondrial morphology classified as networked, fragmented or swollen and expressed as percentage of total mitochondria, (*n* = 3, *p = 0.0140, 0.0177, 0.0327, 0.0436, 0.0418, **p = 0.0028, 0.0031, 0.0032, 0.0023, 0.0065, 0.0038, 0.0015, ***p = 0.0006, 0.0004). **e** Representative TMRM images of control and patient fibroblasts treated with JP1-138 (100 nM) or DMSO for 3 days. Scale bars: 20 μm. **f** Mean TMRM values showing steady state ΔΨ_m_, (*n* = 60 cells for each control line, 58 cells for each patient line and 56 cells for each patient line treated with JP1-138, ****p = 9.73 × 10⁻⁵, 4.32 × 10⁻⁵). **g** NADH redox index in control and patient fibroblasts treated with JP1-138 (100 nM) or DMSO for 3 days (*n* = 30 cells for each control line, 32 cells for each patient line with and without JP1-138, **p = 0.00232, ***p = 0.000614). **h** Normalised OCR traces from control and patient fibroblasts treated with JP1-138 (100 nM) or DMSO for 3 days (*n* = 6 runs, **p = 0.0077, ***p = 0.0009). Data in (**b**–**d**, **f–h**) are mean ± SD and points represent independent experiments. Statistical analysis was carried out using one/two-way ANOVA with Šidák or Holm-Šidák test.

To determine whether inhibition of mPTP opening by JP1-138 treatment or EPG5 expression also supressed cytosolic mtDNA release, fibroblasts immunolabelled with TOM20, DNA and CS were examined using Airyscan imaging (Fig. 6a and Supplementary Fig. 7a, b). As expected, both *EPG5*-siRNA cells and patient fibroblasts exhibited a marked increase in the cytosolic localisation of DNA puncta as well as an increased proportion of the mitochondrial pool with fragmented and swollen morphology observed in patient fibroblasts. Swollen mitochondria often displayed mtDNA extrusion in patient cells. Long term treatment with JP1-138 or ectopic expression of EPG5 lead to a marked reduction in both the average number and the total percentage of cells with cytosolic DNA puncta in patient fibroblasts and *EPG5*-siRNA cells (Fig. 6b, c and Supplementary Fig. 7c, d).

Notably, morphometric analysis of mitochondrial form following JP1-138 treatment revealed a significant reduction in the fraction of swollen mitochondria in patient fibroblasts (Fig. 6d). This pronounced remodelling of mitochondrial morphology was accompanied by a restoration of mitochondrial membrane potential, with resting ΔΨ_m_ in patient cells increasing to levels comparable to those observed in control fibroblasts after long-term JP1-138 treatment, indicating substantial mitochondrial repolarization (Fig. 6e, f). The increase in ΔΨ_m_ was accompanied by a decrease in the NADH redox index in comparison to untreated patient fibroblasts (Fig. 6g). Improvement in these bioenergetic parameters was associated with a concomitant increase in mitochondrial respiration in patient fibroblasts (Fig. 6h and Supplementary Fig. 7e). Long-term treatment with JP1-138 led to a small but significant increase in basal or ATP-linked respiration rate along with a large increase in the spare respiratory capacity when compared to the untreated patient group (Supplementary Fig. 7f, g). However, compared to the changes in ΔΨ_m_ and NADH redox index, the increase in mitochondrial respiration after JP1-138 treatment was partial when compared with untreated control fibroblasts. Together these data show that JP1-138 treatment effectively suppresses mtDNA release and partially rescues mitochondrial morphology and bioenergetic function in EPG5-deficient patient fibroblasts.

### Impaired mitochondrial Ca^2+^ signalling sensitises Q336R iPSC-derived neurons to excitotoxicity

**Fig. 7:**
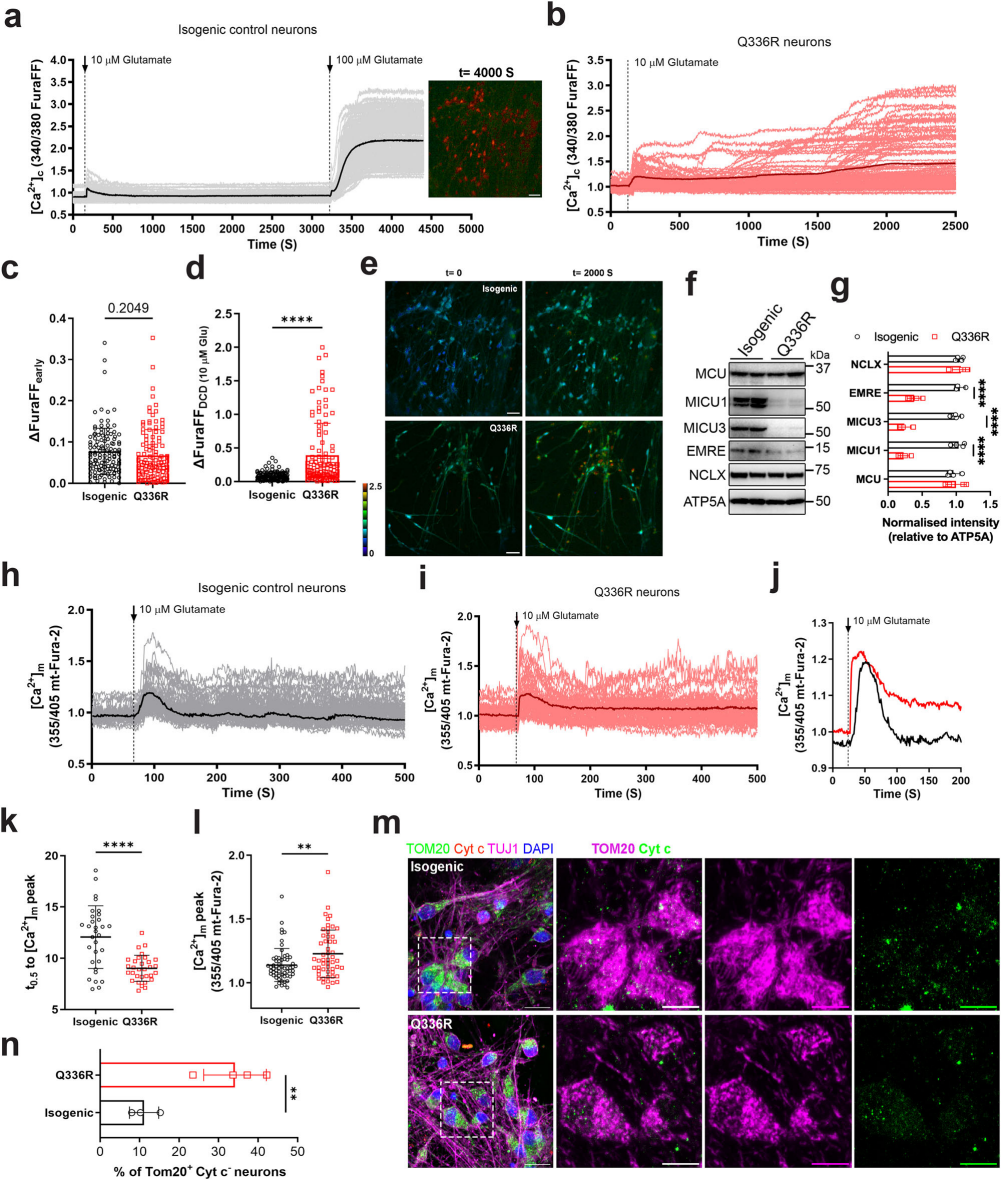
Enhanced susceptibility to glutamate-induced delayed Ca^2+^ dysregulation and cell death in Q336R neurons. **a**, **b** Cytosolic calcium ([Ca^2+^]_c_) in FuraFF AM-loaded neurons measured by fluorescence imaging over indicated intervals. Individual and mean traces show responses of isogenic and Q336R neurons to sequential application of 10 μM and 100 μM glutamate, (*n* = 91 and 120 neurons, respectively). **c** Baseline-subtracted peak of the early response at ~150 s after 10 μM glutamate, (*n* = 122 neurons). **d** Baseline-subtracted peak of delayed Ca^2+^dysregulation (DCD) at 2000 s after 10 μM glutamate, (*n* = 124 neurons, ****p = 2.39 × 10^-10^). **e** Representative FuraFF ratio at *t* = 0 s and 2000 s after glutamate exposure, showing recovery of [Ca^2+^]_c_ in isogenic neurons but sustained elevation in Q336R neurons, consistent with DCD. Scale bars: 50 μm. **f** Immunoblots of proteins involved in mitochondrial Ca^2+^ signalling in whole-cell lysates from isogenic and Q336R neurons. ATP5A, loading control. **g** Protein abundance relative to ATP5A, normalised to isogenic control, (*n* = 4, ****p = 3.59 × 10^-9^, 3.93 × 10^-11^, 6.45 × 10^-12^). **h**, **i** Mitochondrial Ca^2+^ ([Ca^2+^]_m_) in mito-Fura-2 AM-loaded neurons after 10 μM glutamate. Individual traces are shown for isogenic and Q336R neurons, (*n* = 55 and 75 neurons for isogenic and Q336R respectively). **j** Mean [Ca^2+^]_m_ traces calculated from (**h**, **i**). **k** Time to half-maximal [Ca^2+^]_m_ rise after glutamate stimulation, (*n* = 32 neurons, ****p = 3.34 × 10^-6^). **l** Maximum [Ca^2+^]_m_ rise induced by 10 μM glutamate, (*n* = 61 neurons, **p = 0.0041). **m** Representative confocal images of isogenic and Q336R neurons treated with 10 μM glutamate or PBS for 12 h and labelled for TOM20, cytochrome c (Cyt c), and β-tubulin III (TUJ1). Insets show regions lacking co-localisation of TOM20 (pseudocolored magenta) and Cyt c (pseudocolored green) in Q336R neurons. Scale bars: 20 μm (overview), 5 μm (insets). **n** Percentage of total mitochondrial area lacking Cyt c, (*n* = 4, **p = 0.0059). Data in (**c**, **d**, **g**, **k**, **l**, **n**) are mean ± SD with individual data points from independent experiments. Statistics were by Welch’s unpaired two-tailed *t*-test or two-way ANOVA with Šidák test.

To investigate the impact of impaired mitochondrial bioenergetic function and mitochondrial Ca^2+^ signalling more specifically on the pathophysiology of neurodegeneration that is a severe feature of patients with *EPG5*-related disorders, we used an iPSC-derived cortical neuronal model, reprogrammed from patient 1 fibroblasts bearing the pathogenic founder Q336R missense mutation and a CRISPR/Cas9-edited isogenic control. In vitro neurodifferentiation of isogenic and Q336R iPSCs yielded mixed cultures of neurons and glial cells with no significant change in the proportion of cell types (Supplementary Fig. 8a, b). The increased bioenergetic demand imposed during metabolic workload, such as physiological glutamate-induced excitation may sensitise neurons with impaired mitochondrial function to glutamate excitotoxicity^17^. To test this, mixed cultures of neurons and glial cells, loaded with low-affinity [Ca^2+^]_c_ indicator FuraFF-AM were challenged with near physiological (10 µM) and toxic (100 µM) glutamate concentrations (Fig. 7a, b). Early peak [Ca^2+^]_c_ (ΔFuraFF_early_) remained unchanged between isogenic and Q336R neurons in response to 10 µM glutamate (Fig. 7c). However, in the majority of Q336R neurons but never in the isogenic control cells, the initial transient response was followed by a progressive secondary increase of [Ca^2+^]_c_ (Fig. 7d, e). This characteristic response, referred to as DCD was evident only at 100 µM in control cells (Fig. 7a). Glutamate-induced DCD drives excitotoxic neuronal cell death through a cascade of pathways^63,64^. Disruption of mitochondrial Ca^2+^ homoeostasis is the key contributing factor during glutamate excitotoxicity^17^. Therefore, we first measured the expression of the regulatory components of the MCU complex using immunoblotting (Fig. 7f). As seen in the patient fibroblasts, protein expression of MICU1 and MICU3, a brain- specific Ca^2+^ sensing regulator, were significantly reduced in Q336R neurons along with EMRE, while the relative levels of MCU and NCLX remained unchanged (Fig. 7g). BN-PAGE analysis of mitochondria isolated from isogenic and Q336R neuronal cultures revealed the ~1.1 MDa assembly representing MCU complex containing gatekeeper subunits in isogenic mitochondria and a prominent constitutively active ~400 kDa MCU-EMRE complex devoid of gatekeeper proteins in Q336R mitochondria (Supplementary Fig. 8c–e) as previously described in cortical mitochondria of *Afg3l2* neuron-specific knockout mice with spastic ataxia-neuropathy^65^. In addition to defective neuronal MCU assembly, native complex I assembly was also reduced in Q336R mitochondria (Supplementary Fig. 8c). Analysis of mRNA expression showed no significant change among the components of the MCU complex (Supplementary Fig. 8f) suggesting a post-translational regulation of MICU1/MICU3 expression/assembly in Q336R neurons.

Consistent with these results, the measurement of [Ca^2+^]_m_ in isogenic and Q336R neurons loaded with mito-Fura-2 AM, showed a higher resting [Ca^2+^]_m_ in Q336R neurons (Supplementary Fig. 8g). Moreover, confocal images analysis of ratiometric images (F_355_/F_405_) revealed a swollen mitochondrial pool with a high F_355_/F_405_ ratio in Q336R neurons compared to isogenic control neurons which showed elongated mitochondrial morphology with a low F_355_/F_405_ ratio (Supplementary Fig. 8h). Exposure to a physiological concentration of glutamate evoked an early rise in [Ca^2+^]_m_ and a larger [Ca^2+^]_m_ peak in Q336R neurons compared to isogenic control neurons (Fig. 7h-l). Ratiometric image analysis also showed a rapid [Ca^2+^]_m_ rise in Q336R neurons in response to glutamate challenge (Supplementary Fig. 8i). Glutamate-induced [Ca^2+^]_m_ overload in Q336R neurons was confirmed using the low-Ca^2+^ affinity mt-AEQ probe (Supplementary Fig. 8j). These results are consistent with [Ca^2+^]_m_ overload as a cause of excitotoxic glutamate-induced DCD and neuronal cell death. Therefore, we analysed cytochrome c (Cyt c) release in neurons exposed to 10 µM glutamate overnight (12 h) by immunolabelling neurons for the TOM20 and Cyt c (Fig. 7m). The release of cytochrome c from mitochondria (TOM20^**+**^ and Cyt c^**-**^) is the major trigger for caspase activation during the initial events of apoptotic cell death cascade. After glutamate challenge, Q336R neurons showed a marked increase in TOM20 intensity without Cyt c intensity suggesting Cyt c release in comparison to isogenic control neurons (Fig. 7n). Notably, Q336R neurons also showed an aberrant remodelling of mitochondrial morphology accompanied by a significant increase in swollen mitochondria pool in the somas and a more fragmented form in the axons (Supplementary Fig. 8k). Altogether, these results show that impaired mitochondrial Ca^2+^ signalling and the ensuing [Ca^2+^]_m_ overload increases the vulnerability to glutamate-induced DCD and cell death in Q336R neurons.

### JP1-138 improves mitochondrial Ca^2+^ buffering capacity and bioenergetic function in Q336R neurons

**Fig. 8:**
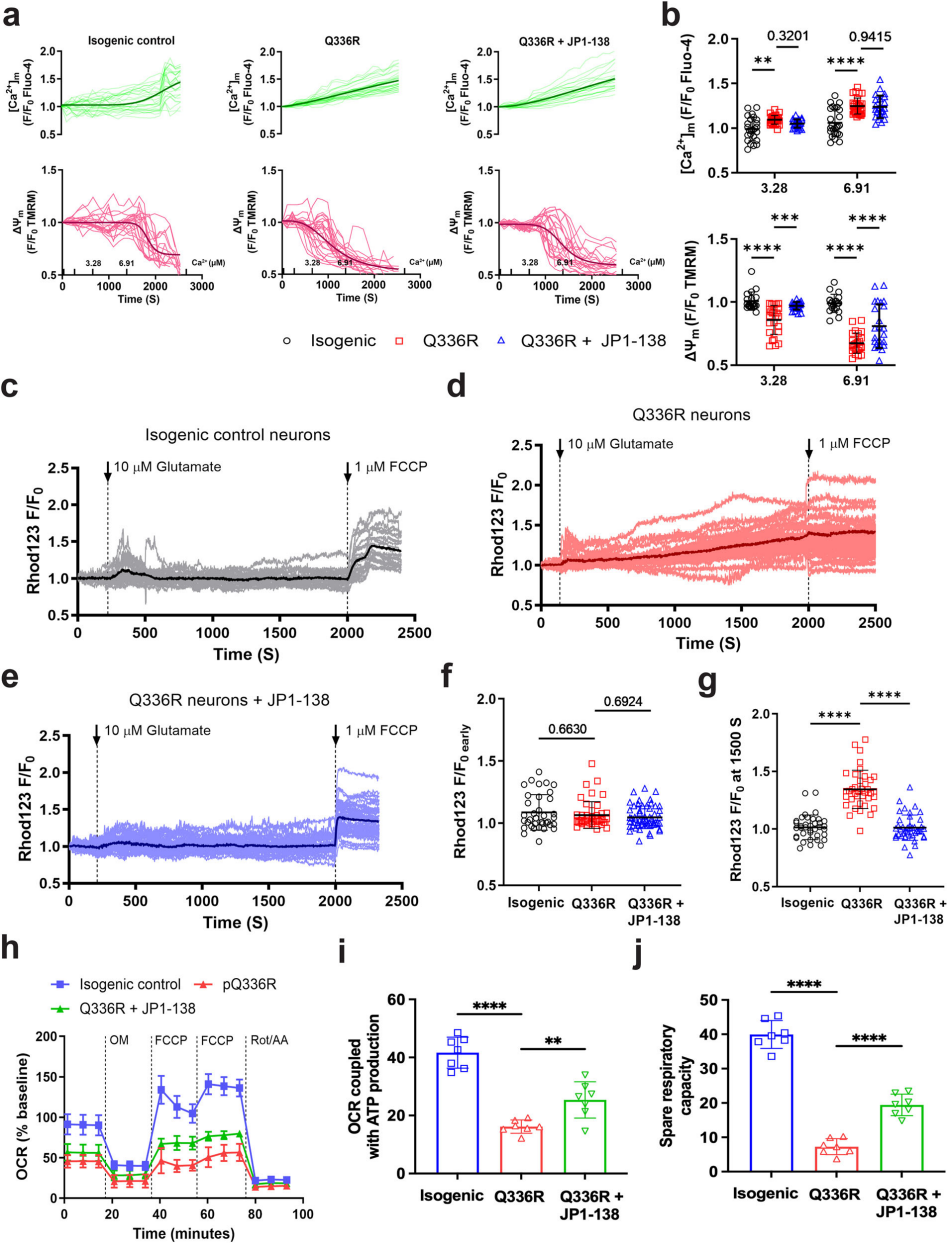
JP1-138 increases mitochondrial Ca^2+^ buffering capacity and partially rescues bioenergetic function in Q336R neurons. **a** Mean ± SD traces of Fluo-4 AM fluorescence (reporting [Ca^2+^]_m_) and TMRM fluorescence (reporting ΔΨ_m_) in digitonin-permeabilised isogenic and Q336R neurons bathed in pseudo-intracellular solution with or without JP1-138 (0.5 μM). Upward ticks indicate sequential additions of exogenous Ca^2+^, (*n* = 26 runs). **b** Peak Fluo-4 (upper) and TMRM (lower) responses to increasing Ca^2+^ concentrations in permeabilized isogenic and Q336R neurons with or without JP1-138, (*n* = 26, **p = 0.0050, ****p = 2.11 × 10^-7^, ***p = 0.0005, ****p = 9.2 × 10^-7^, 4.3 × 10^-5^, 9.2 × 10^-7^). **c–e** Mitochondrial membrane potential measured using Rhodamine-123 (dequench protocol) during exposure to 10 μM glutamate in isogenic and Q336R neurons treated with JP1-138 (0.5 μM) or DMSO. Increased Rhod123 fluorescence indicates mitochondrial depolarisation. Q336R neurons showed greater glutamate-induced depolarisation than isogenic neurons, which was rescued by JP1-138. FCCP-induced depolarisation represents total ΔΨ_m_ after glutamate, (*n* = 42 and 55 neurons for isogenic and Q336R respectively). **f**, **g** Quantification of mitochondrial depolarisation at an early time point and at 1500 s after 10 μM glutamate, expressed as Rhod123 F/F_0_, in isogenic, Q336R, and Q336R + JP1-138 neurons (*n* = 36, 45 and 57 neurons, respectively, ****p = 1 × 10⁻¹⁶, 1 × 10⁻¹⁶). **h** Normalised OCR traces from isogenic and Q336R neurons treated with JP1-138 (0.5 μM) or vehicle for 3 days (*n* = 7 runs). **i**, **j** ATP-linked respiration and spare respiratory capacity calculated from (**h**) (*n* = 7 wells, **p = 0.0070, ****p = 7.99 × 10^-15^, 7.99 × 10^-15^, 2.51 × 10^-5^). Data in (**b**, **f–j**) are mean ± SD with individual data points from independent experiments. Statistical analysis used one-way ANOVA with Tukey’s test or two-way ANOVA with Holm–Šidák test.

An increase in [Ca^2+^]_m_ is the most potent inducer of mPTP opening and therefore provides a direct link between sustained increases in [Ca^2+^]_m_ and mitochondrial dysfunction during excitotoxic injury in neurons^66^. Given that the pharmacological inhibition of mPTP in patient fibroblasts suppresses mtDNA-induced IFN response and partially restores mitochondrial bioenergetic function, we first determined mitochondrial Ca^2+^ retention capacity of neurons which were digitonin permeabilised after co-labelling with Fluo-4 AM (which is lost from the cytosol but retained in mitochondria after permeabilization^67^) and TMRM to monitor dynamic changes in [Ca^2+^]_m_ and ΔΨ_m,_ respectively, using sequential additions of exogenous CaCl_2_ as described in Supplementary Fig. 9a. The highest fold-change in Fluo-4 intensity was evident only after the fourth successive Ca^2+^ addition (6.91 µM final Ca^2+^) in permeabilised isogenic neurons, indicating the Ca^2+^ uptake threshold and cooperative activation of MCU by Ca^2+^ (Fig. 8a). Subsequent Ca^2+^ addition (13.3 µM final Ca^2+^) induced permeability transition culminating in the collapse of ΔΨ_m_ as indicated by the rapid loss of the TMRM signal. In contrast, mitochondria from Q336R neurons showed an almost linear increase in Fluo-4 intensity with successive Ca^2+^ additions indicating a lower threshold for activation and a constitutively active MCU complex. As a consequence of [Ca^2+^]_m_ overload, mPTP opening and rapid dissipation of ΔΨ_m_ was observed at lower concentrations of Ca^2+^ (between 1.32 and 3.28 µM) in the Q336R cells (Fig. 8b). Notably, the preincubation of Q336R mitochondria with JP1-138 significantly increased the threshold of mPTP opening and the loss of ΔΨ_m_ without affecting the rate of Ca^2+^ uptake indicating the protective effect of JP1-138 from [Ca^2+^]_m_ overload-induced mPTP opening and mitochondrial depolarisation (Fig. 8a, b). To demonstrate this under (patho)physiological conditions, isogenic and Q336R neurons were loaded with Rhodamine-123 in ‘dequench mode’ to monitor time-dependent changes in ΔΨ_m_ following exposure to glutamate. After an initial transient depolarisation indicated by an early rise in fluorescence in response to glutamate, ΔΨ_m_ recovered to the baseline in almost all the isogenic neurons (Fig. 8c, f). In contrast, Q336R neurons showed a delayed secondary depolarisation in the majority of the neurons (Fig. 8d), coincident with the glutamate-induced DCD response^66^. Moreover, an uncoupler-induced mitochondrial depolarisation caused a smaller additional depolarisation in comparison to isogenic neurons confirming near total dissipation of ΔΨ_m_ induced by glutamate in the Q336R neurons. Importantly, preincubation with JP1-138 completely protected the Q336R neurons from glutamate-induced collapse of ΔΨ_m_ (Fig. 8e–g). To determine the bioenergetic differences under unstimulated resting state, we measured basal and maximal respiration in isogenic and Q336R neurons. Both ATP-linked respiration rate and spare respiratory capacity were reduced in Q336R neurons (Fig. 8h). However, long-term treatment with JP1-138 resulted in a small but significant increase in both basal and maximal respiration rate in Q336R neurons (Fig. 8i, j). Together, these results demonstrate that JP1-138 effectively increases mitochondrial Ca^2+^ buffering capacity and protects Q336R neurons from glutamate-induced loss of ΔΨ_m_, and improves mitochondrial respiration, thus highlighting a potential therapeutic strategy to target neurodegeneration in *EPG5*-related disorders.

## Discussion

Impaired mitochondrial quality control resulting from depletion of core autophagic proteins plays a critical role in the regulation of glucose homoeostasis^68^, differentiation and function of primary immune cells^69^, and the control of sterile inflammation and tissue repair^70,71^. Whilst defective autophagy has been identified as the primary mechanism in *EPG5*-related disorders, downstream pathogenic effects remain poorly understood. Our recent work demonstrated that loss of EPG5 impaired mitochondrial homoeostasis due to defective PINK1/PARKIN-dependent mitophagy in patient-derived cells^7^. Here, we identify impaired mitochondrial Ca^2+^ signalling and the resulting bioenergetic insufficiency as major contributing factors to the neurodegenerative phenotype observed in patients with *EPG5*-RD. Our data show that dysregulation of mitochondrial Ca²⁺ signalling leads to distinct pathophysiological outcomes in EPG5-mutant primary fibroblasts and iPSC-derived cortical neurons. In cortical neurons, which are intrinsically vulnerable due to their high metabolic demand and physiological activity, mitochondrial Ca²⁺ overload increases susceptibility to excitotoxicity and cell death. In contrast, in proliferative primary fibroblasts, mitochondrial Ca²⁺ overload predominantly drives maladaptive inflammatory signalling through mtDNA release and activation of the cGAS–STING–type I IFN pathway. These data indicate that differences in mitochondrial bioenergetic reserve and tissue-specific demands may further contribute to the broader multisystem phenotype observed in patients carrying truncating versus missense EPG5 mutations.

Patient fibroblasts with either truncating or missense mutations showed an almost complete deficiency of normal EPG5 protein (~ 280 kDa) suggesting the involvement of both transcriptional and post-translational mechanisms in regulating mutant EPG5 protein expression. Previous reports demonstrated that both Q336R and R1631Q mutations create null alleles through defective mRNA splicing leading to the production of multiple aberrant splice isoforms that are degraded by nonsense-mediated mRNA decay^6,72^. At the protein level, EPG5 exhibits a high turnover rate and is predominantly regulated by lysosomal degradation^73^. Consequently, the limited availability of residual *EPG5* transcripts and/or rapid degradation of mutant EPG5 protein likely determines the overall abundance and stability of EPG5 in patient-derived fibroblasts carrying either missense or truncating variants. Consistent with this, impaired mitochondrial bioenergetic function and altered mitochondrial morphology were observed across all patient fibroblast lines. The mitochondrial respiratory defect was associated with downregulation of OXPHOS subunit expression despite an increase in mtDNA copy number. Gene enrichment analysis showed upregulation of *PPARGC1A* transcription factor encoding PGC1α, a master regulator of mitochondrial biogenesis (Supplementary Table 2), suggesting a compensatory response that may partially offset impaired mitophagy. Together, these findings indicate that defective mitophagy flux and mitochondrial dysfunction represent shared pathophysiological mechanisms across different classes of EPG5 mutations.

Mitophagy defects may also impact local mitochondrial quality control regulated by mitochondrial proteases involved in IMM proteome remodelling and activation of the mitochondrial unfolded protein response^74–76^. Unexpectedly, we found downregulation of MICU1 and MICU2 in patient fibroblasts and MICU1 and MICU3 in EPG5-mutant neurons as the causative mechanism underlying [Ca^2+^]_m_ overload and ensuing mPTP opening. Pathogenic mutations in MICU1 itself are associated with a rare childhood disorder that include neurodevelopmental, neurodegenerative and neuromuscular phenotypes with some close parallels to *EPG5*-RD^54,77^. Patients with loss-of-function MICU1 mutations present with a progressive extrapyramidal neurodegenerative disorder^54^, features that have a significant overlap with the clinical phenotype associated with *EPG5*-RD, including ataxia, dystonia, and tremor as well as basal ganglia abnormalities on magnetic resonance imaging. Similarly, microcephaly, myopathy, seizures and immunodeficiency are shared features of both MICU1 deficiency and EPG5 deficiency. Furthermore, [Ca^2+^]_m_ overload and mitochondrial dysfunction due to downregulation of NLCX and MCU remodelling has been implicated in the neuropathology and memory decline in Alzheimer’s disease^35^. Although the mechanism of MCU remodelling in patient fibroblasts and EPG5 mutant neurons defined by downregulation of MICUs and EMRE protein levels is not directly investigated here, it seems likely that stress-induced activity of quality control proteases may regulate the turnover of MCU regulatory subunits. Proteostasis stress arising from impaired mitophagy may activate OMA1-mediated DELE1 processing, triggering the integrated stress response (ISR)^75,76^ as evident by the ISR transcriptional signature observed in patient fibroblasts (Supplementary Table 2). In addition, alternative IMM or matrix proteases, such as YME1L1^78^ OMA1^79^ or AFG3L2^65^ may contribute to the degradation of MICUs and EMRE during ISR activation.

In EPG5-deficient patient fibroblasts, a prominent pathophysiological feature observed during histamine-induced mitochondrial Ca²⁺ overload was a limited, rather than widespread, opening of the mPTP. This restricted mPTP opening enabled the release of mtDNA into the cytosol, where it activated the cGAS–STING pathway and drove a type I IFN inflammatory response. Our data further suggest that this inflammatory programme may be sustained in a chronic, low-grade manner in patient fibroblasts, allowing continued cell survival and proliferation while avoiding acute cell death. A conceptually similar phenomenon, termed minority mitochondrial outer membrane permeabilization (miMOMP), has been described previously, whereby MOMP in a small subset of mitochondria permits mtDNA efflux through BAX/BAK macropores and activates cGAS–STING signalling, promoting age-associated inflammation in vivo^41^. However, while the formation of large VDAC or BAX/BAK pores in the outer mitochondrial membrane can provide an exit route for mitochondrial contents^40,80^, these mechanisms alone do not fully explain how the tightly packed inner mitochondrial membrane becomes permeabilized. Our findings in EPG5-deficient fibroblasts identify early upstream events underlying this process and demonstrate that unregulated mitochondrial Ca²⁺ uptake and overload compromise IMM integrity by sensitising mitochondria to mPTP opening, thereby enabling mtDNA release from a limited subset of damaged mitochondria, linking impaired mitochondrial Ca²⁺ homoeostasis to chronic innate immune activation in EPG5-deficient cells.

Consistent with this model, *Epg5*-deficient mice exhibit exaggerated pulmonary inflammation sufficient to inhibit influenza pathogenesis and increased resistance to intestinal viral infections, accompanied by spontaneous sterile inflammation^81,82^. Although enhanced IFN and TNF signalling were identified as key drivers of this hyperinflammatory phenotype, the upstream mechanisms linking autophagic dysfunction to innate immune activation remained unclear. Notably, these cytokine levels are also increased in patients with *EPG5*-RD^49^ and show a hyperimmunity against influenza. In addition, we also found upregulated expression of pro-survival NF-κB target genes, such as *CFLAR* and *BIRC2* (Supplementary Table 2) in patient RNA-seq datasets in addition to the type I/III IFN signature genes. Independent studies have shown that stress-induced mtDNA or mtRNA release to cytoplasm triggers IFN-I response that confers resistance to viral infection^83–85^. Our findings extend these observations by demonstrating that defective mitophagy and mtDNA release drive constitutive activation of the cGAS–STING pathway independently of infection, potentially preconditioning antiviral immunity while simultaneously promoting sterile inflammation. This may provide a mechanistic explanation for the lack of influenza infections observed in patients with *EPG5*-RD.

In iPSC-derived neurons carrying the pathogenic Q336R mutation, mitochondrial respiration and CI assembly were impaired, similar to patient fibroblasts. The exposure to glutamate at concentrations that were innocuous for isogenic neurons caused a rapid collapse of bioenergetic capacity, disruption of [Ca^2+^]_c_ homoeostasis and excitotoxic death in Q336R neurons. Glutamate-induced excitotoxicity in Q336R neurons is attributed to a lower mitochondrial Ca^2+^ uptake threshold and [Ca^2+^]_m_ overload which combined with increased ROS production leads to increased susceptibility to mPTP opening caused by downregulation of MICU1/3 holocomplex and a constitutively active MCU complex. Thus, an increased bioenergetic demand during neuronal metabolic workload may amplify the compromised mitochondrial function due to [Ca^2+^]_m_ overload and trigger a rapid pathological cascade culminating in cell death. Our data demonstrate that an impaired mitochondrial metabolism and Ca^2+^ signalling sensitises neurons to excitotoxic injury which may play an important role in neurodegeneration in *EPG5*-RD and may also contribute to the increased seizure susceptibility observed in around two-thirds of patients^48^. Loss of AFG3L2 activity, a mitochondrial m-AAA protease associated with spinocerebellar ataxia and loss of Purkinje neuron, results in impaired processing of EMRE and assembly of MCU complex enabling [Ca^2+^]_m_ overload, mPTP opening and neuronal death in mice^65^. Moreover, neuronal loss of MICU1 increased [Ca^2+^]_m_ overload-induced excitotoxicity and caused progressive degeneration of motor neurons in *MICU1*-KO mice^33^. These findings further highlight the impaired mitochondrial Ca^2+^ homoeostasis as a key driver of excitotoxic neuronal cell death in early-onset neurodegenerative diseases.

Pharmacological prevention of [Ca^2+^]_m_ overload-induced mPTP opening using JP1-138 increased mitochondrial Ca^2+^ buffering capacity and partially rescued mitochondrial bioenergetic function in both patient fibroblasts and Q336R neurons. Prolonged treatment with JP1-138 also suppressed mtDNA release and downstream activation of the cGAS-STING activation and IFN response more effectively than CsA, the prototypical inhibitor of mPTP with off-target effects^32^. Nevertheless, the incomplete recovery of mitochondrial bioenergetics underscores that impaired mitophagy, while clearly pathogenic, is unlikely to fully account for the complex cellular dysfunction observed in EPG5 deficiency. Further studies exploring pharmacological modulation of mitophagy will be required to define the extent to which mitochondrial quality control alone can restore metabolic homoeostasis and suppress inflammation in EPG5-deficient cells. Importantly, given the marked phenotypic heterogeneity observed in *EPG5*-RD, broader autophagy-dependent mechanisms are likely to contribute to disease pathogenesis.

The classic Vici syndrome phenotype manifests as a severe infantile-onset neurodevelopmental disorder with multisystem involvement, characterised by agenesis of the corpus callosum, cataracts, hypopigmentation, cardiomyopathy, combined immunodeficiency, microcephaly, and failure to thrive^3^. Some of these features, particularly neurodevelopmental abnormalities, microcephaly and callosal agenesis overlap with phenotypes attributed to stalled autophagosomal biogenesis and/or clearance and represent established clinical and radiological hallmarks of disorders affecting core autophagy pathway^7^. However, other hallmark features of Vici syndrome, including hypopigmentation and combined immunodeficiency with memory B‑cell depletion, are less readily explained by impaired mitophagy alone and instead point to defects in endolysosomal trafficking^86^, melanocytic function^87^, intracellular transport, and xenophagy^88^. As EPG5 functions as a tethering factor between autophagosomes and lysosomes, EPG5 deficiency likely represents a convergence of mitochondrial dysfunction with more widespread autophagy‑dependent defects. Indeed, loss of EPG5 has been shown to disrupt additional selective autophagy pathways, including nucleophagy^89^, Toll-like receptor signalling^49^, and endocytic trafficking^90^, suggesting that the distinctive EPG5 phenotype arises from the combined impact of impaired mitophagy and broader disruptions of cellular homoeostasis.

Several studies have demonstrated that genetic ablation or pharmacological inhibition of STING confers protection against inflammation-mediated neuronal loss in mouse models of Parkinson’s disease, amyotrophic lateral sclerosis, and lysosomal storage disorders^46,91,92^. In our study, although pharmacological inhibition of STING using H-151 effectively suppressed STING-dependent type I IFN signalling in EPG5-deficient fibroblasts, it failed to rescue mitochondrial dysfunction suggesting that activation of the cGAS–STING pathway occurs downstream of mitochondrial dysfunction rather than acting as a driver of the mitochondrial bioenergetic defect. Submicromolar concentrations of JP1-138 were highly effective at maintaining ΔΨ_m_ even after [Ca^2+^]_m_ overload in both patient fibroblasts and Q336R neurons. Our group recently reported improved mitochondrial activity and preclinical safety of JP1-138 compound with 20-fold greater brain concentration following a single dose in mice^55^, highlighting its therapeutic potential in targeting Ca^2+^ mishandling and excitotoxicity in *EPG5*-RD and other neurodegenerative diseases with similar pathogenic mechanisms.

In summary, this study advances our understanding of how impaired mitochondrial Ca^2+^ signalling and dysfunction contributes to the presentation and progression of *EPG5*-RD by identifying a cascade of events starting from bioenergetic deficiency and unregulated mitochondrial Ca²⁺ uptake, leading to Ca²⁺ overload, sensitisation to mPTP opening, and ultimately divergent downstream consequences in different cell types. In patient-derived fibroblasts, these events culminate in low-grade, mtDNA-driven chronic inflammatory signalling, whereas in neurons they increase vulnerability to excitotoxicity and cell death. Importantly, these findings suggest that mitochondrial dysfunction resulting from impaired quality control in EPG5 deficiency may exerts tissue-specific effects. In the central nervous system, this may manifest as both cell-autonomous neuronal vulnerability and non–cell-autonomous inflammatory responses in metabolically competent cell populations, such as glia, which together may contribute to and accelerate neurodegeneration. This progressive chain of events may account for the clinically variable and multisystem pathophysiology of *EPG5*-RD but also help to identify multiple sites for pharmacological intervention as potential novel therapeutic targets. These findings will together serve to improve our understanding of the progressive neurodegeneration in patients with *EPG5*-related disorders and other neurodegenerative disorders where similar defects in autophagy are an important pathogenic mechanism.

## Methods

### Ethics statement

Primary fibroblasts carrying specific EPG5 mutations (Supplementary Table 1) were isolated from skin biopsies of male and female patients previously obtained as part of the routine diagnostic process^7^. All patients and/or their legal guardians gave informed consent to anonymized publication. Age and sex matched healthy control fibroblasts were obtained from the MRC Centre for Neuromuscular Disorders Biobank, London (13DN28).

### Cell culture and treatments

All cell lines were cultured and maintained in Dulbecco’s modified Eagle’s medium (10566016, Gibco) supplemented with 10% foetal bovine serum (16140071, Gibco), and 1% Antibiotic-Antimycotic (15240096, Gibco) and incubated at 37 °C with 5% CO_2_. Fibroblasts lines were maintained at maintained at sub-confluence (80%) and cultured between 3 and 10 passages. All cell cultures were routinely checked for mycoplasma using MycoAlert™ Mycoplasma Detection Kit (LT07-118, Lonza). For transfection, Human Dermal Fibroblasts Nucleofector Kit (VPI-1002, Lonza) was used according to the manufacturer’s instructions. Fibroblasts were treated with either freshly prepared histamine (CAY33828, Cambridge Bioscience), Ru360 (557440, Calbiochem) or a thapsigargin (586005, Merck), H-151 (S6652, Selleck) and G140 (S9945, Selleck) stock solutions made in DMSO, at indicated working concentrations. Fibroblasts and neuronal cultures were preincubated with JP1-138 in imaging media for 10 min before live cell imaging. For long-term treatments with either JP1-138, CsA (1101, Tocris) or H-151, the culture medium was changed every two days.

### SDS-PAGE and immunoblotting

Fibroblast cultures from 60 mm dishes were washed with PBS and collected by trypsinization (0.5% trypsin-EDTA; 15400054, Gibco). Similarly, mixed neuronal cultures were gently lifted and collected using Accutase. To prepare the samples for Western blot analysis, cell pellets were homogenised in 100–150 μl RIPA buffer (R0278, Sigma-Aldrich) containing 1x Halt Protease and Phosphatase Inhibitor Cocktail (78440, Thermo Scientific). Following homogenisation, cell lysates were centrifuged at 16,000 g at 4 °C for 30 min and the protein concentration in the supernatant was quantified using the Pierce BCA Assay Kit (23227, Thermo Scientific). Equivalent amounts of total protein (30 µg) samples in NuPAGE 4x LDS Sample Buffer (NP0007, Invitrogen) and 2% β-mercaptoethanol (63689, Sigma-Aldrich) were boiled at 95 °C for 10 min. Proteins were separated on either 12% Bolt Bis-Tris Plus (NW04127, Invitrogen) or 4–12% NuPAGE Bis-Tris polyacrylamide gels (NP0335, Invitrogen) immersed in MOPS running buffer (NP0001, Invitrogen) and transferred onto PVDF membranes (1620175, Bio-Rad). Membranes were then blocked in SuperBlock Blocking Buffer (37545, Thermo Scientific) for 1 h at room temperature (RT) and probed overnight at 4 °C using indicated primary antibodies. After incubation with appropriate secondary antibodies, protein bands were detected using a chemiluminescent reagent (Luminata Forte Western HRP substrate; WBLUF0100, Merck) and imaged using a ChemiDoc system (Bio-Rad). Membranes were further stripped using Restore Western Blot Stripping Buffer (21059, Thermo Scientific) and re-probed with additional primary antibodies. Quantification of the protein bands was performed using with ImageJ (NIH) and Image Lab software, v6.0.1 (Bio-Rad). Details of all the antibodies used in this study can be found in Supplementary Table 3.

### Mitochondrial oxygen consumption rate (OCR)

Measurements of mitochondrial respiration in fibroblasts and neurons were conducted with the Seahorse Bioscience XFe96 bioanalyzer using the Seahorse XF Cell Mito Stress Test Kit (103015-100, Agilent). Fibroblasts were seeded at a density of 1 × 10^4^ cells/well on XF96 cell culture microplates (102416-100, Agilent) and cultured for 1-2 days and/or treated for the indicated time. For neuronal cultures, iPSC-derived NPCs were seeded at a density of 5 × 10^4^ cells/well on XF96 cell culture microplates coated with poly-L-lysine and laminin for terminal neuronal differentiation and maturation using the method describe above. On the day of the experiment, the culture medium was replaced with Seahorse XF Base medium (103334-100, Agilent) supplemented with 1 mM pyruvate (11360070, Gibco), 2 mM glutamine (25030081, Gibco) and 10 mM glucose (A2494001, Gibco) and incubated in a CO_2_-free incubator for 30 min at 37 °C before loading into the Seahorse Analyser. After the measurement of basal respiration, the drugs oligomycin (5 μM), FCCP (1 μM, 2 μM), and rotenone/antimycin A (0.5 μM/ 0.5 μM) were added to each well in sequential order. Data was analysed using the XF Cell Mito Stress Test Report Generator. The OCR data from fibroblasts were normalised to the cell number obtained after counting the numbers of cell nuclei with ImageXpress MicroXL as described above. The OCR data from neurons were also normalised to the cell counts obtained after constructing a calibration curve and calculating the normalised cell number per well with CyQuant Direct Cell Proliferation Assay Kit (C35011, Invitrogen) and expressed as a percentage of the baseline measurement of untreated line.

### Mitochondrial membrane potential (ΔΨ_m_) in fibroblasts and neurons

The steady-state and time-lapse measurement of ΔΨ_m_ in fibroblast was carried out using tetramethylrhodamine methyl ester (TMRM) in the redistribution mode where a lower fluorescence intensity indicates reduced ΔΨ_m_ and vice versa. For the steady-state measurement of ΔΨ_m_, cells were seeded at a density of 1 × 10^5^ cells/dish on fluorodishes (FD35-100, WPI) and cultured for 1 day and/or treated for the indicated time. On the day of the experiment, cells were washed once with recording buffer 1 (RB1; 150 mM NaCl, 4.25 mM KCl, 4 mM NaHCO_3_, 1.25 mM NaH_2_PO_4_, 2 mM CaCl_2_, 1.2 mM MgCl_2_, 10 mM D-glucose, and 10 mM HEPES at pH 7.4) and incubated with 25 nM TMRM (T668, Invitrogen) and 1 μM Calcien-AM (C3100MP, Invitrogen) in RB1 for 30 minutes at 37 °C. Following incubation, cells were washed twice with RB1 and TMRM was added in the RB1 to avoid its depletion while imaging. z-stacks with 0.45 µm thickness with a pixel dwell time of 1.54 μs were acquired using a Zen Black software-controlled LSM 880 confocal microscope (Carl Zeiss) equipped with a plan-Apochromat 63x/1.4 oil DIC objective lens and an Ar (λ_ex_ = 488 nm, λ_em_ = 500–550 nm for Calcien-AM)and DPSS laser source (λ_ex_ = 561 nm, λ_em_ = 575–625 nm for TMRM) at 37 °C. Mean TMRM fluorescence intensity was quantified using the same threshold across all the samples and the percentage mitochondrial mass was calculated from the area of the binarized images of Calcien-AM and TMRM using ImageJ.

For the time-lapse measurement of ΔΨ_m_, images were acquired from a single z-plane every 2 s interval with a pixel dwell time of 1.54 μs. After acquiring baseline TMRM images, 10 uM histamine was applied directly into the fluorodishes using a micropipette and subsequent images were obtained to monitor the effect of histamine on ΔΨ_m_. At the end of each experiment, 2.5 μM Carbonyl cyanide 4-(trifluoromethoxy) phenylhydrazone (FCCP; C2920, Sigma-Aldrich) was added as a positive control which depolarized the mitochondria. Time series were analysed using ImageJ by selecting and measuring the mean fluorescence intensity in the regions of interests (ROIs) in each field. Individual ΔΨ_m_ traces were normalised to their baseline intensity obtained before stimulation.

Measurement of ΔΨ_m_ in neurons was carried out using Rhodamine-123 in the dequench mode where dequenching or increase in Rhodamine-123 fluorescence indicates mitochondrial depolarisation and loss of ΔΨ_m_. Neuronal cultures seeded in a glass-bottom 96-well plate (655892, SensoPlate) were labelled with 10 μg/ml Rhodamine-123 (R8004, Sigma-Aldrich) in BrainPhys Imaging Optimised Medium (05796, STEMCELL Technologies) at 37 °C and 5% CO_2_ for 20 min. After incubation, cells were washed thrice and imaged as above using a Plan-Neofluar 40x/1.30 oil objective and an Ar laser source (λ_ex_ = 488 nm, λ_em_ = 510–600 nm). For all experiments, the laser illumination intensity was kept to a minimum (max 0.5%) to avoid phototoxicity and photobleaching. After acquiring baseline Rhodamine-123 images, neurons were stimulated using 10 μM glutamate (G1626, Sigma-Aldrich) and subsequent images were acquired. At the end of each experiment, 1 μM FCCP was added to evaluate the Rhodamine-123 fluorescence intensity corresponding to the loss of ΔΨ_m_. Time series were analysed using ImageJ as described above.

### Measurement of mitochondrial NAD(P)H

NAD(P)H autofluorescence imaging was carried out to investigate the mitochondrial redox state in fibroblasts. Cells were seeded at a density of 1 × 10^5^ cells/dish on fluorodishes and cultured for 1 day and/or treated for the indicated time. On the day of the experiment, cells were washed once with RB1 and imaged using the Carl Zeiss LSM 880 confocal microscope equipped with an ultraviolet (UV) laser (λ_ex_ = 355 nm, λ_em_ = 410–480 nm) and quartz Plan-Apochromat 63x /1.4 oil objective at 37 °C. Images were acquired from a single z-plane with the pinhole wide open to maximise signal and laser illumination intensity kept to a minimum (0.1-0.2%) to avoid phototoxicity. After acquiring baseline images, cells were first exposed to 2.5 μM FCCP to depolarise the mitochondria completely and achieve maximal respiration. The oxidation of the mitochondrial pool of NADH into non-fluorescent NAD^+^ led to the lowest fluorescence signal, which was considered as 0%. Thereafter, 1 mM cyanide (NaCN), an inhibitor of mitochondrial respiration, was added to allow the regeneration of the mitochondrial pool of NADH (the highest fluorescence signal was considered 100%). The baseline autofluorescence acquired from each cell was normalised between the minimal (0%) and maximal (100%) fluorescent signals to calculate the redox index.

### Simultaneous measurement of [Ca^2+^]_c_ and [Ca^2+^]_m_ in fibroblasts

Dynamic measurements of cytosolic and mitochondrial Ca^2+^ concentrations were carried out in fibroblasts loaded with 2.5 μM Fluo-4 AM and 1 μM mito-Fura-2 AM in RB1 containing 0.005% Pluronic F-127 for 30 minutes at 37 °C. Following incubation, cells were washed thrice with RB1 and imaged using the Carl Zeiss LSM 880 confocal microscope equipped with an UV (λ_ex_ Ca^2+^ bound mito-Fura-2 = 355 nm, λ_ex_ Ca^2+^ unbound mito-Fura-2 = 405 nm, λ_em_ = 470–600 nm) and Ar laser (λ_ex_ = 488 nm, λ_em_ = 505–560 nm for Fluo-4) and quartz Plan-Apochromat 63x /1.4 oil objective at 37 °C. Images were acquired sequentially from a single z-plane every 12.5 s interval with a pixel dwell time of 1.54 μs. For all experiments, the laser illumination intensity was kept to a minimum (0.25–0.5%) to avoid phototoxicity. After acquiring baseline images for about 100 s, 10 μM histamine was applied directly into the fluorodishes using a micropipette and subsequent images were obtained. A total of 1 μM ionomycin was added at the end of each course as a positive control. Time series were analysed in ImageJ as described above. After background subtraction, the change in Fluo-4 intensity was calculated relative to the baseline and shown as ΔF/F_0_. For mito-Fura-2, ratios between the fluorescence intensity excited at 405 nm and at 355 nm were calculated at each time point and plotted as 355/405 ratio representing [Ca^2+^]_m_. The values for area under the curve (AUC), basal mito-Fura-2 ratio, t_0.5_ to [Ca^2+^]_m_ peak and t_0.5_ of stabilisation for [Ca^2+^]_c_ peak were calculated for each independent experiment in GraphPad Prism.

### Cytosolic and mitochondrial calcium imaging in neurons

Measurements of cytosolic Ca^2+^ concentrations were performed in neuronal cultures seeded on fluorodishes and loaded with 2.5 μM FuraFF AM (CAY20416, Cambridge Bioscience) in BrainPhys Imaging Optimised Medium containing 0.001% Pluronic F-127 for 30 min at 37 °C. Following incubation, cells were washed thrice and imaged on a custom-made Olympus IX71 inverted epifluorescence microscope equipped with a UAPO/340 20x/0.70 water objective and a Xenon arc lamp. FuraFF was excited alternately at 340 nm ± 20 nm and 380 nm ± 20 nm, and emitted light was collected through a dichroic T510lpxru (Chroma). Images were acquired with a Zyla CMOS camera (Andor) every 2 s using MetaFluor 7.8.12.0 (Molecular Devices). After acquiring baseline images for about 100 s, neurons were stimulated with 10 μM and/or 100 μM glutamate applied directly into the fluorodishes using a micropipette. A total of 2 μM ionomycin was added at the end of each experiment as positive control. For analysis, time series were imported and processed in ImageJ as described above. Ratios between the fluorescence intensity excited at 380 nm and at 340 nm were calculated at each time point and plotted as 340/380 ratio representing [Ca^2+^]_c_. FuraFF ratiometric images at the indicated time points were acquired using MetaMorph 7.8.12.0 (Molecular Devices). A linear blue-to-red pseudocolour lookup table (LUT) was applied to the ratio images, covering the full range of the data. For FuraFF experiments, LUT values were linearly mapped from 0 to 2.5.

Mitochondrial Ca^2+^ concentrations were measured in neurons loaded with 1 μM mito-Fura-2 AM in BrainPhys imaging medium containing 0.001% Pluronic F-127 for 30 min at 37 °C. Following incubation, cells were washed thrice and imaged using the Carl Zeiss LSM 880 confocal microscope equipped with an UV laser and quartz Plan-Apochromat 63x/1.4 oil objective, as described above. Images were acquired from a single z-plane every 5 s interval with a pixel dwell time of 2.05 μs. After acquiring baseline images, 10 μM glutamate was applied directly into the fluorodishes using a micropipette and subsequent images were obtained. Time series were analysed in ImageJ and the ratios between the fluorescence intensity excited at 405 nm and at 355 nm were calculated at each time point and plotted as 355/405 ratio representing [Ca^2+^]_m_. Basal mito-Fura-2 ratio, t_0.5_ to [Ca^2+^]_m_ peak and peak amplitude values were calculated for each cell in GraphPad Prism. Representative mito-Fura-2 ratiometric images were generated using Metamorph. For mito-Fura-2 experiments, a linear blue-to-red pseudocolour lookup table (LUT) was applied, covering the full range of ratio values (0 to 0.85) in each dataset.

### Mitochondrial calcium retention assay in permeabilized neurons

To measure the capacity of neuronal mitochondria to accumulate Ca^2+^ until the mPTP opens, digitonin-permeabilized neurons were loaded with Fluo-4 AM and TMRM to simultaneously measure [Ca^2+^]_m_ uptake and the loss of ΔΨ_m_ as a readout for mPTP opening. Briefly, neuronal cultures seeded on fluorodishes, were washed once with RB1 and loaded with 5 μM Fluo-4 AM, 25 nM TMRM and 0.1% Pluronic F-127 dissolved in RB1 for 30 min at room temperature (RT). Following incubation, cells were permeabilized with 10 μg/ml digitonin in RB3 (5 mM NaCl, 130 mM KCl, 1 mM KH_2_PO_4_, 20 mM HEPES, 6.5 mM MgCl_2_, 1.5 mM EGTA, 6 mM EDTA, 0.4 mM CaCl_2_, 2 mM malate, 2 mM succinate, 2 mM glutamate and 2.5 mM ADP; pH adjusted to 7.3 with 1 M KOH) containing 250 nM TMRM and 2 μM thapsigargin for 10 minutes. After permeabilization, excess digitonin was washed off and the neurons were imaged in RB3 with 250 nM TMRM and 2 μM thapsigargin to inhibit sarco/endoplasmic reticulum Ca^2+^ ATPase. Imaging was performed as described above, after baseline TMRM and Fluo-4 images, small volumes (5 or 10 μl) of 50 mM CaCl_2_ were carefully added to the chamber using a P10 micropipette until the loss of TMRM signal. Images were acquired every 120 seconds over a total time of 30 minutes. The final free Ca^2+^ ion concentration in RB3 was calculated using WEBMAXC Extended software: https://somapp.ucdmc.ucdavis.edu/pharmacology/bers/maxchelator/webmaxc/webmaxcE.htm. TMRM and Fluo-4 fluorescence intensities were calculated relative to baseline and shown as ΔF/F_0_ where ΔF is the difference in fluorescence between baseline and post CaCl_2_ addition and F_0_ is the basal fluorescence. Ratios of Fluo-4 and TMRM fluorescence intensities (ΔF/F_0_) were plotted against time and fitted with a nonlinear sigmoidal curve function.

### Live cell imaging of mtDNA release in fibroblasts

Quantitative analysis of mtDNA dynamics in fibroblasts was carried by co-labelling cells seeded in fluorodishes with 25 nM TMRM, 1 μM PicoGreen (P11495, Invitrogen), 1 μM mito-Fura-2 and 0.005% Pluronic F-127 in RB1. After incubation for 30 min at 37 °C, cells were washed thrice and imaged on Carl Zeiss LSM 880 confocal microscope equipped with an UV (λ_ex_ Ca^2+^ bound mito-Fura-2 = 355 nm, λ_ex_ Ca^2+^ unbound mito-Fura-2 = 405 nm, λ_em_ = 470–600 nm), Ar (λ_ex_ = 488 nm, λ_em_ = 505–560 nm for PicoGreen) and DPSS laser source (λ_ex_ = 561 nm, λ_em_ = 570–700 nm for TMRM) and quartz Plan-Apochromat 63×/1.4 oil objective. Images were acquired from a single z-plane every 12.5 s interval with a pixel dwell time of 0.85 μs. After acquiring baseline images for about 250 s, 10 μM histamine was applied directly into the fluorodishes using a micropipette and subsequent images were obtained. At the end of each experiment, 1 μM FCCP was added at the end of the time course to completely depolarise mitochondria. Time series were processed and analysed in ImageJ and Metamorph. TMRM fluorescence intensities were normalised relative to baseline and shown as ΔF/F_0_, mito-Fura-2 fluorescence intensities were plotted as 355/405 ratio representing [Ca^2+^]_m_ as described above. Ratio images were displayed using a linear blue-to-red pseudocolour lookup table (LUT) spanning the full data range, with values mapped from 0 to 0.85. For the quantification of cytosolic PicoGreen puncta, number of PicoGreen puncta outside the nucleus and the TMRM-labelled mitochondrial perimeter were counted manually between the time points after the challenge with 10 μM histamine and before the application of 1 μM FCCP.

Fibroblasts expressing mTagBFP-cGAS (Addgene:102603, a gift from Nicolas Manel) were co-labelled with 1 μM PicoGreen and 25 nM TMRM and imaged as described above with mTagBFP-cGAS visualised using an UV laser source (λ_ex_ = 405 nm, λ_em_ = 410–470 nm). For the quantification of the cytosolic PicoGreen puncta with cGAS, cells displaying puncta positive for cGAS and PicoGreen were selected and a binary image of TMRM and PicoGreen channels was generated in ImageJ. A segmented mask of non-mitochondrial Picogreen puncta was generated using image calculator and number of events with cGAS and PicoGreen overlap within each ROI were scored.

### Immunocytochemistry

#### Immunofluorescence analysis of fibroblasts

Immunofluorescence analysis of cytosolic mtDNA puncta/ mtDNA release in fibroblasts was performed using Airyscan imaging. Briefly, cells were seeded at a density of 2 × 10^4^ cells/well in a glass-bottom 96-well plate and cultured for 1-2 days. On the day of the experiment, cells were fixed in 5% PFA in PBS for 30 min at RT, then washed three times with PBS, followed by quenching with 50 mM ammonium chloride in PBS. After fixation, cells were washed thrice with PBS and permeabilized in 0.1% Triton X-100 in PBS for 10 min, followed by three washes in PBS. Permeabilized cells were then blocked with 10% FBS in PBS, followed by incubation with primary antibodies in 5% FBS in PBS, for 2 h at RT. Cells were then washed three times with 5% FBS in PBS and labelled with the corresponding secondary antibodies prepared in 5% FBS in PBS for 1 h at RT. After three washes, super-resolution Airyscan images were acquired on a Zeiss LSM 880 with Airyscan detector in SR mode using all 32 pinholes. At least 8 z-stacks with optimal slice sizes 0.45 µm (overview image) and 0.185 µm (inset image) with a pixel dwell time of 4.10 μs were acquired. Prior to image analysis, raw confocal micrographs were automatically processed into deconvoluted Airyscan images using the Zen Black software. The number of DNA puncta outside the nucleus and mitochondrial perimeter were counted in ImageJ by generating a nuclear and mitochondrial mask and subtracting this segmented mask from DNA channel in image calculator. The colocalization coefficient (Pearson’s R-value) was quantified using Coloc 2 plugin. For 3D rendering and quantification of cytosolic mtDNA puncta or mtDNA release events, cells with enlarged nucleoids were selected and a segmented mask of IMM was created using surface function in Imaris 9.8 (Bitplane). Using this mask mtDNA nucleoids outside the surface were differentiated using spot function. A surface rendering of OMM was generated similarly and the transparency of IMM and OMM was adjusted to allow the visualisation of mtDNA nucleoid spots. Details of all the antibodies used in this study can be found in Supplementary Table S3.

For the cGAS immunofluorescence, cells were cultured, fixed and permeabilized as above. After permeabilization, cells were blocked with 5% BSA in PBS for 1 h at the RT and incubated overnight with primary antibody diluted in PBS with 1% BSA and 0.1% Triton X-100. After three washes in PBS, cells were incubated with secondary antibody diluted in PBS with 1% BSA for 2 h at RT. Cells were then washed three times and incubated with DAPI for 5 min to label nuclei. After three washes, cells were imaged using the confocal microscope as described above. The nuclear cGAS intensity in each cell was quantified from the DAPI positive area and the cytosolic cGAS intensity per cell was calculated from subtracting the DAPI positive area from total cGAS mask area in ImageJ.

#### Immunofluorescence analysis of neurons

Neuronal cultures were treated overnight with 10 μM glutamate and fixed with 4% PFA in 2x microtubule stabilisation buffer (160 mM PIPES, 5 mM EGTA, 1 mM MgCl_2_, pH 7.2) for 10 min at RT. After fixation, cells were gently washed three times with PBS and 0.1% Triton X-100 for 10 min. Following permeabilization, cells were blocked with buffer containing 3% goat serum and 0.1% Triton X-100 in PBS for 30 min and incubated with primary antibodies prepared in blocking buffer for 2 h at RT. After three washes, cells were incubated with the corresponding secondary antibodies prepared in the blocking buffer for 1 h at RT. After three washes, nuclei were stained using DAPI and imaged as described above. z-stacks were imported into ImageJ and somas and axons were manually segmented using β-tubulin III staining. A colocalization mask of TOM20 and Cyt c channel was subtracted from the Cyt c channel allowing the separation of pixel intensities occurring outside the mitochondria. These binary images were used to quantify the area occupied by mitochondria without Cyt c.

### Statistical analysis

No formal statistical methods were used to predetermine sample sizes. Quantitative data were obtained from at least three independent biological replicates and are represented as mean ± SD, unless otherwise specified. Confocal images shown are representative of at least three independent biological replicates. Where applicable, curve fitting was performed using linear or nonlinear regression functions. Normality was assessed using the Shapiro–Wilk test. For normally distributed data, comparison between two groups were performed using unpaired, two-tailed Welch’s *t*-test. Comparisons involving grouped or repeated-measures data were analysed using two-way mixed-effects models, or repeated- measures ANOVA, whereas other multiple-group comparisons were analysed using one-way ANOVA, as appropriate. Post hoc multiple-comparisons tests, including Tukey’s, Šidák’s, or Holm–Šidák’s tests, were applied as indicated in the figure legends. Statistical differences were considered significant when the α-value of *p* was <0.05. Exact *p* values are provided in the figure legends, significance is otherwise denoted as *p < 0.05, **p < 0.01, ***p < 0.001, ****p < 0.0001. Analyses were not performed blinded. Raw data were stored in Microsoft Excel, and statistical analyses were performed using GraphPad Prism 10.4.1.

### Reporting summary

Further information on research design is available in the Nature Portfolio Reporting Summary linked to this article.

## Supplementary information

Supplementary Information

Description of Additional Supplementary File

Supplementary Movie 1

Supplementary Movie 2

Supplementary Movie 3

Reporting Summary

Transparent Peer Review file

## Source data

Source Data

## Data Availability

The RNA-seq datasets generated and analysed during the current study are available in the GEO repository GSE316460. Gene-set Analyses using biological processes and the KEGG pathway analysis of the RNA-sequencing experiment and the unprocessed blot images are available in the Source Data file of this paper. Source data are provided with this paper.
