# Supplementary material for: Pathogenic variants in the autophagy-tethering factor EPG5 drive neurodegeneration through mitochondrial dysfunction and innate immune activation
Source: Nat Commun. 2026 May 26;17:6887. doi: 10.1038/s41467-026-73538-7 (PMC13388713; doi:10.1038/s41467-026-73538-7)
Supplement: Supplementary file 1 — Supplementary Information [file 41467_2026_73538_MOESM1_ESM.pdf]

SUPPLEMENTARY INFORMATION

Pathogenic variants in the autophagy-tethering factor EPG5 drive neurodegeneration through mitochondrial dysfunction and innate immune activation

Kritarth Singh[1*], Hormos Salimi Dafsari[2,3,4*], Olivia Gillham[1], Haoyu Chi[1], Ivet Mandzhukova[1], Ioanna Kourouzidou[1], Preethi Sheshadri[1], Chih-Yao Chung[1], Valeria Pingitore[5,6], Fleur Vansenne[7], David L. Selwood[6], Diana Pendin[8,9], Gyorgy Szabadkai[1,9], Manolis Fanto[10†], Heinz Jungbluth[4,11†], Michael R. Duchen[1†]

[1]Department of Cell and Developmental Biology and Consortium for Mitochondrial Research, University College London, Gower Street, London WC1E6BT, UK

[2]Department of Pediatrics, Faculty of Medicine and University Hospital Cologne, University of Cologne

[3]Max-Planck-Institute for Biology of Aging and Cologne Excellence Cluster for Ageing-associated Diseases, Cologne, Germany

[4]Department of Paediatric Neurology, Evelina London Children's Hospital, Guy's & St Thomas' NHS Foundation Trust, London, UK

[5]Department of Health and Biomedical Sciences, Universidad Loyola Andalucía, Seville, Spain

[6]Drug Discovery, UCL Wolfson Institute for Biomedical Research, University College London, London WC1E6BT, UK

[7]Department of Genetics, University Medical Center Groningen, University of Groningen, Groningen, Netherlands

[8]Neuroscience Institute, National Research Council, Padua 35131 Italy

[9]Department of Biomedical Sciences, University of Padua, Padua 35131 Italy

[10]Division of Basic and Clinical Neuroscience, IoPPN, King's College London

[11]Randall Centre for Cell and Molecular Biophysics, Muscle Signalling Section, Faculty of Life Sciences and Medicine (FoLSM), King's College London, London, UK

*These authors contributed equally: KS and HSD

†These authors jointly supervised this work: MD, HJ and MF

Correspondence to: Michael R. Duchen, email: m.duchen@ucl.ac.uk

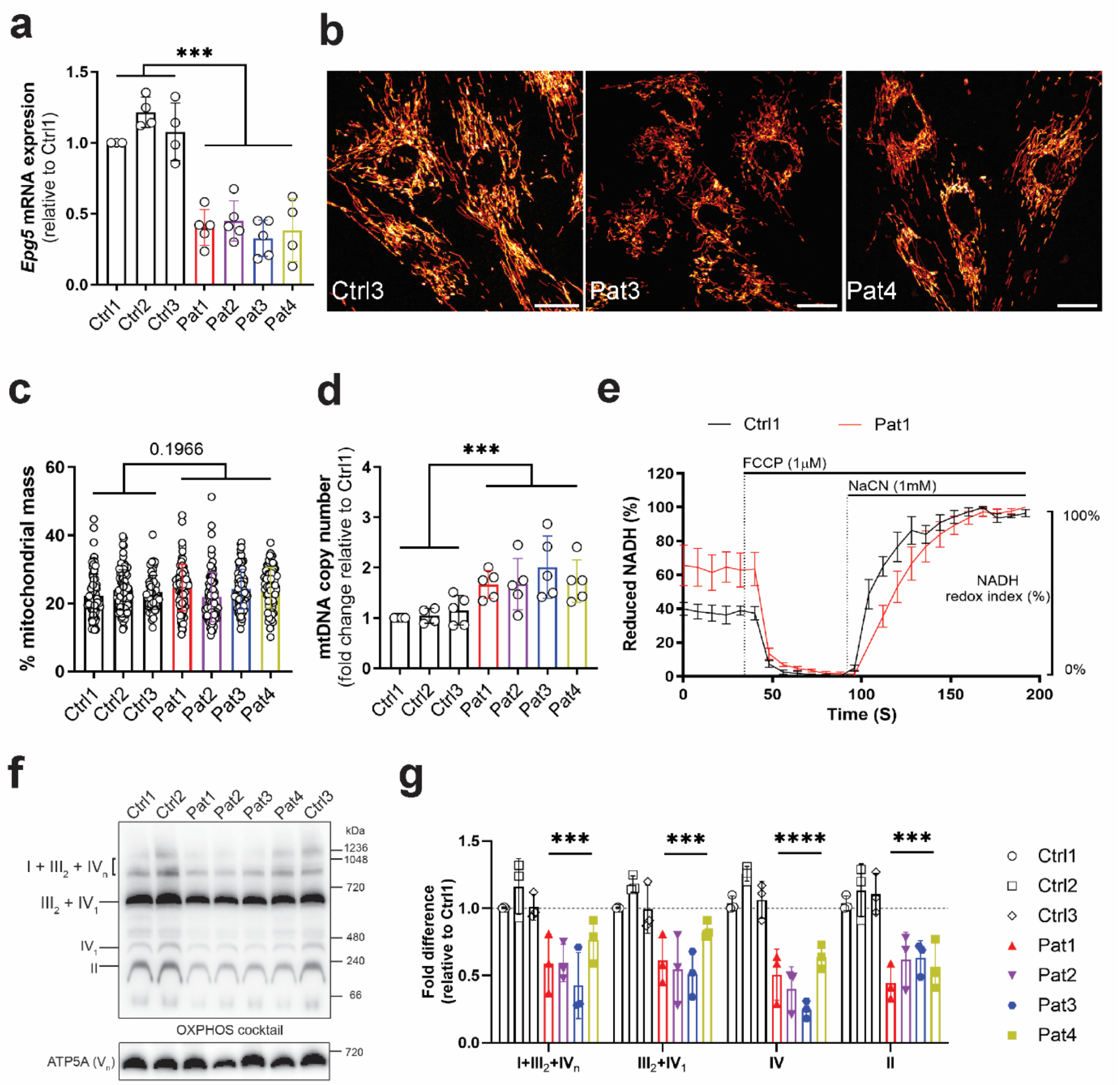

Supplementary Figure 1. Mitochondrial dysfunction and increased mtDNA copy number in EPG5-deficient fibroblasts. a qRT-PCR analysis of *EPG5* mRNA expression levels in patient fibroblasts normalized to the levels Ctrl1, (n= 4, ****p*= 0.0007). **b** Representative confocal image of TMRM-labelled cells showing steady-state mitochondrial membrane potential ($\Delta\Psi_m$) in Ctrl3, Pat3 and Pat4 fibroblasts. Scale bars: 20 µm. **c** Quantification of mitochondrial volume occupancy calculated relative to total cytosolic volume measured by Calcein AM fluorescence and expressed as percentage mitochondrial mass, (n= 90, 95, 76, 87, 84, 78 and 81 cells for Ctrl1, Ctrl2, Ctrl3, Pat1, Pat2, Pat3 and Pat4 respectively). **d** Quantification of mtDNA copy number measured by qPCR using an mtDNA-specific primer pair, from total genomic DNA isolated from control and patient fibroblasts, (n= 5, ****p*= 0.0008). **e** Representative traces of quantified NADH autofluorescence (mean ± SD) in Ctrl1 and Pat1 fibroblasts. After baseline acquisition, 2.5 µM FCCP was added to maximise

respiration and fully oxidise mitochondrial NADH, producing the lowest fluorescence signal, defined as the minimum (0% NADH). Subsequent addition of 1 mM NaCN blocked respiration and prevented NADH oxidation, allowing regeneration of the NADH pool and producing the highest fluorescence signal, defined as the maximum (100% NADH). The NADH redox index was calculated from these traces, (n= 5 runs). **f** Immunoblot analysis of respiratory chain protein expression and supercomplex assembly in mitochondria isolated from control and patient fibroblasts using blue native gel electrophoresis (BNGE). Supercomplex V_n was detected with ATP5A and used as a loading control. **g** Quantification of protein expression levels normalised to ATP5A and plotted as fold change relative to Ctrl1, (n= 3, ****p*= 0.0005, 0.006 and 0.0001, *****p*= 1.91×10^{-6})**.** Data in **a, c, d, e** and **g** are shown as mean ± SD with individual data points from independent experiments. Statistical analysis was performed using one- or two-way ANOVA with Šidák or Holm–Šidák multiple-comparisons tests, non-significant *p* values are given numerically.

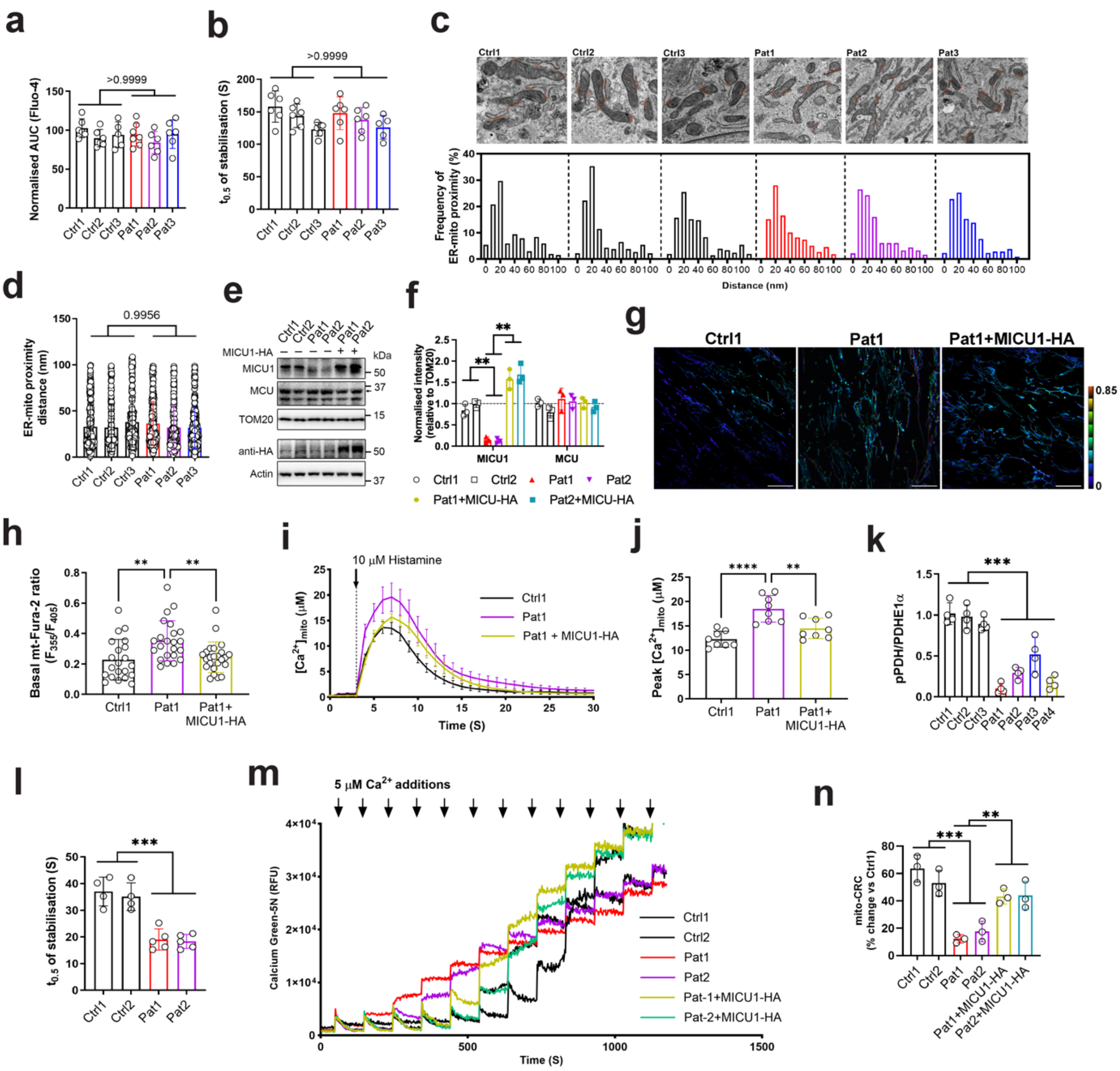

Supplementary Figure 2: MICU1 overexpression protects EPG5-deficient fibroblasts from mitochondrial Ca^{2+} overload and mPTP opening while ER–mitochondrial Ca^{2+} signaling remains unchanged. **a** Quantification of normalized areas under the curve (AUC) of Fluo-4 AM traces in response to 10 µM histamine, representing total ER Ca^{2+} released over time, calculated from the mean traces in Fig. 2c (n= 6). **b** Time required for 50% of the released ER Ca^{2+} to be cleared from the cytosol, calculated from the mean traces in Fig. 2c. **c** Representative TEM images of control and patient fibroblasts. Red segmented regions between the ER and mitochondria indicate ER–mitochondria contact sites. Histograms show the distribution of ER-mitochondria contact widths measured from the segmented regions. Scale bars: 0.5 µm. (n= 202, 184, 204, 218, 180, and 210 mitochondria analyzed for Ctrl1, Ctrl2, Ctrl3, Pat1, Pat2, Pat3 and Pat4 respectively, from three independent fixations). **d** Quantification of ER-mito contact widths from the dataset in c. **e** Immunoblot analysis of MICU1 and MCU in whole-cell lysates from control and patient fibroblasts with or without MICU1-HA

transfection. TOM20 and Actin were used as loading controls. **f** MICU1 and MCU protein levels relative to TOM20, normalized to Ctrl1, (n= 3, ***p*= 0.006556, 0.004639). **g** Representative mito-Fura-2 AM ratio images (F_{355}/F_{405}) of Ctrl1, Pat1 and MICU1-HA-transfected patient 1 fibroblasts. Scale bars, 20 µm. **h** Quantification of the mean mito-Fura-2 ratio per cell, showing resting mitochondrial Ca^{2+} concentration ($[Ca^{2+}]_m$), (n= 22, 23 and 24 cells for Ctrl1, Pat1 Pat1+ MICU1-HA respectively, ***p* =0.0033, 0.0092). **i** Mean traces of mitochondrial Ca^{2+} uptake measured using mtAEQ in response to 10 µM histamine (n= 8 runs). **j** Maximum $[Ca^{2+}]_m$ induced by 10 µM histamine in Ctrl1, Pat1 and Pat1 + MICU1-HA fibroblasts, (n= 8 ***p*= 0.0040, *****p*= 3.62×10^{-5}). **k** Ratio of phosphorylated PDH (PDH-E1α pS293) to total PDH (PDH-E1α), normalized to the average Ctrl1 ratio shown in Fig. 2j, (n= 4, ****p*= 0.0004). **l** Time to half-maximal $[Ca^{2+}]_m$ uptake after the first 5 µM $CaCl_2$ bolus, calculated from the inset in Fig. 2k (n= 4, ****p*= 0.0001). **m** Mitochondrial Ca^{2+} retention capacity measured in isolated mitochondria from control and patient fibroblasts with or without MICU1-HA transfection. Mean traces show extramitochondrial Ca^{2+} measured using Calcium Green-5N following repeated additions of 5 µM $CaCl_2$. **n** Quantification of mitochondrial Ca^{2+} retention capacity, expressed as percentage inhibition relative to Ctrl1 mitochondria, (n= 3, ***p*= 0.0017, ****p*= 0.0001). Quantitative data in **a, b, d, f, h-l** and **n** are shown as mean ± SD with individual data points from independent experiments. Statistical analysis was performed using one-way ANOVA with Tukey's test or one-/two-way ANOVA with Šidák or Holm–Šidák multiple-comparisons tests, non-significant *p* values are given numerically.

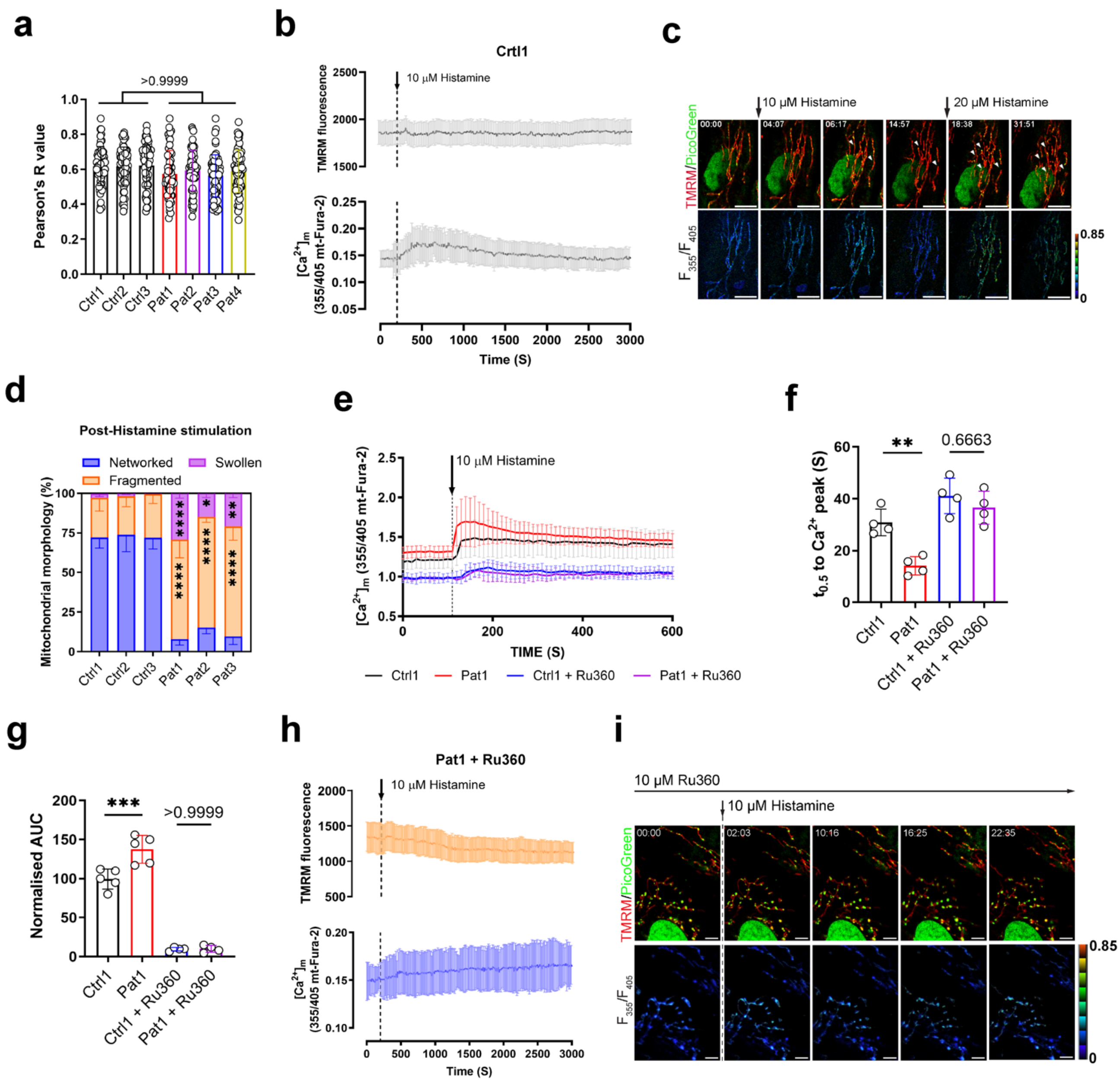

Supplementary Figure 3. Histamine stimulation does not induce mitochondrial depolarization in control fibroblasts, and Ru360 prevents mtDNA release under mitochondrial Ca^{2+} overload in patient fibroblasts. a Pearson's R values for co-localisation of TOM20 (red) and citrate synthase (blue) in mitochondria from the confocal images shown in Fig. 3a, (n= 61, 66, 63, 55, 64, 60 and 62 cells for Ctrl1, Ctrl2, Ctrl3, Pat1, Pat2, Pat3 and Pat4 respectively). **b** Mean traces of TMRM fluorescence and mitochondrial Ca^{2+} concentration ($[Ca^{2+}]_m$) following 10 μM histamine challenge in Ctrl1 fibroblasts co-labelled with TMRM, mito-Fura-2 AM, and PicoGreen, (n= 9 runs). **c** Time-lapse confocal images of Ctrl1 fibroblasts co-labelled with TMRM (red) and PicoGreen (green) in the upper panels, and mito-Fura-2 AM ratio images (F_{355}/F_{405}) in the lower panels, corresponding to the quantification in b. Elapsed time after 10 μM histamine challenge is indicated (Supplementary Movie 1). White arrowheads indicate mitochondrial fragmentation events following 10 μM and 20 μM histamine challenge. Scale bars, 5 μm. **d** Quantitative morphometric analysis of TMRM-labelled

mitochondria in control and patient cells after stimulation with 10 μM histamine. Mitochondria were classified as networked, fragmented, or swollen and plotted as a percentage of the total mitochondrial population, (n= 3, **p= 0.001064, ***p= 0.000810, 0.000231, 0.000466, ****p= 0.000004, 0.000074). **e** Mean $[Ca^{2+}]_m$ traces measured in mito-Fura-2 AM-labelled control 1 and patient 1 fibroblasts, either untreated or pretreated with 10 μM Ru360 for 30 min, followed by stimulation with 10 μM histamine, (n= 5 runs). **f** Rate of mitochondrial Ca^{2+} uptake, quantified as the time taken to reach 50% of peak $[Ca^{2+}]_m$, calculated from the traces in e (n= 4, **p= 0.0058). **g** Quantification of normalized areas under the curve (AUC) of the mito-Fura-2 traces, representing total mitochondrial Ca^{2+} uptake over time, calculated from e (n= 5, ***p= 0.0009). **h** Mean traces of TMRM fluorescence and $[Ca^{2+}]_m$ change after 10 μM histamine challenge in Pat1 fibroblast pretreated with 10 μM Ru360 for 30 min and co-labelled with TMRM, mito-Fura-2 AM and PicoGreen, (n= 6 runs). **i** Time-lapse confocal images of patient fibroblasts pretreated with Ru360 and co-labelled with TMRM (red) and PicoGreen (green) in the upper panels, and mito-Fura-2 AM ratio images (F_{355}/F_{405}) in the lower panels, corresponding to h. Elapsed time after 10 μM histamine challenge is indicated (Supplementary Movie 1). Data in **a, b, d-h** are shown as mean ± SD with individual data points from independent experiments. Statistical analysis was performed using one-way ANOVA with Tukey's test or one-/two-way ANOVA with Šidák or Holm-Šidák test, non-significant p values are given numerically.

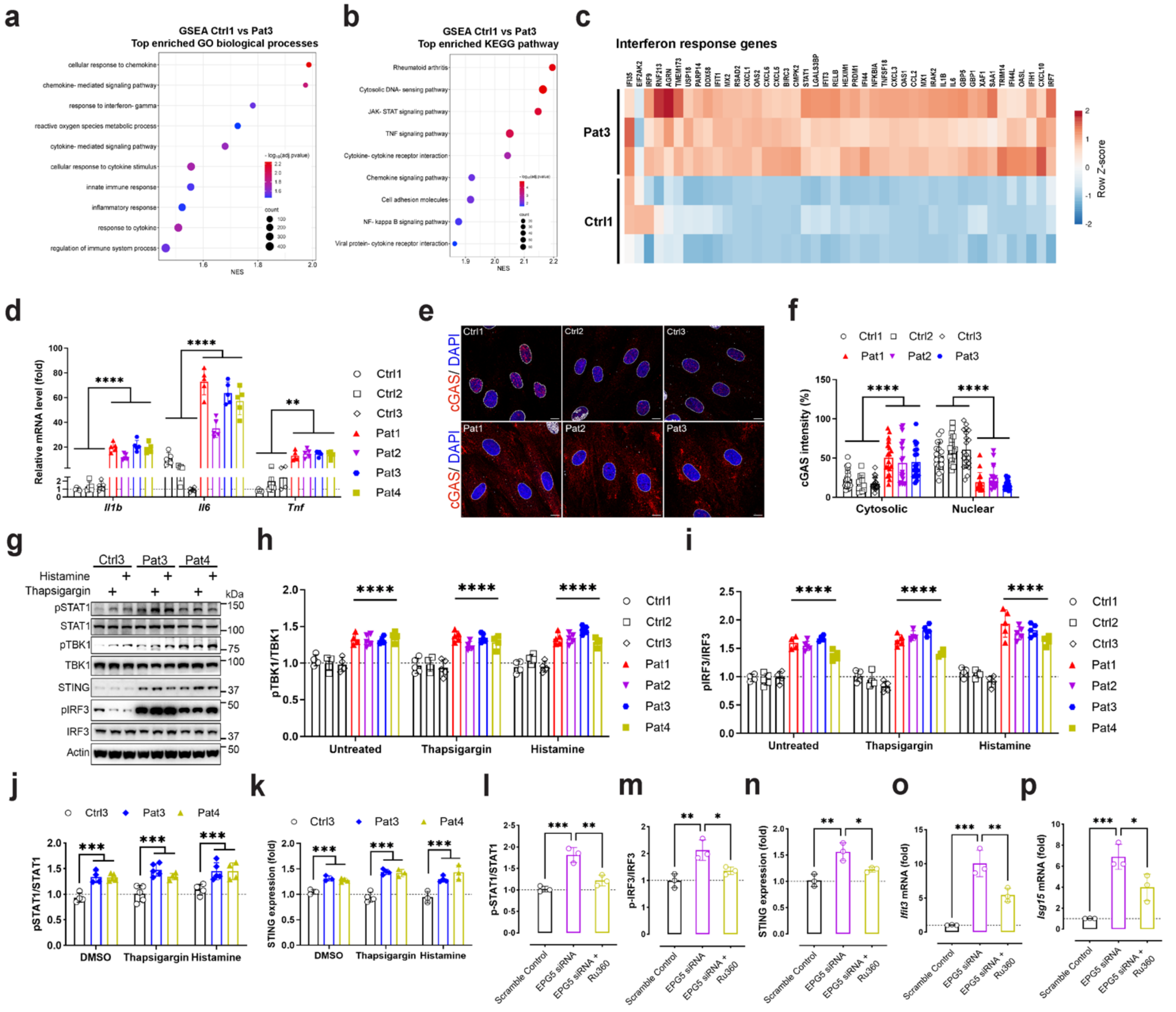

Supplementary Figure 4. Additional characterization of cGAS-STING activation and ISGs induction in patient-derived fibroblasts. a, b Pathway analysis of RNA-seq data from Pat3. Dot plots and gene set enrichment analysis (GSEA) were performed using Gene Ontology (GO) biological process and KEGG pathway gene sets. Adjusted p values were corrected for multiple testing using the Benjamini–Hochberg method (FDR control), NES, normalized enrichment score. **c** Heatmap of RNA-seq data displaying the top 50 upregulated differentially expressed type I/III interferon-related genes in Ctrl1 and Pat3 fibroblasts, (n= 3). **d** qRT-PCR analysis of pro-inflammatory interferon-stimulated genes (ISG) expression in control and patient fibroblasts, (n= 5, ****p*= 0.0029, ******p*= 1.37 × 10^{-5}, 2.07 × 10^{-5}). **e** Representative confocal images of control and patient fibroblasts immunolabelled with cGAS (red) antibody and counterstained with DAP1 to assess cytoplasmic and nuclear localization of cGAS. **f** Quantification of relative cGAS fluorescence intensity in the cytoplasmic and nuclear compartments, used to determine cytosolic cGAS translocation in control and patient fibroblasts, (n= 20,16, 21, 19, 19, 19 and 21 cells for Ctrl1, Ctrl2, Ctrl3, Pat1, Pat2 and Pat3 respectively, ******p*= 0.000011, 0.000003). **g** Immunoblot analysis of proteins involved in STING-

dependent type I interferon signalling in whole-cell lysates from control and patient fibroblasts treated with 10 μM histamine or 1 μM thapsigargin for 24 h. Actin was used as a loading control. **h** Ratio of phosphorylated TBK1 (pTBK1, Ser172) to total TBK1, normalized to Ctrl1 ratio, (n= 5, ****p= 1.07 $\times 10^{-7}$, 3.11 $\times 10^{-7}$, 1.49 $\times 10^{-8}$). **i, j** Ratio of pIRF3 (pSer396) and total IRF3 and pSTAT1 (p Tyr701) and total STAT1 band intensities normalized to the Ctrl1 ratio, (n= 5, ****p= 9.32 $\times 10^{-8}$, 1.22 $\times$ 10^{-11}, 1.26 $\times 10^{-8}$, ***p= 0.0002, 0.0010, 0.0005,). **k** STING protein expression normalized and plotted as fold change relative to control, (n= 4, ***p= 0.0003, 0.0006, 0.0002). **l, m, n** Ratios of pSTAT1 (Tyr701) to total STAT1, pIRF3 (Ser396) to total IRF3, and STING protein expression, each normalized to the scrambled negative control ratio, (n= 3, ***p= 0.0007, **p= 0.0033, **p= 0.0058, *p= 0.0347, **p= 0.0037, *p= 0.0338). **o, p** qRT-PCR analysis of ISG expression in fibroblasts transfected with control siRNA or *EPG5* siRNA with or without Ru360 pretreatment, (n= 3, *p= 0.0275, **p= 0.0097, ***p= 0.0003, 0.0009). Data in **d, f, h-p** are shown as mean ± SD and individual data points from independent experiments. Statistical analysis was performed using one-way ANOVA with Tukey's test or two-way ANOVA with Holm-Šidák test.

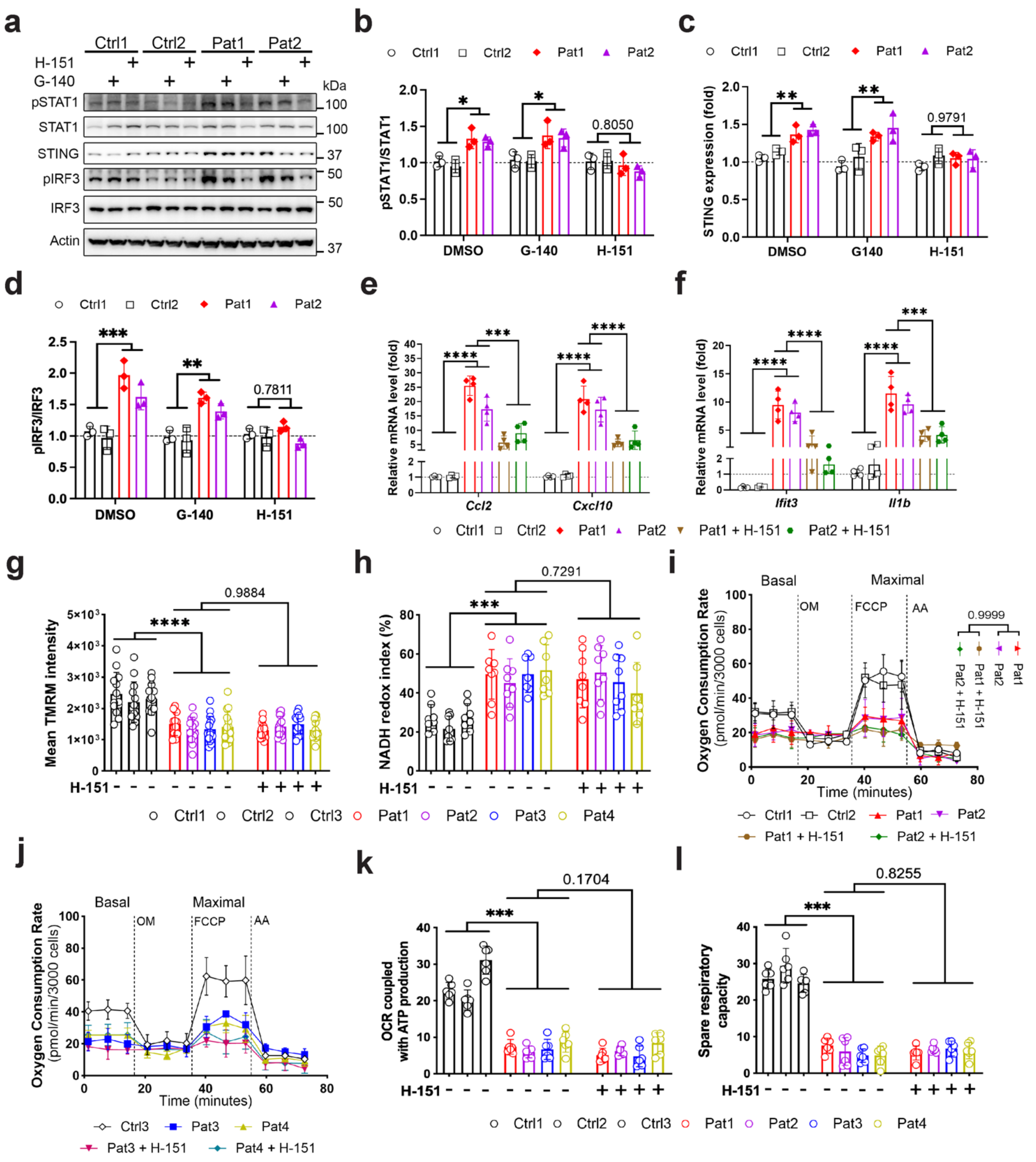

Supplementary Figure 5. Inhibition of STING activation by H-151 attenuates the STING-dependent interferon response but does not improve mitochondrial function in patient-derived fibroblast. a Immunoblot analysis of cGAS-STING signaling proteins in whole-cell lysates from control and patient fibroblasts treated for 24 h with either the STING inhibitor H-151 (1 µM) or the cGAS inhibitor G-140 (100 nM) for 24 h. Actin was used as a loading control. **b, c** Ratio of phosphorylated STAT1 (pTyr701) to total STAT1, normalized to Ctrl1 ratio, (n= 3, *p= 0.0123, 0.0171) and STING protein expression normalized and plotted as fold change relative to control, (n= 3, **p= 0.0055, 0.0071). **d** Ratio of phosphorylated IRF3 (pSer396) to total IRF3 normalized to the

Ctrl1 ratio, (n= 3, **p= 0.0031, ***p= 2.17 × 10^{-4}). **e, f** qRT-PCR analysis of ISG expression in control and patient fibroblasts either untreated or treated with H-151 (1 μM) for 24 h (n= 4, ***p= 0.0006, ****p= 5.47 × 10^{-9}, 4.70 × 10^{-8}, 2.33 × 10^{-6}, ***p= 0.0003, ****p= 2.99 × 10^{-9}, 1.16 × 10^{-6}, 1.18 × 10^{-5}). **g** Quantification of steady-state $\Delta\Psi_m$ measured by TMRM fluorescence intensity in control and patient fibroblasts, either untreated or treated with STING inhibitor, H-151 (1 μM, 24 h), (n= 14 cells for Ctrl1, Ctrl2, Ctrl3 and 16 cells for Pat1, Pat2, Pat3 and Pat4, ****p =1.73 × 10^{-5}). **h** NADH redox index in control and patient fibroblasts, either untreated or treated with H-151, (n= 8 cells for each control and patient, ***p= 0.0001). **i, j** Normalized OCR traces from controls and patient fibroblasts, either untreated or treated with H-151 for 3 days (0.5 μM), (n= 6 runs). **k, l** Normalized ATP-linked respiration and spare respiratory capacity in control and patient fibroblasts, calculated from the OCR traces, (n= 6 wells, ***p= 1.73 × 10^{-4}, 2.79 × 10^{-4}). Data in **b-h** and **j- l** are shown as mean ± SD with individual data points from independent experiments. Statistical analysis was performed using one- or two-way ANOVA with Šidák or Holm-Šidák test, non-significant p values are given numerically.

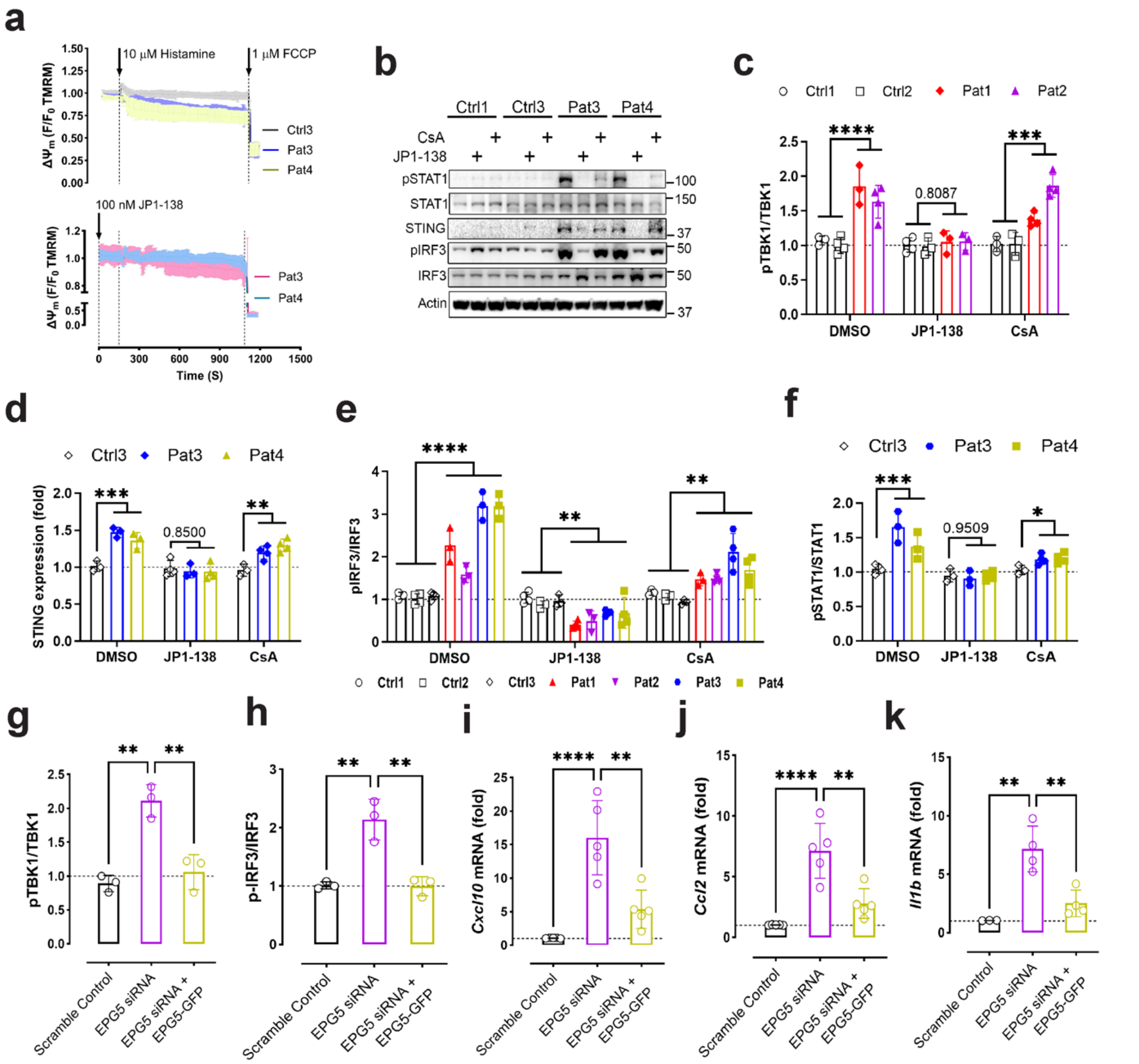

Supplementary Figure 6: Additional characterization of the effect of JP1-138 treatment on the STING-dependent interferon response in patient-derived fibroblasts. **a** Mean ± SD traces showing changes in mitochondrial membrane potential ($\Delta\Psi_m$) in response to 10 µM histamine and FCCP-induced depolarization in control and patient fibroblasts, in the absence (upper) and presence (lower) of JP1-138 (100 nM), (n= 20 runs). **b** Immunoblot analysis of proteins involved in the cGAS-STING pathway in whole-cell lysates from control and patient fibroblasts treated for 3 days with either CsA (1 µM) or JP1-138 (100 nM). Actin was used as loading a control. **c** Ratio of phosphorylated TBK1 (pSer172) to total TBK1, normalized to the Ctrl1 ratio shown in Fig. 5g, (n= 4, ****p*= 0.0001, *****p*= 0.000020). **d-f** STING protein expression, ratio of phosphorylated IRF3 (pSer396) to total IRF3 and ratio of phosphorylated STAT1 (p Tyr701) to total STAT, normalized and plotted as fold change relative to control, (n= 3, ****p*= 0.0004, ***p*= 0.0074, *****p*= 2.68×10^{-8}, ***p*= 0.0064, 0.0031, ****p*= 0.0003, **p*= 0.0162). **g, h** Ratio of phosphorylated TBK1 (pSer172) to

total TBK1, pIRF3 (pSer396) to total IRF3, normalized to the control siRNA ratio, (n= 3, ***p*= 0.0011, 0.0023, 0.0021, 0.0020). **i-k** qRT-PCR analysis of interferon-stimulated gene (ISG) expression in fibroblasts transfected with control siRNA or *EPG5* siRNA, with and without EPG5-GFP nucleofection, (n= 5, *****p*= 7.81×10^{-5}, ***p*= 0.0015, *****p*= 7.13×10^{-5}, ***p*= 0.0014, ***p*= 0.0011, 0.0038,). Data in **a** and **c-**k are shown as mean ± SD with individual data points from independent experiments. Statistical analysis was performed using one-way ANOVA with Tukey's test or two-way ANOVA with Holm-Šidák test, non-significant *p* values are given numerically.

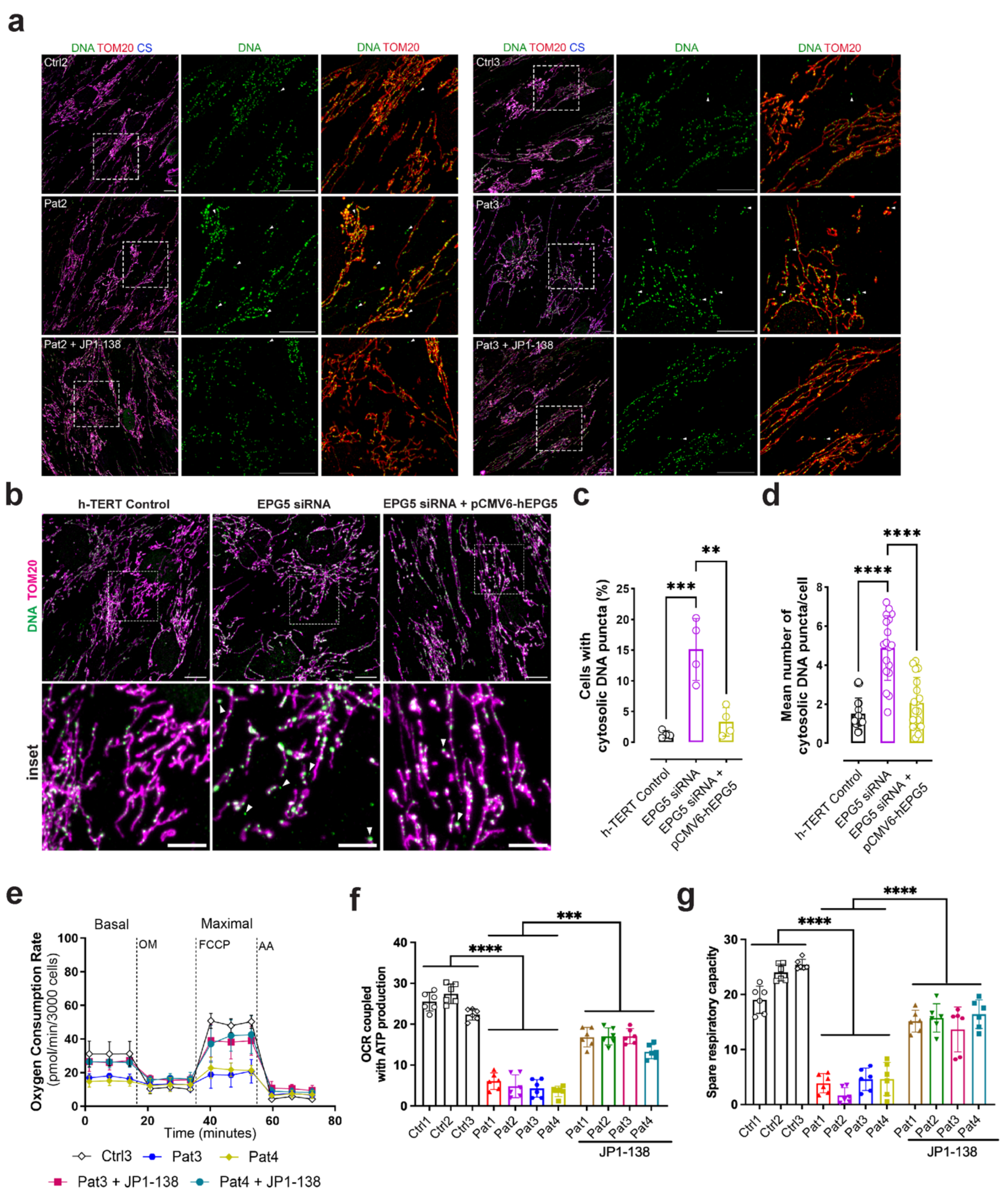

Supplementary Figure 7: Additional characterization of the effect of JP1-138 treatment on mitochondrial respiration and cytosolic mtDNA release in patient-derived fibroblast. a Representative Airyscan images of control and patient fibroblasts treated with JP1-138 (100 nM) or DMSO for 3 days and immunolabelled for DNA (green), TOM20 (red) and citrate synthase (blue). Insets highlight regions in patient cells where DNA does not co-localize with TOM20, indicated by white arrowheads, this is reduced following JP1-138 treatment. Scale bars: 10 µm (overview), 5 µm

and 2.5 µm (insets). **b** Representative Airyscan images of hTERT-immortalised control fibroblasts, *EPG5*-siRNA fibroblasts, and *EPG5*-siRNA fibroblasts expressing human EPG5, immunolabelled for DNA (green) and TOM20 (magenta). Insets below highlight regions where DNA does not co-localize with TOM20, indicated by white arrowheads. Scale bars: 10 µm (overview), 5 µm (insets). **c** Percentage of control siRNA, *EPG5*-siRNA and *EPG5*-siRNA+pCMV6-hEPG5 fibroblasts containing cytosolic DNA puncta, (n= 4, ***p*= 0.0015, ****p*= 0.0005). **d** Quantification of the number of cytosolic DNA puncta released per cell (n= 4, *****p*= 2.907×10^{-10}, 5.359×10^{-8}). **e** Normalized OCR traces from control and patient fibroblasts treated with JP1-138 (100 nM) or DMSO for 3 days (n= 6 runs). **f, g** Normalized ATP-linked respiration and spare respiratory capacity of control and patient fibroblasts calculated from the OCR traces in e and Fig. 6h (n= 6 wells, ****p*= 0.0005, *****p*= 2.05×10^{-7}, 1.43×10^{-5}, 3.48×10^{-8}, 7.26×10^{-5}). Data **c- g** are shown as mean ± SD with individual data points from independent experiments. Statistical analysis was performed using one-way ANOVA with Tukey's test or two-way ANOVA with Holm-Šidák test.

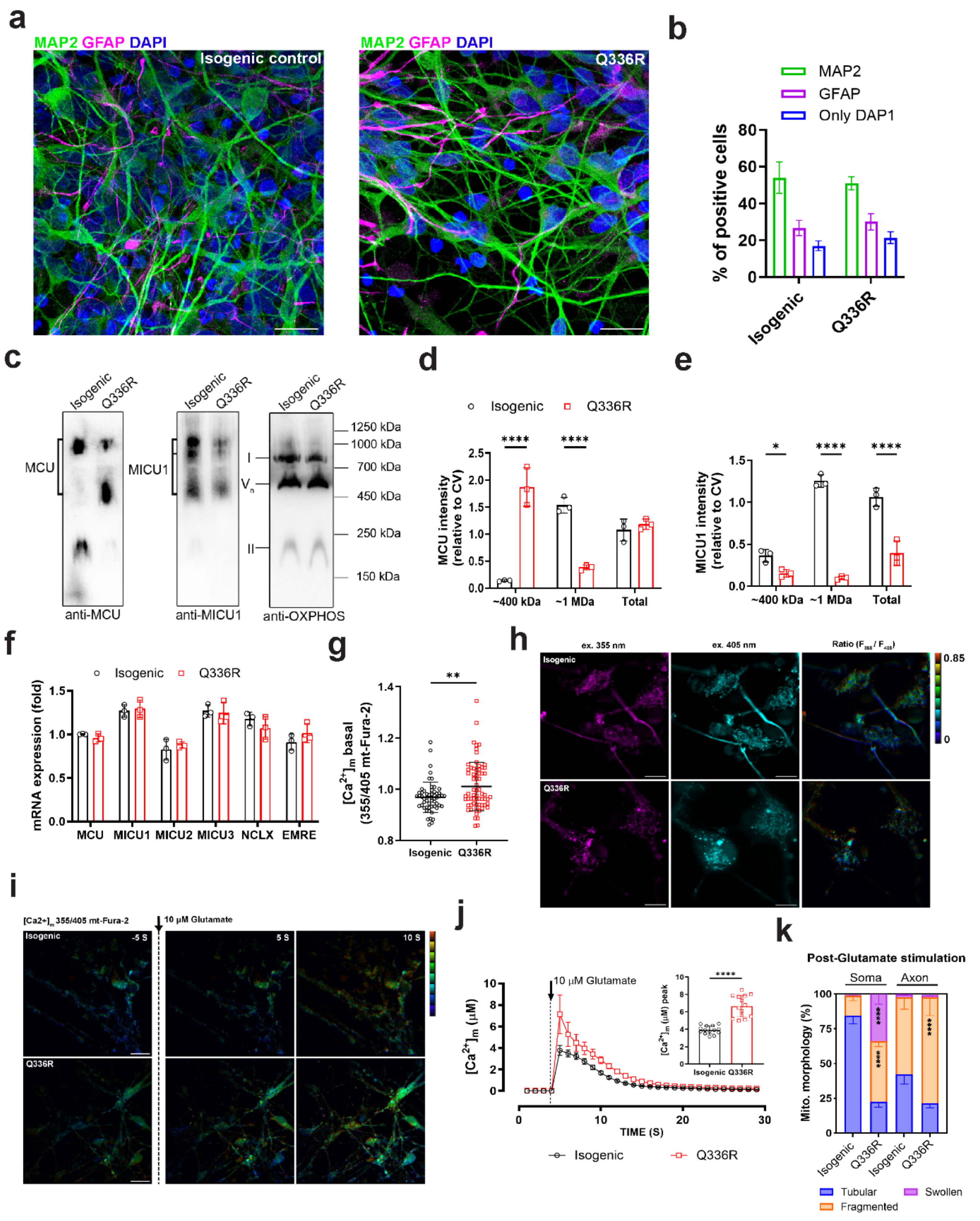

Supplementary Figure 8. Altered mitochondrial Ca^{2+} homeostasis in Q336R neurons. a Neural cell differentiated from NPCs were immunostained for the neuronal marker MAP2 and the glial marker GFAP. Scale bars, 20 µm. **B** MAP2 (green) and GFAP (magenta) fluorescence were used to determine the percentage of neuronal and glial cells, respectively, and DAPI (blue) was used to determine the total number of cells in each field of view, (n= 3). **c** Immunoblot analysis of native

MCU complex assembly, respiratory chain protein expression and supercomplex assembly in mitochondria isolated from isogenic and Q336R neurons, analyzed by blue native gel electrophoresis (BNGE) using the indicated antibodies. **d, e** Quantification of high and low-molecular weight MCU- and MICU1-containing complexes identified by BNGE in c, (n= 3, ****p= 2.31 × 10^{-7}, 1.59 × 10^{-5}, *p= 0.0, ****p= 4.50 × 10^{-9}, 3.86 × 10^{-6}). **f** mRNA levels of genes involved mitochondrial Ca^{2+} signaling were measured by qPCR using cDNA generated from isolated mRNA and normalized to isogenic control, (n= 3, p= 0.9969, 0.9764, 0.9950, 1.0000, 0.6429, 0.5883). **g** Quantification of resting mitochondrial Ca^{2+} concentration, $[Ca^{2+}]_m$ in isogenic and Q336 neurons before glutamate stimulation, calculated from traces in Fig. 8h and i, (n= 56 neurons, **p= 0.0084). **h** Representative mito-Fura-2 ratio images (F_{355}/F_{405}) of isogenic and Q336R neurons, showing a higher steady-state $[Ca^{2+}]_m$ and a more swollen mitochondrial morphology in Q336R neurons. Scale bars, 10 µm. **i** Representative mito-Fura-2 ratio images (F_{355}/F_{405}) for isogenic and Q336R neurons at the start of the experiment (t = -5 s) and at 5 s and 10 s after glutamate exposure, showing the rate of increase in $[Ca^{2+}]_m$ in each genotype. Scale bars, 50 µm. **j** Mean traces of $[Ca^{2+}]_m$ uptake measured in isogenic and Q336R neurons using the mt-AEQ plate reader assay in response to 10 µM glutamate. The inset shows the maximal $[Ca^{2+}]_m$ after stimulation, (n= 15 runs, ****p= 4.84 × 10^{-7}). **k** Quantitative morphometric analysis of mitochondria in somas and axons of isogenic and Q336R neurons treated with 10 µM glutamate or PBS for 12 h. All immunofluorescence images including representative images shown in Fig. 7m were classified into networked, fragmented and swollen mitochondria potted as a percentage of the total mitochondria (n= 3, ****p= 3.6 × 10^{-6}, 2.1 × 10^{-5}, 4.3 × 10^{-5}). Data in **b** and **d- k** are shown as mean ± SD with individual data points from independent experiments. Statistical analysis was performed using Welch's unpaired two-tailed t-test or two-way ANOVA with Šidák test.

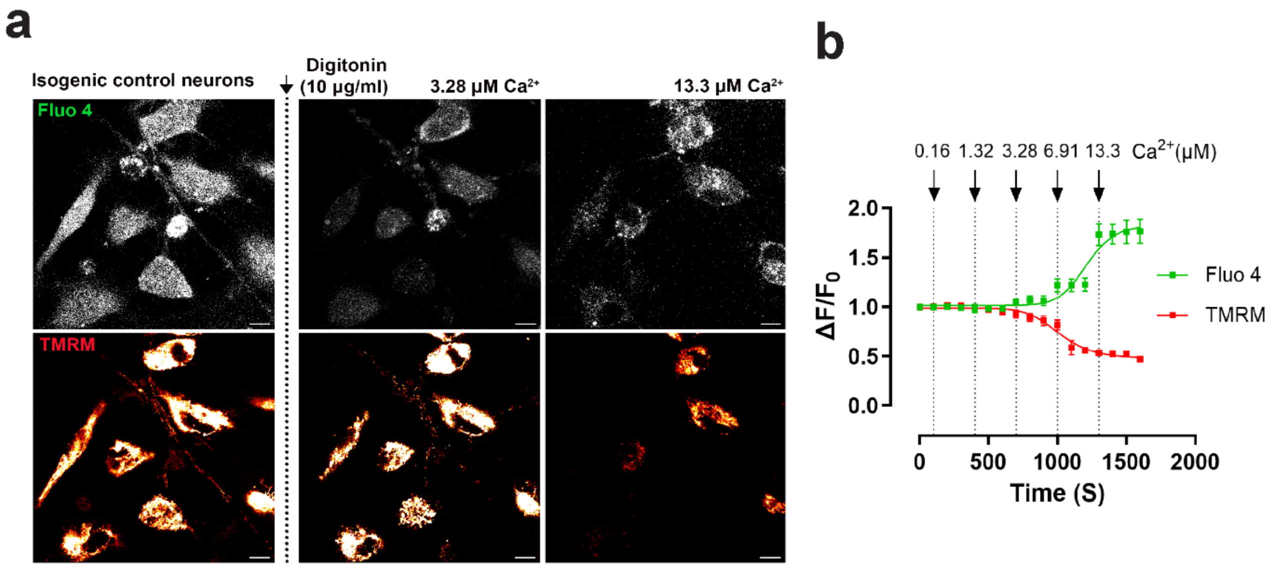

Supplementary Figure 9. Mitochondrial Ca^{2+} buffering capacity determined by simultaneous measurement of $[Ca^{2+}]_m$ and $\Delta\Psi_m$ in isogenic control neurons. a Representative confocal image of permeabilized isogenic control neurons showing changes in TMRM and Fluo-4 AM fluorescence in response to increasing extracellular Ca^{2+} concentrations (3.28 and 13.3 µM). Before digitonin permeabilization, Fluo-4 signal is predominantly cytosolic, whereas TMRM localizes to tubular mitochondria in both somas and axons. After permeabilization, the punctate staining pattern of both Fluo-4 and TMRM confirms mitochondrial localization of the dyes. **b** Mean ± SEM traces of in Fluo-4 fluorescence representing mitochondrial Ca^{2+} ($[Ca^{2+}]_m$) and TMRM fluorescence, representing mitochondrial membrane potential ($\Delta\Psi_m$), in response to increasing Ca^{2+} concentrations in the recording medium. Quantified data from one representative control experiment show the characteristic responses. The final free $[Ca^{2+}]$ in the medium is indicated for each addition (n= 8 runs).

SUPPLEMENTARY METHODS

hiPSCs derivation and culture

The iPSC lines, GM27291 and GM28930 used in this study were generated by Coriell Institute. iPSC cell line GM27291 was derived from GM26636 fibroblasts (Patient 1)[1] and iPSC cell line GM28930 is the isogenic control for the patient-derived line GM27291. iPSCs were thawed, expanded, and maintained using hESC-qualified matrigel-coated plates (356277, Corning) with mTeSR1 iPSC medium (85850, STEMCELL Technologies) as per the commercially available protocol from STEMCELL Technologies. The iPSCs were passaged when they reached approximately 70%–80% confluency using 0.5 mM EDTA (S311-500, Fisher Scientific) prepared in PBS (10010023, Thermo Fisher Scientific).

Differentiation of human iPSCs to neuronal networks

Cortical neuronal cultures were generated from human iPSCs[2]. Neural maintenance media (NMM) comprised a 1:1 mixture of DMEM/F-12 GlutaMAX (10565018, Thermo Scientific) with Neurobasal media (12348-017, Thermo Scientific), supplemented with 2.5 µg/ml insulin (I9278, Sigma-Aldrich), 1 mM L-glutamine (25030-024Thermo Scientific), 50 µm nonessential amino acids (NEAA) (11140-050, Thermo Scientific) , 50 µM β-mercaptoethanol (31350010, Gibco), 0.5x N-2 supplement (17502001, Thermo Scientific), 0.5x B-27 (17504001, Thermo Scientific), and 0.25% penicillin/streptomycin (15140122, Gibco). Neural induction media (NIM) consisted of NMM with 1 µM Dorsomorphin (3093, Tocris) and 10 µM SB431542 (1614, Tocris). In brief, iPSCs were

cultured as above transferred at a seeding ratio of 2:1 to hESC-qualified Matrigel coated wells, in mTeSR1 supplemented with 10 μM ROCK inhibitor (1254/10, Bio-techne). Cells were incubated at 37°C for 24 h-48 h to reach 100% confluence. Cell layers were gently washed with PBS and medium replaced with Neural Induction Media (NIM), which was then replaced daily, until a dense neuro-epithelial sheet formed at around 8-12 days after neural induction. At this point, cells were passaged using 1 mg/ml dispase (17105041, Thermo Scientific) in NIM and lifted as aggregates. These aggregates were washed and seeded at a ratio of 1:2 in fresh NIM, onto laminin (L2020-1MG, Sigma-Aldrich) wells. Cells were incubated overnight to allow attachment, and clumps that had not been attached were transferred to fresh laminin coated wells. Upon the formation of neural rosettes, culture medium was replaced with NMM supplemented with 20 ng/ml recombinant human FGF2 (100-18B, PeproTech), which was changed every 48 h. FGF2 was withdrawn from the culture medium after 4 days, and cells were expanded using dispase to lift neural rosettes. On day 25 (±1 day) after induction, cultures were dissociated into single cells using Accutase (A1110501, Thermo Scientific) and seeded in NMM at a ratio of 1:1 in fresh laminin coated wells. From this point onwards, cells were expanded as single cell suspensions with Accutase, and media was replaced at least every 48 h. At ~ day 36 of neural induction, iPSC-NPCs (neural progenitor cells) were seeded in poly-L-lysine (P4707, Sigma) and laminin coated plates at a density of $5x10^4$ cells/cm^2 for terminal neuronal differentiation, and NMM was replaced at least every 48 h. Neuronal cultures were matured until at least day 62.

For neuronal characterization, Neuronal cultures in glass-bottom 96-well plate (655892, SensoPlate™) were fixed for 15 min at room temperature using a 4% paraformaldehyde solution, washed and permeabilized for 5 min with 0.1% Triton X-100 (AC215682500, Fisher Scientific) in PBS. Fixed cells were blocked with 3% BSA (A6003, Sigma) in PBS to block the nonspecific bindings for 60 min. Cells were then incubated overnight at 4°C with the GFAP and MAP2 primary antibodies prepared in blocking solution. The following day, the cells were washed with PBS and incubated with secondary antibodies, Alexa Fluor 488 or Alexa Fluor 568 based on the primary antibody host species, for 1 h. Then, the cells were washed with PBS and incubated with 4,6-diamidino-2-phenylindole (DAPI) solution (1:10,000, D1306, Thermo Scientific) for 5 min to detect all nuclei and imaged using confocal microscope as described below. Details of the antibodies used in this study can be found in Supplementary Table 3.

RNA-sequencing

Fibroblasts for RNA-sequencing were seeded at a density of 1×10^6 cells/dish and cultured in 10 cm dishes with regular media before harvesting on day two. RNA sequencing was performed at the UCL Genomics Core Facility using the Mag-Bind® Total RNA 96 Kit for RNA-sequencing polyA capture on the Illumina NextSeq 2000 P2 sequencer. Reads were mapped and analyzed by the SARTools R

package. Differential expression analysis was carried out with the Bioconductor package DESeq2 (v.1.48.0)[3]. Genes were annotated using the org.Hs.eg.db package (Genome wide annotation for Human, v.3.21.0) and differentially expressed genes (DEG) with an adjusted p-value cut-off of 0.05 were identified as statistically significant. Gene ontology (GO) and Kyoto Encyclopedia of Genes and Genomes (KEGG) enrichment analysis were performed using the ClusterProfiler package[4]. Results of DEG analysis were visualized with heatmaps plotted in ClustVis[5].

Transmission Electron Microscopy

Fibroblasts cultured on coverslips were fixed in EM fixative (2% glutaraldehyde + 2% paraformaldehyde in 0.1M sodium cacodylate) for 1 h followed by washings with 0.1M Cacodylate Buffer. The Coverslips were then fixed in 1% osmium tetroxide and 1% potassium ferricyanide in 0.1M sodium cacodylate, followed by sequential dehydration in ethanol. The coverslips were embedded in Epoxy Resin (Araldite CY212) Kit (Agar Scientific Ltd.) according to the standard protocol. Embedded coverslips were sectioned to 50 nm using an ultra-microtome fitted with a diamond knife, mounted onto TEM compatible copper grids. The grids were then stained with Lead Citrate for 3 min before proceeding for Imaging. Images were acquired using Jeol 1400 Transmission Electron Microscope and Gatan software. Images were taken at a magnification of 1200X (digital magnification). Interfaces between ER and mitochondria were segmented using DeepMIB software[6] analyzed using a custom ImageJ script: https://sites.imagej.net/MitoCare/ .

ROS measurements

The rate of intracellular ROS production in fibroblasts was measured using a superoxide indicator, dihydroethidium (DHE; D11347, Thermo Scientific) as described previously[7]. Following the measurements of fluorescence intensity, cells were stained with Hoechst 33342 (62249, Thermo Scientific) for 10 min to label and count the numbers of cell nuclei representing cell numbers in each well using an automated fluorescent image acquisition system (ImageXpress MicroXL). Subsequently, the fluorescence intensity was normalized based on the relative cell numbers obtained.

Blue native gel electrophoresis (BNGE) and immunoblotting

Mitochondria from the fibroblasts were isolated using mitochondria isolation buffer 1 (MIB1; 225 mM Mannitol, 75 mM Sucrose, 5 mM HEPES, 1 mM EGTA and 1 mg/ml fatty acid free BSA) and MIB2 (same as MIB1 but without BSA) according to the method described previously [8]. Mitochondria isolation from the neuronal cultures were performed similarly, with the exception of homogenization step, performed using a Dounce tissue grinder tube. Protein concentration of the isolated mitochondria was quantified using the Pierce BCA Assay Kit (23227, Thermo Scientific)

and an equivalent amounts of total protein (50 μg) was solubilized with digitonin followed by centrifugation at 20,000g for 20 min at 4°C. Digitonin-solubilized mitochondria were separated on 3–12% NativePAGE Bis-Tris gels (BN1001, Invitrogen) and electroblotted onto PVDF membrane (1620175, Bio-Rad) according to the manufacturer's instructions. Membranes were then blocked and probed with indicated primary antibodies as described above. Quantification of the protein bands was performed using ImageJ. Details of all the antibodies used in this study can be found in Supplementary Table 3.

Quantitative reverse transcription PCR (RT-qPCR)

Total RNA was extracted from fibroblasts and neuronal culture using the RNeasy Plus Mini Kit (74104, Qiagen) according to the manufacturer's instructions. Quality check and the quantification of isolated mRNA was done on the Nanodrop 2000c (ND-2000, Thermo Scientific). cDNA was synthesized from 1 μg of total RNA using SuperScript IV First-Strand Synthesis System Kit (18091050, Invitrogen) and quantitative PCRs was performed using SYBR Green JumpStart Taq ReadyMix (Sigma-Aldrich) on a CFX96 Real-Time PCR Detection System (Bio-Rad). Data were analysed using the comparative $2^{-\Delta\Delta Ct}$ method. Ct of the gene of interest was normalized to that of β-actin.

For the quantitative analysis of the relative mtDNA copy number, total genomic DNA was extracted from fibroblasts using the DNeasy Blood & Tissue Kit (69506, Qiagen) and the quantitative PCR was performed similarly with primers for the mtDNA tRNALeu (UUR) and for the nuclear B2M (β-2-microglobulin) to determine the relative mtDNA copy number of cells [9]. The following equation was used to determine the relative mitochondrial DNA content, 2 x $2^{\Delta Ct}$, where ΔCt is nuclear DNA Ct value subtracted by mtDNA Ct value. All primer pairs used can be found in Supplementary Table 4.

Mitochondria morphology analysis

Morphometric analysis of TMRM-labelled or TOM20-immunolabelled raw confocal images was performed using a 2D cell segmentation model, MitoSegNet[10]. Neuronal somas and axons were manually segmented in ImageJ using β-tubulin III staining for morphological distinction. Briefly, images were pre-processed to 8-bit format and mitochondria were first segmented using the MitoS basic toolbox (https://github.com/MitoSegNet). Segmentation masks were then run on MitoA analyzer tool for the quantification of the morphological features broadly categorized into shape descriptors such as area, eccentricity and perimeter and the network descriptors such as branch length, number of branches and curvature index. The shape descriptors values were then used to calculate the percentage of elongated, fragmented or swollen mitochondrial pool.

Mitochondrial Ca^{2+} concentration measurements with aequorin

$[Ca^{2+}]_m$ measurements in fibroblasts and neuronal cultures were carried out using the mitochondria-targeted luminescent aequorin probe, mtAEQ as previously described[11]. Fibroblasts were seeded at a density of 2×10^4 cells/well on white 96-well plate (6005680, PerkinElmer) and cultured for 1-2 days. Similarly, iPSC-derived NPCs were seeded at a density of 5×10^4 cells/well on white 96-well plate coated with poly-L-lysine and laminin for terminal neuronal differentiation and maturation using the method describe above. Two days before the experiment, cells were transduced with mtAEQ adenovirus. Following incubation, media was replaced with 5 µM coelenterazine (C2944, Invitrogen) in Krebs Ringer Buffer (125mM NaCl, 5.5mM D-Glucose, 5 mM KCl, 20mM HEPES, 1 mM Na_3PO_4, 1mM Glutamine, 100mM Pyruvate, and 1.2 mM $CaCl_2$ at pH 7.4). The plate was then incubated in the dark for 2 h at 37 °C. Baseline luminescence signals were acquired using a plate reader (CLARIOstar, BMG Labtech) every 1 s followed by fluidic additions of either 10 µM histamine or 10 µM glutamate using integrated syringe injectors. At the end of each experiment, maximal aequorin signal was obtained by permeabilizing the cells with 1 mM digitonin and exposing the cells to a saturating Ca^{2+} concentration of 10 mM $CaCl_2$. For analysis, luminescence values were converted into Ca^{2+} concentration as previously described[11].

Calcium retention capacity assay

The capacity of isolated mitochondria to accumulate Ca^{2+} until the mPTP opens was determined using the method described earlier[12]. Isolated mitochondria from the fibroblasts (0.5mg/ml), using the method described above, were resuspended in the RB2 (75 mM D-mannitol, 25 mM sucrose, 5 mM KH_2PO_3, 20 mM Tris-HCl, 100 mM KCl, and 0.1% BSA fatty acids free at pH 7.4) supplemented with 10 mM succinate and 1 µM rotenone to energize the mitochondria. 100 µl of the mitochondrial suspension was plated in a glass-bottom 96-well plate in triplicate for each condition. Extramitochondrial Ca^{2+} levels were quantified by measuring fluorescence intensity of the Ca^{2+}-sensitive dye, Calcium Green-5N (C3737, Invitrogen) at 1µM concentration The fluorescence intensity was recorded using a plate reader (CLARIOstar, BMG Labtech) at 30℃ with the following filters: ex/em: 480 nm/520 nm. Ca^{2+} additions were achieved using integrated syringe injectors, where subsequent 10 µl additions of 50 µM $CaCl_2$ were added for a total of 12 injections. The area under the curve was used as a measure of extramitochondrial Ca^{2+}, which was expressed as a proportion of total Ca^{2+} added (Ca^{2+} free condition was used for background subtraction). This value was used to calculate the proportion of buffered Ca^{2+}, and subsequent percentage inhibitions were calculated compared to untreated.

SUPPLEMENTARY TABLE 1

***EPG5* mutations (NM_020964.2) in patient-derived fibroblasts**

**Patients**	**Variant 1**			**Variant 2**			**Source** (Clinical presentation, outcome and Family/patient ID)
	**Nucleotide**	**amino acid**	**Exon**	**Nucleotide**	**amino acid**	**Exon**	
Patient 1 (Pat1)	c.1007A>G	p.Q336R	2	c.1007A>G	p.Q336R	2	Cullup et al., 2013[13], Patient ID- 7.1
Patient 2 (Pat2)	c.4862G>A	p.R1621G	28	c.4862G>A	p.R1621G	28	Vansenne et al., 2022[14], Patient ID- 1.1
Patient 3 (Pat3)	c.895C>T	p. R299*	2	c.5479C>G	p.P1827A	31	Dafsari et al., 2024[15], Patient ID- 82.1
Patient 4 (Pat4)	c.4952+1G>A	p.F1604Gfs*20	28	c.4952+1G>A	p.F1604Gfs*20	28	Byrne et al., 2016[16], Patient ID- 4.1

SUPPLEMENTARY TABLE 2

Upstream analysis of transcription regulators in patient fibroblasts

Transcription regulators	Pathway responsive genes[17]	Z-score	adj. *p*-value (BH-adjusted p values)
**PPARGC1A**	FASTK, CYP7B1, APOLD1, NFKBIA, PTGS2, PDK4, BCL2L1	1.41141849	0.030074675
**ATF4**	BEX4, MTHFD1, MTHFD2, ATF3, IDH1, PHGHD, RGS2, PIM1, PSHP, EIF4EBP1, EIF1B, DNAJB9, HSPA2, HSP70, HSP90B1, SEC61A2, RELB, NFKB1, TNFSF10B, EIF2A	1.457997196	0.031461968
**RELA**	ABCB4, BCL2, ABCA1, BIRC5, BCL2L1, CCL2, CXCL10, SERPINE2, SOD2, TRAF2, CFLAR,	1.859075413	0.028959004
**STAT1**	CCL2, CXCL10, FGF2, IFIT3, IL6, IRF7	1.437937718	0.039748444
**IRF3**	CCL2, CXCL1O, ATF4	1.729626042	0.01774614
**KLF5**	BIRC5, CCNB1, CCND1, MMP3, MYH10, ITGB2,	1.566385318	0.026121499
**MAF**	CCND2, GCLC, IL4, MMP13	1.811069918	0.022278662
**FOSL1**	MMP1, PLAUR, ITGB3, CLU	1.740310439	0.03584421

SUPPLEMENTARY TABLE 3

Key resources table

Reagent or resource	Source	Identifier
**Antibodies**		
Rabbit pAb anti-EPG5 (1:1000)	Abcam	Cat# ab122186
Mouse mAb anti-β-actin (1:10000)	Cell Signaling Technology	Cat# 3700
Mouse Ab anti-OxPhos cocktail (1:1000)	Invitrogen	Cat# 45-8199
Rabbit mAb anti-TOM20 (1:5000 IB, 1:200 IF)	Abcam	Cat# ab186735
Mouse mAb anti-MCU (1:1000)	Sigma-Aldrich	Cat# AMAB91189
Rabbit pAb anti-MCUB (1:1000)	Proteintech	Cat# 20387-1-AP
Rabbit pAb anti-MICU1 (1:1000)	Thermo Scientific	Cat# HPA037480
Rabbit pAb anti-MICU2 (1:1000)	Sigma-Aldrich	Cat# HPA045511
Rabbit pAb anti-MICU3 (1:1000)	Thermo Scientific	Cat# PA5-55177
Rabbit pAb anti-EMRE (1:1000)	Abcam	Cat# ab157387
Rabbit pAb anti-NCLX (1:1000)	Sigma-Aldrich	Cat# SAB2102181
Mouse mAb anti-ATP5A (1:1000)	Abcam	Cat# ab14748
Rabbit pAb anti-phospho PDH E1α (Ser293) (1:1000)	Sigma-Aldrich	Cat# AP1062

Mouse mAb anti- PDH E1α (1:5000)	Abcam	Cat# ab110330
Rabbit mAb anti-HA (1:1000)	Abcam	Cat# ab236632
Rabbit pAb anti-STAT1 (1:1000)	Cell Signaling Technology	Cat# 9172S
Rabbit mAb anti-phospho STAT1 (Tyr701) (1:1000)	Cell Signaling Technology	Cat# 9167S
Rabbit mAb anti-TBK1 (1:1000)	Cell Signaling Technology	Cat# 3504S
Rabbit mAb anti-phospho TBK1 (Ser172) (1:1000)	Cell Signaling Technology	Cat# 5483S
Rabbit mAb anti-STING (1:1000)	Cell Signaling Technology	Cat# 13647S
Rabbit pAb anti-IRF3 (1:1000)	Abcam	Cat# ab25950
Rabbit mAb anti-phospho IRF3 (Ser396) (1:1000)	Cell Signaling Technology	Cat# 29047S
HRP-Goat Anti-Rabbit IgG (H+L) (1:5000-1:10000)	Jackson ImmunoResearch	Cat# 111-035-045
HRP-Goat Anti-Mouse IgG (H+L) (1:5000)	Jackson ImmunoResearch	Cat# 315-035-045
Rabbit mAb anti-cGAS (1:100 IF)	Cell Signaling Technology	Cat# 15102S

Rabbit pAb anti-Citrate synthetase (1:100 IF)	Abcam	Cat# ab96600
Mouse mAb anti-DNA (1:200 IF)	Sigma-Aldrich	Cat# CBL186
Mouse mAb anti-Cytochrome C (1:200 IF)	BD Pharmingen	Cat# 556432
Mouse mAb anti-Beta-Tubulin III (TUJ1) (1:100 IF)	STEMCELL Technologies	Cat# 60052
Rabbit pAb anti-GFAP (1:100 IF)	Sigma-Aldrich	Cat# AB5804
Chicken pAb anti-MAP2 (1:100 IF)	Abcam	Cat# ab92434
Alexa Fluor 647 Donkey anti-Rabbit IgG (H+L) (1:1000)	Thermo Scientific	Cat# A-31573
Alexa Fluor 488 Donkey anti-Mouse IgG (H+L) (1:1000)	Thermo Scientific	Cat# A-21202
Alexa Fluor 568 Donkey anti-Rabbit IgG (H+L) (1:1000)	Thermo Scientific	Cat# A-10042
Alexa Fluor 488 Goat anti-Rabbit IgG (H+L) (1:1000)	Thermo Scientific	Cat# A-11008
Alexa Fluor 568 Goat anti-Mouse IgG (H+L) (1:1000)	Thermo Scientific	Cat# A-11004
Alexa Fluor 647 Goat anti-Chicken IgG (H+L) (1:1000)	Thermo Scientific	Cat# A-21449
**Commercial Kit Assays**		

MycoAlert Mycoplasma Detection Kit	Lonza	Cat# LT07-118
Seahorse XF Cell Mito Stress Test Kit	Agilent	Cat# 103015-100
Seahorse XFe96/XF Pro FluxPak	Agilent	Cat# 103793-100
Human Dermal Fibroblasts Nucleofector Kit	Lonza	Cat# VPI-1002
NativePAGE Novex Bis-Tris Gel System	Thermo Scientific	Cat# BN2007, BN2008
**Experimental Models: Cell Lines**		
Control Fibroblasts	This paper and Logan et al., 2014[18]	PMID: 24336167 and MRC CNMD Biobank London
Patient Fibroblasts	This paper	Table 1
BJ-hTERT	ATCC	RRID: CVCL_6573
Isogenic iPSCs	Coriell Institute	RRID: GM28930
Q336R iPSCs	Coriell Institute and Mitchell et al., 2022[1]	RRID: GM27291 and PMID: 35700637
**Critical chemicals and reagents**		
JP1-138	Pingitore et al., 2024[19]	PMID: 38985859
Mito-Fura-2 AM (v2.3)	Pendin et al., 2019[20]	PMID: 31132197

**siRNA and constructs**		
EPG5 human siRNA oligo duplex	Origene	SR324765
pTRIP-CMV-mTagBFP2-2A-FLAG-ntcGAS	Addgene[21]	Cat# 102603
Ade-mtaequorin (mt-AEQ)	Gift from Rosario Rizzuto lab[22]	PMID: 27138568
EPG5-GFP (NM_020964)	Origene	RG219254
pCMV6-EPG5 untagged (NM_020964)	Origene	SC318729
MICU1-HA	Gift from Anna Raffaello lab[23]	PMID: 30242232
**Software and algorithms**		
FIJI (ImageJ); Version 1.54p	http://fiji.sc[24]	RRID: SCR_002285
Prism 10	GraphPad	RRID: SCR_002798
MitoSegNet	https://github.com/MitoSegNet[10]	PMID: 33083756
Image Lab software; Version 6.1	Bio-Rad	RRID: SCR_014210
Microscopy Image Browser; Version 2.81	https://mib.helsinki.fi/[6]	RRID:SCR_016560
Imaris Version 9.8	Bitplane	RRID:SCR_007370

MetaMorph Microscopy Automation and Image Analysis Software; Version 7.8.12.0	Molecular Devices	RRID:SCR_002368
MetaFluor Fluorescence Ratio Imaging Software; Version 7.8.12.0	Molecular Devices	RRID:SCR_014294
Zen Black 2.3 (Zeiss) Microscopy Software	Carl Zeiss	RRID:SCR_013672
Seahorse Wave; Version 2.4.0.60	Agilent	RRID:SCR_014526
R Project	http://www.r-project.org/	RRID:SCR_001905

SUPPLEMENTARY TABLE 4

Primer pairs used in this study.

Target	Forward	Reverse	Source
mitochondria	CACCCAAGAACAGGGTTTGT	TGGCCATGGGTATGTTGTTA	Rooney, J. P. et al., 2015[9]
nucleus	TGCTGTCTCCATGTTTGATGTATCT	TCTCTGCTCCCCACCTCTAAGT	
ACTB	CACCATTGGCAATGAGCGGTTC	AGGTCTTTGCGGATGTCCACGT	Thermo Scientific
EPG5	CCTTCTGTATCTTCACCGTCCG	GAAGTCAGCCACCTCGGTCAAA	
NCLX	ATGGTGGCTGTGTTCCTGACCT	GGTGCAGAGAATCACAGTGACC	
MCU	CAGCACTGTTGTGCCCTCTGAT	GGCTTGAGTGTGAACTGACAGC	
MICU1	GACAGTGGCTAAAGTGGAGCTC	CCTCTCATCAGCCGTTGCTTCA	
MICU2	GGATGGCAGTTTTACAGTCTCCG	GAAGAGGAAGTCTCGTGGTGTC	
MICU3	CACTGATGGCAATGAGATGGTGG	GACGCAGCATTGCACGCTTTTC	
EMRE	GCCTAGCTTGAGGAAAGATGGC	ATGGAGAACACACGCAGAAGGC	

IL1B	CCACAGACCTTCCAGGAGAATG	GTGCAGTTCAGTGATCGTACAGG	
IL6	AGACAGCCACTCACCTCTTCAG	TTCTGCCAGTGCCTCTTTGCTG	
TNF	CTCTTCTGCCTGCTGCACTTTG	ATGGGCTACAGGCTTGTCACTC	
CCL2	AGAATCACCAGCAGCAAGTGTCC	TCCTGAACCCACTTCTGCTTGG	
CXCL10	GGTGAGAAGAGATGTCTGAATCC	GTCCATCCTTGGAAGCACTGCA	
IFIT3	CCTGGAATGCTTACGGCAAGCT	GAGCATCTGAGAGTCTGCCCAA	
ISG15	CTCTGAGCATCCTGGTGAGGAA	AAGGTCAGCCAGAACAGGTCGT	

REFERENCES

1 Mitchell, M. W., Grandizio, C., Turan, N. & Requesens, D. V. An induced pluripotent stem cell line (CIMRi001-A) from a Vici syndrome donor with a homozygous recessive c.1007A>G (p.Q336R) mutation in the EPG5 gene. *Stem Cell Res* **63**, 102833, doi:10.1016/j.scr.2022.102833 (2022).

2 Shi, Y., Kirwan, P. & Livesey, F. J. Directed differentiation of human pluripotent stem cells to cerebral cortex neurons and neural networks. *Nat Protoc* **7**, 1836-1846, doi:10.1038/nprot.2012.116 (2012).

3 Love, M. I., Huber, W. & Anders, S. Moderated estimation of fold change and dispersion for RNA-seq data with DESeq2. *Genome Biol* **15**, 550, doi:10.1186/s13059-014-0550-8 (2014).

4 Yu, G., Wang, L. G., Han, Y. & He, Q. Y. clusterProfiler: an R package for comparing biological themes among gene clusters. *OMICS* **16**, 284-287, doi:10.1089/omi.2011.0118 (2012).

5 Metsalu, T. & Vilo, J. ClustVis: a web tool for visualizing clustering of multivariate data using Principal Component Analysis and heatmap. *Nucleic Acids Res* **43**, W566-570, doi:10.1093/nar/gkv468 (2015).

6 Belevich, I. & Jokitalo, E. DeepMIB: User-friendly and open-source software for training of deep learning network for biological image segmentation. *PLoS Comput Biol* **17**, e1008374, doi:10.1371/journal.pcbi.1008374 (2021).

7 Chung, C. Y. & Duchen, M. R. A Plate Reader-Based Measurement of the Cellular ROS Production Using Dihydroethidium and MitoSOX. *Methods Mol Biol* **2497**, 333-337, doi:10.1007/978-1-0716-2309-1_24 (2022).

8 Singh, K. & Duchen, M. R. Analysis of Organization and Activity of Mitochondrial Respiratory Chain Complexes in Primary Fibroblasts Using Blue Native PAGE. *Methods Mol Biol* **2497**, 339-348, doi:10.1007/978-1-0716-2309-1_25 (2022).

9 Rooney, J. P. *et al.* PCR based determination of mitochondrial DNA copy number in multiple species. *Methods Mol Biol* **1241**, 23-38, doi:10.1007/978-1-4939-1875-1_3 (2015).

10 Fischer, C. A. *et al.* MitoSegNet: Easy-to-use Deep Learning Segmentation for Analyzing Mitochondrial Morphology. *iScience* **23**, 101601, doi:10.1016/j.isci.2020.101601 (2020).

11 Bonora, M. *et al.* Subcellular calcium measurements in mammalian cells using jellyfish photoprotein aequorin-based probes. *Nat Protoc* **8**, 2105-2118, doi:10.1038/nprot.2013.127 (2013).

12 Bhosale, G. & Duchen, M. R. Investigating the Mitochondrial Permeability Transition Pore in Disease Phenotypes and Drug Screening. *Curr Protoc Pharmacol* **85**, e59, doi:10.1002/cpph.59 (2019).

13 Cullup, T. *et al.* Recessive mutations in EPG5 cause Vici syndrome, a multisystem disorder with defective autophagy. *Nat Genet* **45**, 83-87, doi:10.1038/ng.2497 (2013).

14 Vansenne, F. *et al.* Phenotypic expansion of EGP5-related Vici syndrome: 15 Dutch patients carrying a founder variant. *Eur J Paediatr Neurol* **41**, 91-98, doi:10.1016/j.ejpn.2022.11.003 (2022).

15 Dafsari, H. S. *et al.* Mutations in the Key Autophagy Tethering Factor EPG5 Link Neurodevelopmental and Neurodegenerative Disorders Including Early-Onset Parkinsonism. *Ann Neurol*, doi:10.1002/ana.78013 (2025).

16 Byrne, S. *et al.* EPG5-related Vici syndrome: a paradigm of neurodevelopmental disorders with defective autophagy. *Brain* **139**, 765-781, doi:10.1093/brain/awv393 (2016).

17 Han, H. *et al.* TRRUST v2: an expanded reference database of human and mouse transcriptional regulatory interactions. *Nucleic Acids Res* **46**, D380-D386, doi:10.1093/nar/gkx1013 (2018).

18 Logan, C. V. *et al.* Loss-of-function mutations in MICU1 cause a brain and muscle disorder linked to primary alterations in mitochondrial calcium signaling. *Nat Genet* **46**, 188-193, doi:10.1038/ng.2851 (2014).

19 Pingitore, V. *et al.* Delocalized quinolinium-macrocyclic peptides, an atypical chemotype for CNS penetration. *Sci Adv* **10**, eado3501, doi:10.1126/sciadv.ado3501 (2024).

20 Pendin, D. *et al.* A Synthetic Fluorescent Mitochondria-Targeted Sensor for Ratiometric Imaging of Calcium in Live Cells. *Angew Chem Int Ed Engl* **58**, 9917-9922, doi:10.1002/anie.201902272 (2019).

21 Gentili, M. *et al.* Transmission of innate immune signaling by packaging of cGAMP in viral particles. *Science* **349**, 1232-1236, doi:10.1126/science.aab3628 (2015).

22 Tosatto, A. *et al.* The mitochondrial calcium uniporter regulates breast cancer progression via HIF-1alpha. *EMBO Mol Med* **8**, 569-585, doi:10.15252/emmm.201606255 (2016).

23 Matteucci, A. *et al.* Parkin-dependent regulation of the MCU complex component MICU1. *Sci Rep* **8**, 14199, doi:10.1038/s41598-018-32551-7 (2018).

24 Schindelin, J. *et al.* Fiji: an open-source platform for biological-image analysis. *Nat Methods* **9**, 676-682, doi:10.1038/nmeth.2019 (2012).
