# Supplementary material for: Pathogenic variants in the autophagy-tethering factor EPG5 drive neurodegeneration through mitochondrial dysfunction and innate immune activation
Source: Nat Commun. 2026 May 26;17:6887. doi: 10.1038/s41467-026-73538-7 (PMC13388713; doi:10.1038/s41467-026-73538-7)
Supplement: Supplementary file 2 — Description of Additional Supplementary File [file 41467_2026_73538_MOESM2_ESM.pdf]

Description of Additional Supplementary Files

Supplementary Movie 1:

Time-lapse imaging of mitochondrial membrane potential [$\Delta\Psi m$], mitochondrial calcium [Ca2+]m and mtDNA dynamics in response to histamine in control 1 fibroblasts. Control 1 fibroblasts colabelled with TMRM (red), mito-Fura-2 AM (pseudocoloured ratiometric image, blue to red LUT, range 0-0.85) and PicoGreen (green) were imaged every 12.5 s to simultaneously monitor changes in $\Delta\Psi m$, [Ca2+]m and mtDNA dynamics, respectively, in response to successive application of 10 µM and 20 µM histamine. Movie playback: 30 fps. Scale bar: 10 µm

Supplementary Movie 2: Time-lapse imaging of mitochondrial membrane potential ($\Delta\Psi m$), mitochondrial calcium [Ca2+]m and mtDNA extrusion in response to 10 µM histamine in patient 1 fibroblasts. Patient 1 fibroblasts co-labelled with mito-Fura-2 AM (pseudocoloured ratiometric image, blue to red LUT, range 0-0.85), TMRM (red) and PicoGreen (green) were imaged every 12.5 s to simultaneously monitor the increase in [Ca2+]m, collapse of $\Delta\Psi m$, and mtDNA extrusion, respectively, in response to 10 µM histamine challenge. Movie playback 30fps. Scale bar, 10 µm.

Supplementary Movie 3: Time-lapse imaging of mitochondrial membrane potential ($\Delta\Psi m$), mitochondrial calcium [Ca2+]m and mtDNA dynamics in response to 10 µM histamine in patient 1 fibroblasts pretreated with Ru360. Patient 1 fibroblasts pretreated with Ru360 and co-labelled with TMRM (red), mitoFura-2 AM (pseudocoloured ratiometric image, blue to red LUT, range 0-0.85), and PicoGreen (green) were imaged every 12.5 s to simultaneously monitor the change in $\Delta\Psi m$, [Ca2+]m and mtDNA extrusion, respectively, in response to 10 Mm histamine challenge. Movie playback 30fps. Scale bar, 10 µm.
