# Supplementary material for: Pathogenic variants in the autophagy-tethering factor EPG5 drive neurodegeneration through mitochondrial dysfunction and innate immune activation
Source: Nat Commun. 2026 May 26;17:6887. doi: 10.1038/s41467-026-73538-7 (PMC13388713; doi:10.1038/s41467-026-73538-7)
Supplement: Supplementary file 6 — Reporting Summary [file 41467_2026_73538_MOESM6_ESM.pdf]

nature portfolio

Corresponding author(s): Michael R Duchen

Last updated by author(s): Apr 12, 2026

Reporting Summary

Nature Portfolio wishes to improve the reproducibility of the work that we publish. This form provides structure for consistency and transparency in reporting. For further information on Nature Portfolio policies, see our Editorial Policies and the Editorial Policy Checklist.

Statistics

For all statistical analyses, confirm that the following items are present in the figure legend, table legend, main text, or Methods section.

n/a	Confirmed	
☐	☒	The exact sample size (*n*) for each experimental group/condition, given as a discrete number and unit of measurement
☐	☒	A statement on whether measurements were taken from distinct samples or whether the same sample was measured repeatedly
☐	☒	The statistical test(s) used AND whether they are one- or two-sided<br>*Only common tests should be described solely by name; describe more complex techniques in the Methods section.*
☒	☐	A description of all covariates tested
☐	☒	A description of any assumptions or corrections, such as tests of normality and adjustment for multiple comparisons
☐	☒	A full description of the statistical parameters including central tendency (e.g. means) or other basic estimates (e.g. regression coefficient) AND variation (e.g. standard deviation) or associated estimates of uncertainty (e.g. confidence intervals)
☐	☒	For null hypothesis testing, the test statistic (e.g. *F*, *t*, *r*) with confidence intervals, effect sizes, degrees of freedom and *P* value noted<br>*Give P values as exact values whenever suitable.*
☒	☐	For Bayesian analysis, information on the choice of priors and Markov chain Monte Carlo settings
☒	☐	For hierarchical and complex designs, identification of the appropriate level for tests and full reporting of outcomes
☐	☒	Estimates of effect sizes (e.g. Cohen's *d*, Pearson's *r*), indicating how they were calculated

Our web collection on statistics for biologists contains articles on many of the points above.

Software and code

Policy information about availability of computer code

Data collection	CFX Maestro 2.0 (Bio-Rad); version for qPCR, Seahorse Wave 2.4.0.60 (Agilent) for the Seahorse assay, Image Lab (6.0.1.34) for immunoblotting, Zen Black 2.3 (Zeiss) for fixed and live cell confocal imaging, MetaFluor Fluorescence Ratio Imaging Software 7.8.12.0 for live cell imaging, Gatan software for transmission electron microscopy, CLARIOstar (5.70) for plate-based luminescence and fluorescence recording and the SARTools R package for RNA-seq.
Data analysis	CFX Maestro 2 (Bio-Rad) for qPCR, Seahorse Wave Desktop 2.6 (Agilent) for the Seahorse assay, FIJI (ImageJ) Version 1.54p for immunoblotting and imaging, Imaris Version 9.8 for image segmentation, quantification and 3D rendering, MitoSegNet for mitochondrial morphometric analysis, Microscopy Image Browser; Version 2.81 for segmentation and quantification of TEM images, MetaMorph Microscopy Automation and Image Analysis Software 7.8.12.0 for ratiometric imaging, R for RNA-seq and Graph Pad Prism 10 for all statistical analyses.

For manuscripts utilizing custom algorithms or software that are central to the research but not yet described in published literature, software must be made available to editors and reviewers. We strongly encourage code deposition in a community repository (e.g. GitHub). See the Nature Portfolio guidelines for submitting code & software for further information.

Data

Policy information about availability of data

All manuscripts must include a data availability statement. This statement should provide the following information, where applicable:

- Accession codes, unique identifiers, or web links for publicly available datasets
- A description of any restrictions on data availability
- For clinical datasets or third party data, please ensure that the statement adheres to our policy

The RNA-seq datasets generated and analyzed during the current study are available in the GEO repository GSE316460 [https://www.ncbi.nlm.nih.gov/geo/query/acc.cgi?acc=GSE316460].

Figures 1–8 and Supplementary Figures 1–9 have associated source data, including unprocessed immunoblot images, which are provided in the Source Data file.

Research involving human participants, their data, or biological material

Policy information about studies with human participants or human data. See also policy information about sex, gender (identity/presentation), and sexual orientation and race, ethnicity and racism.

Reporting on sex and gender	Human subjects were not used in this study.
Reporting on race, ethnicity, or other socially relevant groupings	N/A
Population characteristics	N/A
Recruitment	N/A
Ethics oversight	N/A

Note that full information on the approval of the study protocol must also be provided in the manuscript.

Field-specific reporting

Please select the one below that is the best fit for your research. If you are not sure, read the appropriate sections before making your selection.

☒ Life sciences ☐ Behavioural & social sciences ☐ Ecological, evolutionary & environmental sciences

For a reference copy of the document with all sections, see nature.com/documents/nr-reporting-summary-flat.pdf

Life sciences study design

All studies must disclose on these points even when the disclosure is negative.

Sample size	No formal sample size calculations were performed. Sample sizes were determined based on prior experience with similar measurements and common practice in the field. For imaging experiments, 50–100 cells were analysed per condition. For immunoblotting and other assays, experiments were performed with more than three independent biological replicates.
Data exclusions	No data were excluded from the analyses.
Replication	We tested our hypothesis using multiple complementary approaches, where feasible, to enhance scientific rigor and reproducibility. All experiments were independently replicated at least three times.
Randomization	Sample allocation was random. For imaging experiments, control samples were measured first, followed by patient samples, with all measurements performed on the same day for each replicate. For other experiments, control and patient samples were processed and measured together.
Blinding	Analyses were not blinded, as experiments were performed and analysed by the same researchers.

Reporting for specific materials, systems and methods

We require information from authors about some types of materials, experimental systems and methods used in many studies. Here, indicate whether each material, system or method listed is relevant to your study. If you are not sure if a list item applies to your research, read the appropriate section before selecting a response.

Materials & experimental systems

n/a	Involved in the study
☐	☒ Antibodies
☐	☒ Eukaryotic cell lines
☒	☐ Palaeontology and archaeology
☒	☐ Animals and other organisms
☒	☐ Clinical data
☒	☐ Dual use research of concern
☒	☐ Plants

Methods

n/a	Involved in the study
☒	☐ ChIP-seq
☒	☐ Flow cytometry
☒	☐ MRI-based neuroimaging

Antibodies

Antibodies used

All primary antibodies have anti-human protein reactivity:
Rabbit pAb anti-EPG5 (1:1000) Abcam Cat# ab122186
Mouse mAb anti-β-actin (1:10000) Cell Signaling Technology Cat# 3700
Mouse Ab anti-OxPhos cocktail (1:1000) Invitrogen Cat# 45-8199
Rabbit mAb anti-TOM20 (1:5000 IB, 1:200 IF) Abcam Cat# ab186735
Mouse mAb anti-MCU (1:1000) Sigma-Aldrich Cat# AMAB91189
Rabbit pAb anti-MCUB (1:1000) Proteintech Cat# 20387-1-AP
Rabbit pAb anti-MICU1 (1:1000) Thermo Scientific Cat# HPA037480
Rabbit pAb anti-MICU2 (1:1000) Sigma-Aldrich Cat# HPA045511
Rabbit pAb anti-MICU3 (1:1000) Thermo Scientific Cat# PA5-55177
Rabbit pAb anti-EMRE (1:1000) Abcam Cat# ab157387
Rabbit pAb anti-NCLX (1:1000) Sigma-Aldrich Cat# SAB2102181
Mouse mAb anti-ATP5A (1:1000) Abcam Cat# ab14748
Rabbit pAb anti-phospho PDH E1α (Ser293) (1:1000) Sigma-Aldrich Cat# AP1062
Mouse mAb anti- PDH E1α (1:5000) Abcam Cat# ab110330
Rabbit mAb anti-HA (1:1000) Abcam Cat# ab236632
Rabbit pAb anti-STAT1 (1:1000) Cell Signaling Technology Cat# 9172S
Rabbit mAb anti-phospho STAT1 (Tyr701) (1:1000) Cell Signaling Technology Cat# 9167S
Rabbit mAb anti-TBK1 (1:1000) Cell Signaling Technology Cat# 3504S
Rabbit mAb anti-phospho TBK1 (Ser172) (1:1000) Cell Signaling Technology Cat# 5483S
Rabbit mAb anti-STING (1:1000) Cell Signaling Technology Cat# 13647S
Rabbit pAb anti-IRF3 (1:1000) Abcam Cat# ab25950
Rabbit mAb anti-phospho IRF3 (Ser396) (1:1000) Cell Signaling Technology Cat# 29047S
Rabbit mAb anti-cGAS (1:100 IF) Cell Technology Cat# 15102S
Rabbit pAb anti-Citrate synthetase (1:100 IF) Abcam Cat# ab96600
Mouse mAb anti-DNA (1:200 IF) Sigma-Aldrich Cat# CBL186
Mouse mAb anti-Cytochrome C (1:200 IF) BD Pharmingen Cat# 556432
Mouse mAb anti-Beta-Tubulin III (TUJ1) (1:100 IF) STEMCELL Technologies Cat# 60052
Rabbit pAb anti-GFAP (1:100 IF) Sigma-Aldrich Cat# AB5804
Chicken pAb anti-MAP2 (1:100 IF) Abcam Cat# ab92434

Secondary antibodies:
HRP-Goat Anti-Rabbit IgG (H+L) (1:5000), Jackson ImmunoResearch Cat# 111-035-045
HRP-Goat Anti-Mouse IgG (H+L) (1:5000), Jackson ImmunoResearch Cat# 315-035-045
Alexa Fluor 647 Donkey anti-Rabbit IgG (H+L) (1: 1000), Thermo Scientific Cat# A-31573
Alexa Fluor 488 Donkey anti-Mouse IgG (H+L) (1: 1000), Thermo Scientific Cat# A-21202
Alexa Fluor 568 Donkey anti-Rabbit IgG (H+L)(1: 1000), Thermo Scientific Cat# A-10042
Alexa Fluor 488 Goat anti-Rabbit IgG (H+L)(1: 1000), Thermo Scientific Cat# A-11008
Alexa Fluor 568 Goat anti-Mouse IgG (H+L) (1: 1000), Thermo Scientific Cat# A-11004
Alexa Fluor 647 Goat anti-Chicken IgG (H+L) (1: 1000), Thermo Scientific Cat# A-21449

Validation

All antibodies used were purchased from commercial sources (e.g., Abcam, Cell Signaling Technology). Antibodies were selected based on reported reactivity with human proteins and on validation data provided by manufacturers and the published literature. In several cases, additional validation was performed by assessing the expected molecular weight by Western blotting and by knockdown of the protein of interest. Antibodies were aliquoted and stored according to the manufacturers' recommendations to minimise freeze–thaw cycles and associated loss of performance.

Eukaryotic cell lines

Policy information about cell lines and Sex and Gender in Research

Cell line source(s)

Human dermal fibroblasts from healthy control subjects were obtained from the MRC CNMD Biobank (London, UK). Primary skin fibroblasts from patients carrying pathogenic mutations in the EPG5 gene were isolated as part of the routine diagnostic process. BJ-hTERT fibroblasts were obtained from the American Type Culture Collection (ATCC; RRID: CVCL_6573). Induced pluripotent stem cell (iPSC) lines were purchased from the Coriell Institute (RRIDs: GM28930 and GM27291).

Authentication	BJ-hTERT fibroblasts and iPSC lines were authenticated by the original suppliers. Control and patient-derived fibroblasts were not independently authenticated.
Mycoplasma contamination	All cell lines tested negative for mycoplasma.
Commonly misidentified lines (See ICLAC register)	No commonly misidentified cell lines were used in this study.

Plants

Seed stocks	N/A
Novel plant genotypes	N/A
Authentication	N/A
