# Supplementary material for: Pathogenic variants in the autophagy-tethering factor EPG5 drive neurodegeneration through mitochondrial dysfunction and innate immune activation
Source: Nat Commun. 2026 May 26;17:6887. doi: 10.1038/s41467-026-73538-7 (PMC13388713; doi:10.1038/s41467-026-73538-7)
Supplement: Supplementary file 7 — Transparent Peer Review file [file 41467_2026_73538_MOESM7_ESM.pdf]

nature portfolio

Peer Review File

Pathogenic variants in the autophagy-tethering factor EPG5 drive neurodegeneration through mitochondrial dysfunction and innate immune activation

Corresponding Author: Professor Michael Duchen

Parts of this Peer Review File have been redacted as indicated to maintain the confidentiality of unpublished data.

This file contains all reviewer reports in order by version, followed by all author rebuttals in order by version.

Version 0:

Reviewer comments:

Reviewer #1

(Remarks to the Author)
In this study, Singh et al. demonstrated that in human dermal fibroblasts derived from patients with pathogenic EPG5 mutations and in iPSC-derived cortical neurons, aberrant downregulation of MICU1/3 leads to mitochondrial Ca^{2+} dysregulation, which subsequently impairs mitochondrial bioenergetics. Mitochondrial Ca^{2+} overload caused by pathogenic EPG5 mutations triggers mtDNA release, activating the cGAS-STING pathway and interferon response, a process mediated by mPTP opening. Pharmacological inhibition of mPTP significantly rescues mitochondrial bioenergetic deficits and attenuates STING activation. This study advances our understanding of mitochondrial pathology in EPG5-related disorders and identifies promising therapeutic targets. Several key questions need be addressed to strengthen their conclusions:

1. The authors proposed that impaired mitophagy contributes to mitochondrial bioenergetic dysfunction in patient-derived fibroblasts. Direct evidence for this link should be provided. Does impaired mitophagy in other autophagy deficient cells cause the same defect as pathogenic EPG5 mutations?

2. The mechanism by which pathogenic EPG5 mutations lead to downregulation of MICU1/3 should be investigated.

3. The authors need check whether wild type EPG5 rescues the mitochondrial defects observed in fibroblasts derived from patients with pathogenic EPG5 mutations or Q336R iPSC-derived neurons.

4. Fig. 1A-B, EPG5 protein is almost complete absence in all patient cells. Is mutant EPG5 protein degraded by proteasome or lysosomes?

5. EPG5 deletion and EPG5 missense mutations cause a distinct spectrum of neurodevelopment phenotype. Does the mitochondria-related defect examined in this study exhibit differences in cells with EPG5 deletion and missense mutations?

Reviewer #2

(Remarks to the Author)
The paper by Singh et al. is an interesting and very detailed study on the role of EPG5, a factor involved in autophagy, and the mitochondrial mechanisms involved in the disease caused by mutations in EPG5. Autosomal recessive mutations in EPG5 result in impaired autophagy causing a rare devastating multisystem disorder known as Vici syndrome, leading to neurodevelopmental defects, severe progressive neurodegeneration and immunodeficiency.
In patient-derived fibroblasts and iPSC-derived cortical neurons, the authors detected mitochondrial dysfunction with impaired mitophagy, mitochondrial Ca2+ overload due to downregulation of MICU1/3, mitochondrial depolarisation, mtDNA release and activation of the cGAS-STING pathway. Some of these changes were rescued by administration of inhibitors of the mitochondrial permeability transition pore (mPTP) or of the STING pathway.
It is a very detailed and nicely done study, but I have a few questions to the authors to clarify in the manuscript.

1. How much of the EPG5 disease is caused by the mitochondrial impairments? Does the gene defect result in other effects via altered autophagy which may be relevant to cause disease?
2. How general is the statement that "increased resting [Ca2+]m in patient cells can cause chronic mtDNA release? Has mtDNA release be reported in other mitochondrial diseases with abnormal mitochondrial calcium metabolism?
3. What is the reason behind that the cGAS/STING inhibition have not rescued the mitochondrial RC complex defect? Please explain more clearly.
4. Would JP1-138 treatment be feasible for primary mitochondrial diseases with abnormal calcium metabolism?
5. "Disruption of mitochondrial Ca2+ homeostasis is the key contributing factor during glutamate excitotoxicity" - Is the impaired calcium homeostasis linked to glutamate?
6. Is the mitochondrial pathology the primary pathomechanism in EPG5? Please, discuss it more precisely.
7. Does mitochondrial Ca2+ overload act mostly via increased vulnerability to mPTP opening and mtDNA release leading to cGAS/STING dependent IFN response?
8. What are the differences in the responses between EPG5 mutant fibroblasts and iPSC derived neurons? Are these mechanisms tissue specific? It would be nice to specify it.
9. It is a bit unclear what is the difference in these mechanisms in missense and truncating mutations.
10. How can defective mitophagy and mtDNA release explain the lack of influenza cases in patients with EPG5-RD?

Reviewer #4

(Remarks to the Author)
Kritarth Singh, et al. show that multiple mitochondrial functions are changed in EPG5 mutant patient cells, such as mitochondrial membrane potential, mitochondrial morphology, impaired mitochondrial bioenergetics, mitochondrial Ca2+ overload, vulnerability to mPTP opening and activation of mtDNA-cGAS-STING-IFN pathway. The authors propose that these events contribute to EPG5 pathogenesis. However, the conclusions are not well supported by the data presented. Below are some concerns about the manuscript.

Major

1. EPG5 is a tethering protein that facilitates fusion between autophagosomes and lysosomes, yet the authors only investigated its role in mitochondrial function. What is the mechanism by which EPG5, a protein primarily acting in another organelle system, regulates mitochondrial function? This is something the authors need to elucidate.

2. A homozygous or heterozygous missense mutation affecting a single amino acid (such as Q336R or R1621G) can lead to undetectable protein expression and a reduction in EPG5 mRNA levels. What is the mechanism behind this phenomenon? In cells, if one overexpresses wild-type and mutant EPG5 (Q336R, R1621G), will the mutant EPG5 fail to be expressed?

3. The entire study investigates the effects of EPG5 deficiency on mitochondria using patient cells. However, given the small sample size (n=4) and individual variability between cells, and since the paper focuses on EPG5, the authors should generate EPG5-deficient (knockdown or knockout) cells in a healthy cell line, or rescue patient cells with EPG5 to rule out patient genetic background effects. This would allow them to compare the effects on mitochondria in the absence of EPG5 versus after its reintroduction. Specifically, they should examine mitochondrial calcium dynamics and the cGAS-STING-IFN response. This approach would better align with the theme of the paper and highlight the role of EPG5 in mitochondrial regulation.

4. For the mitochondrial Ca^{2+} part, mitochondrial Ca^{2+} levels are higher in patient cells, but Calcium Green-5N data shows that mitochondrial calcium buffering is lower, making them more vulnerable to mitochondrial Ca^{2+} overload and mPTP opening. How can this be explained?

5. In lines 264-266, the authors mention that they aim to investigate the role of MCU proteins in calcium regulation. However, they did not perform overexpression or further knockdown of MCU proteins to assess this. Instead, they used a CypD inhibitor.

6. Throughout the entire paragraph from lines 263–276, the authors did not manipulate MICU1 or MICU2 expression, yet they drew conclusions about MICU1 and MICU2. To support these conclusions, the authors need to overexpress MICU proteins in patient cells.

7. It is well established that silencing MICU1 abolish mitochondrial calcium entry. How to explain the reduced MICU1, MICU2 accompanied by increased mitochondrial Ca2+ in this study.

8. In Figure 3, the authors need to block or reduce mitochondrial Ca^{2+} to assess cytosolic mtDNA release. This is necessary to demonstrate that the release of cytosolic mtDNA and downstream activation of cGAS-STING are due to mitochondrial calcium overload.

9. Figure 4H, and S4G-K, the patient cells have increased mitochondrial calcium, so authors should reduce mitochondrial calcium or block mitochondrial calcium, not using TG or histamine to further enhance mitochondrial calcium. The TG or histamine induced mitochondrial calcium overload is artificial. Authors should reduce mitochondrial calcium to detect cGAS-STING pathway in patient cells or EPG5 KO/KD cells.

10. Again, in Figure 5, the authors only inhibited mPTP but did not reduce mitochondrial Ca^{2+} levels. They need to lower mitochondrial Ca^{2+} to assess the STING-dependent IFN response. Additionally, when mitochondrial calcium is further increased to induce overload, inhibition of mPTP followed by measurement of the cGAS-STING-IFN response is necessary. This approach is required to clearly demonstrate the effect of mitochondrial calcium on the STING-IFN pathway.

11. Figures 6-8: The role of mtDNA release from mPTP opening in triggering the cGAS-STING-IFN response is well established. Since JPI-138 is a CypD inhibitor, its effect in improving the cGAS-STING-IFN pathway is predictable. The authors should examine the mitochondrial calcium-mtDNA-cGAS-STING-IFN axis in EPG5 knockout or rescue cells. In addition to patient cells, mPTP inhibitor effects on this pathway should also be tested in EPG5 KO/KD cells.

Minor

1. In Abstract, line 43, downregulation of MICU1/2. Authors only detected MICU1 and MICU2 proteins in Figure 2H.
2. The reporter used to detect NADH is not clearly described.
3. The molecular weight of ATP5A is approximately 55 kDa, but in the paper it appears below 37 kDa (Fig. 2H, 7F).
4. The method for detecting mitochondrial complexes on a single blot in Figure 1K needs to be clearly explained.
5. Figures 2F and 2D are inconsistent. According to the legend in Figure 2F, the “time when mitochondrial Ca^{2+} rise was at half-maximal” is described, but the figure actually shows the time when mitochondrial Ca^{2+} rise was at the maximum.
6. In Figure 4I-J, * labeling should be patient vs ctrl, not patient vs patient
7. In Figure 4J, p-STAT1/STAT1 analysis doesn’t match WB results in Figure 4H.
8. Missing protein molecular weight in Figure 5F and Figure S5A
9. All WB should be on the same membrane in Figure S5A.

Version 1:

Reviewer comments:

Reviewer #1

(Remarks to the Author)
The authors have largely resolved the issues raised in the previous round of review. However, there is one remaining point that merits further clarification to enhance the depth of the manuscript:

While defects in mitophagy are a common feature of various autophagy-related gene mutations, the pathological phenotypes associated with EPG5 differ significantly from those of other autophagy genes. This observation suggests that impaired mitophagy may only be a partial contributor to the EPG5 pathology, implying the existence of other underlying mechanisms. I recommend that the authors briefly address this discrepancy in the Discussion section and speculate on potential alternative mechanisms that might drive the specific phenotype of EPG5 deficiency.

Reviewer #2

(Remarks to the Author)
The authors answered my questions and imporved the manuscript. I don`t have any more questions.

Reviewer #4

(Remarks to the Author)
The authors have adequately addressed this reviewer's concerns.

Open Access This Peer Review File is licensed under a Creative Commons Attribution 4.0 International License, which permits use, sharing, adaptation, distribution and reproduction in any medium or format, as long as you give appropriate credit to the original author(s) and the source, provide a link to the Creative Commons license, and indicate if changes were made.
In cases where reviewers are anonymous, credit should be given to 'Anonymous Referee' and the source.
The images or other third party material in this Peer Review File are included in the article's Creative Commons license, unless indicated otherwise in a credit line to the material. If material is not included in the article's Creative Commons license and your intended use is not permitted by statutory regulation or exceeds the permitted use, you will need to obtain permission directly from the copyright holder.
To view a copy of this license, visit https://creativecommons.org/licenses/by/4.0/

REVIEWER COMMENTS

Reviewer #1 (Remarks to the Author):

In this study, Singh et al. demonstrated that in human dermal fibroblasts derived from patients with pathogenic EPG5 mutations and in iPSC-derived cortical neurons, aberrant downregulation of MICU1/3 leads to mitochondrial Ca^{2+} dysregulation, which subsequently impairs mitochondrial bioenergetics. Mitochondrial Ca^{2+} overload caused by pathogenic EPG5 mutations triggers mtDNA release, activating the cGAS-STING pathway and interferon response, a process mediated by mPTP opening. Pharmacological inhibition of mPTP significantly rescues mitochondrial bioenergetic deficits and attenuates STING activation. This study advances our understanding of mitochondrial pathology in EPG5-related disorders and identifies promising therapeutic targets. Several key questions need be addressed to strengthen their conclusions:

We thank the Reviewer for their positive feedback, and thoughtful suggestions. We have addressed all the comments and have carefully revised the manuscript, as detailed below and hope that these changes have strengthened the study.

Reviewer:
1. The authors proposed that impaired mitophagy contributes to mitochondrial bioenergetic dysfunction in patient-derived fibroblasts. Direct evidence for this link should be provided. Does impaired mitophagy in other autophagy deficient cells cause the same defect as pathogenic EPG5 mutations?

Authors:
i) We have provided comprehensive ***direct evidence*** for a link in our recent article published in *Annals of Neurology* (Dafsari et al., 2025), in which we have demonstrated the consequence of the primary autophagy defect on the mitochondrial quality control pathways in EPG5-deficient patient fibroblasts carrying Q336R and R1621G mutations and in mouse embryonic fibroblasts (MEFs) from Epg5 knock-in mice carrying the Q331R mutation, homologous to the human EPG5 Q336R variant.

We showed that EPG5 deficiency causes a pronounced autophagosome–lysosome fusion defect (Dafsari et al., 2025: Fig. 6A–D, Supplementary Fig. 7A–B), interrupting mitophagy flux and impaired mitochondrial clearance, thereby permitting the accumulation of dysfunctional mitochondria. Assessment of mitophagy flux by immunoblotting (Dafsari et al., 2025: Fig. 6E–

F), together with live-cell imaging of patient fibroblasts expressing the quantitative mitophagy reporter mt-Keima, revealed a marked impairment in PINK1/Parkin-dependent mitophagy (Dafsari et al., 2025: Fig. 6G–M).

In Q331R MEFs, quantitative analysis further demonstrated an accumulation of enlarged, swollen mitophagosomes by transmission electron microscopy (Dafsari et al., 2025: Fig. 4J–L), while flow cytometry showed a severely reduced rate of Parkin-dependent delivery of damaged mitochondria to lysosomes compared with wild-type MEFs (Dafsari et al., 2025: Fig. 4M–N). Consistent with the Q336R and R1621G patient fibroblasts, measurements of mitochondrial oxygen consumption in Q331R MEFs revealed a significant reduction in both ATP-linked respiration and spare respiratory capacity relative to wild-type controls (Dafsari et al., 2025: Fig. 4O–Q).

These data provide direct evidence that EPG5 deficiency leads to mitochondrial dysfunction through a failure of autophagic removal of damaged mitochondria, thereby allowing the progressive accumulation of mitochondrial defects in patient-derived cells.

ii) To the question whether impaired mitophagy in other autophagy deficient cells cause the same defect as pathogenic EPG5 mutations, previous studies using mouse models with primary disruptions in the autophagy pathway have indeed demonstrated that the accumulation of dysfunctional mitochondria compromises mitochondrial function and homeostasis.

For example, macrophages deficient in Beclin 1 (*Becn1*$^{+/-}$) or LC3B (*Map1lc3b*$^{-/-}$), autophagic proteins required for the early and late stages of autophagosome formation, exhibit accumulation of swollen mitochondria with severely disrupted cristae and increased mitochondrial ROS production (Nakahira et al., 2011). Similarly, macrophage-specific ablation of *Sqstm1/p62*, a key autophagy receptor, impairs Parkin-dependent mitophagy and leads to the accumulation of damaged mitochondria (Zhong et al., 2016). Notably, loss of these autophagy-related proteins further results in activation of NLRP3-dependent inflammatory signaling and increased IL-1β secretion in macrophages.

In addition, Aoyagi *et al.* showed that pancreatic β-cell–specific knockout of VAMP7, a SNARE protein mediating autophagosome–lysosome fusion, causes the accumulation of dysfunctional mitochondria and impairs glucose-stimulated ATP production (Aoyagi et al.,

2016). More recently, *Atg16l1*-deficient mice were shown to exhibit defective mitochondrial turnover and impaired memory CD8＋ T cell differentiation (Borsa et al., 2025).

We have summarized these findings and incorporated them into the Discussion section of the revised manuscript (lines: 946-948).

Reviewer:
2. The mechanism by which pathogenic EPG5 mutations lead to downregulation of MICU1/3 should be investigated.

Authors:
This is an interesting point that we have naturally pondered. We have a small body of work addressing this question. We feel that on balance this is effectively a new study, and that this work is beyond the scope of the present paper, which is already quite involved and complex. We reprise these data below for the reviewer, but we ask that this section is redacted from the reviewer report if published.

Proteostasis stress arising from impaired mitophagy impacts local mitochondrial quality control regulated by mitochondrial proteases involved in IMM proteome remodelling and induction of the mitochondrial unfolded protein response (Chakrabarty et al., 2024; Uoselis et al., 2023; Yamada et al., 2025). Our findings suggest that impaired mitochondrial turnover of EPG5-deficient patient fibroblasts triggers activation of this stress response. Specifically, we observed induction of the integrated stress response (ISR), characterised by increased expression of nuclear-encoded transcription factors such as ATF4 and CHOP, along with upregulation of mitochondrial chaperones and IMM proteases, including OMA1, YME1L, and AFG3L2, which collectively act to mitigate mitochondrial proteotoxic stress.

Immunoblot analyses confirmed ISR activation in patient fibroblasts, as evidenced by altered expression of IMM proteases and downstream ISR markers (Figure 1A-D below). Elevated levels of the processed short isoform of OPA1, together with reduced levels of full-length OMA1, were consistent with stress-induced OMA1 activation followed by autocatalytic degradation (Figure 1A-B below). Notably, YME1L expression was increased in patient fibroblasts, and elevated OMA1 activity strongly correlated with CHOP-mediated ISR activation (Figure 1C-D below).

Previous studies have suggested that OMA1 and YME1L may directly regulate the proteolytic turnover of MICU1 and MICU2 under conditions of mitochondrial stress (Acin-Perez et al.,

2018; Tsai et al., 2022). To test this possibility, we examined MICU1 abundance following siRNA-mediated knockdown of YME1L in patient fibroblasts. Reduction of YME1L expression restored MICU1 protein levels, supporting a role for YME1L-mediated proteolysis in MICU1 downregulation (Figure 1E-D below).

Together, these findings support a model in which increased YME1L-mediated proteolysis during mitochondrial stress contributes to MICU1 downregulation in EPG5-deficient fibroblasts. At the same time, they underscore the complex and interconnected nature of mitochondrial quality control pathways, as well as the diverse and independent roles of IMM proteases in maintaining mitochondrial homeostasis. **We have briefly discussed this plausible mechanism in the Discussion section of the revised manuscript (lines 811–820). We hope to further address this important question in future work and therefore request that the reviewer and editor allow us to exclude these data from the current submission.**

(Redaction)

Reviewer:

3. The authors need check whether wild type EPG5 rescues the mitochondrial defects observed in fibroblasts derived from patients with pathogenic EPG5 mutations or Q336R iPSC-derived neurons.

Authors:

We agree that this is an important experiment. We have partially addressed this by utilizing an isogenic iPSC derived neuronal control line in which the *EPG5* c.1007A>G (p.Q336R) mutation was corrected using CRISPR/Cas9, thereby restoring wild-type *EPG5* expression. As shown in **Fig. 7 and 8 of this manuscript**, isogenic cortical neurons exhibit normal changes in $[Ca^{2+}]_c$ in response to 10 µM glutamate stimulation and a cooperative sigmoid regulation of $[Ca^{2+}]_m$ uptake. Importantly, these neurons maintained $\Delta\Psi_m$ under glutamate excitation and displayed normal ATP-linked respiration and spare respiratory capacity.

To assess whether wild-type EPG5 suppresses mtDNA release and mitigates STING-dependent type-I IFN signaling, we performed siRNA-mediated knockdown of *EPG5* in hTERT-immortalized skin fibroblasts, followed by ectopic expression of EPG5-GFP. These cell

lines were subsequently analysed for cytosolic mtDNA release and activation of STING-dependent type-I IFN signaling. Airyscan imaging of EPG5-siRNA cells immunolabeled with TOM20 and DNA revealed a marked increase in cytosolic DNA puncta, as found in the cells carrying EPG5 mutations **(Supplementary Fig. 7b)**. In contrast, ectopic expression of EPG5 significantly reduced both the average number and the percentage of cells with cytosolic DNA puncta **(Supplementary Fig. 7c, d)**.

Consistent with these findings, immunoblot analysis showed that EPG5 re-expression was sufficient to suppress TBK1 activation, reduce downstream IRF3 and STAT1 phosphorylation, and restore STING expression levels to those observed in control cells **(Fig. 5i-k and Supplementary Fig. 6g, h)**. Furthermore, the mRNA expression levels of ISGs were also significantly reduced in EPG5-siRNA cells expressing EPG5-GFP **(Fig. 5l, m and Supplementary Fig. 6i-k)**.

Together, these data confirm that loss of EPG5 function impairs mitochondrial bioenergetic capacity and disrupts mitochondrial Ca^{2+} signaling, thus increasing neuronal vulnerability to excitotoxic cell death and promoting mtDNA release and STING-dependent IFN responses in patient skin fibroblasts. **These findings have now been incorporated into the Results section of the revised manuscript (lines: 697-728 and 732-741) and are presented in the updated Figure 5 and 6.**

Reviewer:

4. Fig. 1A-B, EPG5 protein is almost complete absence in all patient cells. Is mutant EPG5 protein degraded by proteasome or lysosomes?

Authors:

Both transcriptional and post-translational mechanisms influence the overall abundance and stability of mutant EPG5 protein in patient-derived fibroblasts carrying missense and truncating variants. A previous report (Kane et al., 2016), along with recent findings from our group (manuscript submitted), confirms that the Q336R missense variant affects pre-mRNA splicing, leading to the generation of multiple aberrant splice isoforms. These aberrant isoforms account for >80% of residual transcripts, most of which harbor a premature termination codon and are subsequently degraded by nonsense-mediated mRNA decay. Similarly, R1631Q, another homozygous founder missense variant in EPG5, disrupts mRNA splicing and produces an aberrant isoform with an in-frame deletion of 18 amino acids, with complete absence of the wild-type isoform (Vansenne et al., 2022). **Together, these findings**

indicate that Q336R and R1631Q mutations create null alleles through defective mRNA splicing.

At the protein level, EPG5 turnover appears to be predominantly regulated by lysosomal degradation, as previously demonstrated by Gu et al. in embryonic stem cells expressing wild-type EPG5 (Gu et al., 2019). To answer whether degradation of mutant EPG5 protein is mediated by the ubiquitin–proteasome system (UPS) or the autophagy–lysosomal pathway, **we include the data below for the reviewer's consideration and have summarized these findings in the Discussion section of the revised manuscript (lines: 974-983).**

The stability of the Q336R EPG5 variant in patient-derived fibroblasts was assessed following inhibition of protein translation with cycloheximide, combined with analyses under conditions of nutrient deprivation, pharmacological inhibition of the autophagy–lysosomal pathway, or UPS inhibition. Mutant EPG5 degradation was monitored by immunoblotting (Figure 3A-B below). Surprisingly, we did not observe an accumulation of mutant EPG5 following autophagy–lysosomal inhibition with bafilomycin A1, in contrast to wild-type EPG5 controls. This lack of accumulation is likely explained by the very low levels of residual *EPG5* transcript available for translation and/or the rapid degradation kinetics of the mutant EPG5 protein in patient-derived fibroblasts.

(Redaction)

Reviewer:

5. EPG5 deletion and EPG5 missense mutations cause a distinct spectrum of neurodevelopment phenotype. Does the mitochondria-related defect examined in this study exhibit differences in cells with EPG5 deletion and missense mutations?

Authors:

The neurodevelopmental phenotype is observed in all patients with pathogenic *EPG5* variants, including both truncating and missense mutations. Truncating (deletion) variants are typically associated with additional multisystem features, such as cataracts or immunodeficiency. In our primary fibroblast model derived from patients with classic Vici syndrome (patients 3 and 4) and those with an attenuated neurodevelopmental phenotype (patients 1 and 2), impaired mitophagy flux and mitochondrial dysfunction appear to be common pathophysiological mechanisms driving chronic activation of inflammatory signaling. However, in terminally differentiated and bioenergetically vulnerable iPSC-derived cortical neurons, compromised mitochondrial function sensitizes neurons to excitotoxicity and cell death. Further studies will be required to determine the extent to which impaired mitochondrial bioenergetic function contributes to the pathophysiology of other post-mitotic cell types and shapes the multisystem phenotype in patients carrying truncating and missense *EPG5* variants.

Reviewer #2 (Remarks to the Author):

The paper by Singh et al. is an interesting and very detailed study on the role of EPG5, a factor involved in autophagy, and the mitochondrial mechanisms involved in the disease caused by mutations in EPG5. Autosomal recessive mutations in EPG5 result in impaired autophagy causing a rare devastating multisystem disorder known as Vici syndrome, leading to neurodevelopmental defects, severe progressive neurodegeneration and immunodeficiency. In patient-derived fibroblasts and iPSC-derived cortical neurons, the authors detected mitochondrial dysfunction with impaired mitophagy, mitochondrial Ca2+ overload due to downregulation of MICU1/3, mitochondrial depolarisation, mtDNA release and activation of the cGAS-STING pathway. Some of these changes were rescued by administration of inhibitors of the mitochondrial permeability transition pore (mPTP) or of the STING pathway. It is a very detailed and nicely done study, but I have a few questions to the authors to clarify in the manuscript.

We thank the reviewer for their enthusiastic feedback and for appreciating the depth and scope of our study. We have carefully addressed all of the reviewer's questions and comments in the revised manuscript, as detailed below.

Reviewer:
1. How much of the EPG5 disease is caused by the mitochondrial impairments? Does the gene defect result in other effects via altered autophagy which may be relevant to cause disease?

Authors:
This is an important point, and we have now summarised our response to both questions and incorporated it into the Discussion section of the revised manuscript (lines: 1143-1169).

Our findings from the current study, together with our recently published work in *Annals of Neurology* (Dafsari et al., 2025), demonstrate that impaired mitophagy directly leads to mitochondrial dysfunction, which in turn contributes to inflammation and neuronal cell death. Defective mitochondrial function and dysregulated mitochondrial calcium signalling are likely to represent core pathogenic mechanisms in post-mitotic, energetically demanding cell types such as neurons and cardiomyocytes. These defects may therefore be major contributors to the neurodegeneration, epilepsy and (cardio)myopathy characteristic of *EPG5*-related disorders.

However, the clinical manifestations associated with EPG5 deficiency span a broader phenotypic spectrum that is unlikely to be explained solely by impaired mitophagy. Previous work from our group has shown that secondary defects in selective autophagy pathways, such as nucleophagy, lead to compromised nuclear integrity, cytosolic accumulation of LaminB1, and progressive neuronal degeneration (Baron et al., 2017). Additionally, we have reported that EPG5 deficiency with truncating variants increases susceptibility to severe bacterial infections through a Rab7-mediated impairment of Toll-Like Receptor signalling (Piano Mortari et al., 2018). Other studies have further demonstrated that EPG5 regulates early endosome sorting and maturation by modulating RAB protein dynamics, and its loss results in endocytic trafficking defects and the accumulation of non-degradative autophagic intermediates (Yuan et al., 2025). Taken together, these findings highlight that both mitochondrial dysfunction and broader autophagy-related defects contribute to the complex pathophysiology of *EPG5*-related disease.

Reviewer:

2. How general is the statement that "increased resting [Ca2+]m in patient cells can cause chronic mtDNA release? Has mtDNA release be reported in other mitochondrial diseases with abnormal mitochondrial calcium metabolism?

Authors:

We have addressed this question in detail below and have also summarised the key points in the Discussion section of the revised manuscript (lines: 1050-1067).

Emerging evidence indicates that chronic mitochondrial stress can promote the release of mtDNA from a limited subset of mitochondria, leading to cGAS–STING–mediated induction of type I interferon (IFN) signaling and inflammation without triggering overt cell death (Kim et al., 2019; Victorelli et al., 2023). Although chronic mtDNA release associated with increased mitochondrial Ca^{2+} influx and mPTP opening has been implicated in pathological contexts such as atherosclerosis (Xian et al., 2022), autoimmune diseases (Kim et al., 2019), cellular senescence (Victorelli et al., 2023), and autophagy defects (Nakahira et al., 2011), a direct mechanistic link between dysregulated mitochondrial Ca^{2+} signaling and mtDNA release has remained poorly defined.

Our study specifically addresses this gap by delineating early pathological events in EPG5-deficient cells. We demonstrate that unregulated mitochondrial Ca^{2+} uptake and overload compromise inner mitochondrial membrane integrity by sensitizing mitochondria to mPTP

opening, thereby enabling mtDNA release from a restricted subset of damaged mitochondria. This provides a mechanistic framework linking elevated resting $[Ca^{2+}]_m$ to chronic, low-grade mtDNA-driven inflammatory signaling.

To our knowledge, direct evidence linking mtDNA release to abnormal mitochondrial Ca^{2+} metabolism in primary mitochondrial diseases remains limited. However, mtDNA-mediated activation of type I IFN responses has been reported in animal models of chronic inflammatory diseases, such as atherosclerosis (Xian et al., 2022), as well as in cellular models of secondary mitochondrial dysfunction; these studies are now cited in the revised manuscript. Notably, Kim et al. reported mtDNA-dependent type I IFN production in MICU1-deficient mouse embryonic fibroblasts (Kim et al., 2019), which correlates with the interferon proteomic signature observed in lymphoblastoid cell lines derived from patients with MICU1 mutation (Kohlschmidt et al., 2021).

Reviewer:

3. What is the reason behind that the cGAS/STING inhibition have not rescued the mitochondrial RC complex defect? Please explain more clearly.

Authors:

We apologise for any ambiguity regarding this point and have clarified it in the Discussion section of the revised manuscript as detailed below (lines: 1171-1178).

We tested pharmacological inhibition of the cGAS–STING pathway to determine whether innate immune signalling feeds back to exacerbate mitochondrial respiratory chain dysfunction in EPG5-deficient cells. While inhibition of cGAS–STING signalling effectively suppressed downstream type I IFN responses, it did not restore mitochondrial bioenergetic function.

These findings indicate that activation of the cGAS–STING pathway occurs downstream of mitochondrial dysfunction rather than acting as a driver of the mitochondrial bioenergetic defect. In other words, impaired mitochondrial respiration and Ca^{2+} dysregulation precede mtDNA release and cGAS–STING activation, and suppression of inflammatory signalling alone is insufficient to reverse the underlying mitochondrial defects

Reviewer:

4. Would JP1-138 treatment be feasible for primary mitochondrial diseases with abnormal calcium metabolism?

Authors:

At present, there is limited clinical rationale for targeting the mitochondrial permeability transition pore (mPTP) as a therapeutic strategy in primary mitochondrial diseases, largely due to the lack of direct evidence linking abnormal (excessive) calcium influx to disease pathogenesis. Nevertheless, previous studies have reported that mitochondrial Ca^{2+} overload and mPTP opening contribute to neuronal cell death in mouse models of spinocerebellar ataxia type 28 (SCA28) (Konig et al., 2016) and MICU1 deficiency (Singh et al., 2022), as well as in cellular models of inherited mitochondrial optic neuropathies (Whitehead et al., 2025). Given its favourable preclinical safety profile and improved pharmacokinetic properties, further preclinical evaluation of JP1-138 will be required to assess its long-term efficacy and safety in these subsets of primary mitochondrial diseases characterised by dysregulated mitochondrial Ca^{2+} homeostasis.

Reviewer:

5. “Disruption of mitochondrial Ca2+ homeostasis is the key contributing factor during glutamate excitotoxicity” - Is the impaired calcium homeostasis linked to glutamate?

Authors:

Indeed, mitochondrial calcium signalling plays a crucial role in shaping neuronal responses to glutamate (Plotegher et al., 2021). A substantial body of evidence demonstrates that, under physiological conditions, mitochondria support cellular bioenergetics, finely tune intracellular Ca^{2+} dynamics, and regulate glutamate availability by utilising it as a metabolic substrate (Please see Plotegher et al., 2021 for a comprehensive review).

However, when mitochondrial function is compromised as observed in neurodegenerative diseases, stroke, or traumatic brain injury, neurons become highly susceptible to excitotoxic stress. Under these conditions, even physiological concentrations of glutamate can trigger pathological signalling cascades, including excessive activation of neuronal nitric oxide synthase (nNOS), mitochondrial Ca^{2+} overload, and opening of the mitochondrial permeability transition pore (mPTP). Together, these events precipitate mitochondrial dysfunction and ultimately lead to neuronal cell death.

Reviewer:

6. Is the mitochondrial pathology the primary pathomechanism in EPG5? Please, discuss it more precisely.

Authors:

We have addressed this question in detail in our response to **Comment #1**. Briefly, while mitochondrial dysfunction represents a central and early pathogenic mechanism in EPG5-related disorders, it is unlikely to be the sole driver of disease pathology. **We have now summarised this discussion and incorporated it into the Discussion section of the revised manuscript (lines: 1143-1169).**

Reviewer:

7. Does mitochondrial Ca2+ overload act mostly via increased vulnerability to mPTP opening and mtDNA release leading to cGAS/STING dependent IFN response?

Authors:

Our data, together with previous studies, suggest that the cellular consequences of mitochondrial Ca^{2+} overload depend on both the (patho)physiological state of the cell and its underlying bioenergetic vulnerability to mitochondrial dysfunction. In neurons, which are intrinsically vulnerable due to high metabolic demand and physiological Ca^{2+} fluxes, mitochondrial Ca^{2+} overload sensitizes cells to excitotoxicity and cell death. In contrast, in bioenergetically competent primary fibroblasts, mitochondrial Ca^{2+} overload drives maladaptive inflammatory signaling through mtDNA release and activation of the cGAS–STING–IFN pathway.

Reviewer:

8. What are the differences in the responses between EPG5 mutant fibroblasts and iPSC derived neurons? Are these mechanisms tissue specific? It would be nice to specify it.

Authors:

We appreciate this important question. Our response to this comment overlaps with **Comment #7**, and **the key points have also been summarized in the Discussion section of the revised manuscript (lines: 962-972 and 1201-1208).**

Indeed, our data indicate that dysregulation of mitochondrial Ca^{2+} signaling leads to distinct pathophysiological outcomes in EPG5-mutant primary fibroblasts and iPSC-derived cortical neurons. In cortical neurons, which are intrinsically vulnerable due to their high metabolic demand and physiological activity, mitochondrial Ca^{2+} overload increases susceptibility to excitotoxicity and cell death. In contrast, in bioenergetically competent primary fibroblasts,

mitochondrial Ca^{2+} overload predominantly drives maladaptive inflammatory signaling through mtDNA release and activation of the cGAS–STING–type I IFN pathway.

Importantly, these findings suggest that mitochondrial dysfunction resulting from impaired quality control in EPG5 deficiency may exerts tissue-specific effects. In the brain, this may manifest as both cell-autonomous neuronal vulnerability and non–cell-autonomous inflammatory responses in metabolically competent cells, such as neuroglia, which together may contribute to and accelerate neurodegeneration in *EPG5*-related disorders.

Reviewer:

9. It is a bit unclear what is the difference in these mechanisms in missense and truncating mutations.

Authors:

Pathogenic *EPG5* variants, including both truncating and missense mutations, are consistently associated with a neurodevelopmental and neurodegenerative phenotypes in patients. Truncating variants are more frequently linked to additional multisystem manifestations, such as cataracts and immunodeficiency, whereas missense variants are often associated with a comparatively attenuated clinical presentation.

In our primary fibroblast models derived from patients with classic Vici syndrome (patients 3 and 4, carrying truncating variants) as well as from patients with an attenuated neurodevelopmental phenotype (patients 1 and 2, carrying missense variants), we observed impaired mitophagy flux and mitochondrial dysfunction as shared pathophysiological features. In both settings, these defects were associated with chronic activation of inflammatory signaling, indicating that disruption of mitochondrial quality control represents a common mechanism across different classes of EPG5 mutations.

In contrast, in terminally differentiated and bioenergetically vulnerable iPSC-derived cortical neurons, compromised mitochondrial function primarily sensitised cells to excitotoxicity and cell death. These findings suggest that while impaired mitophagy and mitochondrial dysfunction could be key mechanisms in EPG5 deficiency, the downstream cellular consequences are strongly influenced by cell type and metabolic state.

Further studies will be required to determine how differences in mitochondrial bioenergetic reserve and tissue-specific demands contribute to the broader multisystem phenotype observed in patients carrying truncating versus missense *EPG5* variants.

Reviewer:

10. How can defective mitophagy and mtDNA release explain the lack of influenza cases in patients with EPG5-RD?

Authors:

The absence of influenza infections in patients with *EPG5*-related disorders represents a striking clinical observation for which a clear mechanistic explanation has been lacking. Our findings provide a potential mechanism to understand this phenomenon, which is addressed below, and **the key points have also been summarized in the Discussion section of the revised manuscript (lines: 1089-1104).**

This observation is consistent with previous studies in Epg5-deficient mouse models, which demonstrated exaggerated pulmonary inflammation (Lu et al., 2016) as well as increased resistance to intestinal viral infections, including norovirus and rotavirus, accompanied by spontaneous intestinal inflammation in the absence of infectious triggers (Lee et al., 2022). These studies identified enhanced IFN and TNF signaling as a central driver of the hyperinflammatory phenotype; however, the upstream mechanism linking autophagic dysfunction to aberrant innate immune activation remained unclear.

More recently, transcriptomic analyses of *Epg5* missense and knockout mouse models revealed progressive neuroinflammation with enrichment of gene ontology terms related to viral pattern recognition (Thornton et al., 2025), suggesting chronic activation of innate immune pathways. Independent studies have shown that stress-induced mtDNA or mtRNA release to cytoplasm triggers IFN-I response that confers resistance to viral infection (Bahat et al., 2025; Hooftman et al., 2023; West et al., 2015).

Our data extend these findings by demonstrating that EPG5 deficiency leads to defective mitophagosome–lysosome tethering, resulting in impaired mitophagy and persistent leakage of mitochondrial DNA. This, in turn, drives an exaggerated activation of the cGAS–STING pathway, independent of acute infection. We propose that this chronic, low-level innate immune activation may precondition the immune system, thereby enhancing antiviral responses while simultaneously promoting sterile inflammation. In this context, defective mitophagy and mtDNA release provide a mechanistic link between autophagic failure, heightened innate immune response, and the absent incidence of influenza observed in patients with *EPG5*-related disorders.

Reviewer #4 (Remarks to the Author):

Kritarth Singh, et al. show that multiple mitochondrial functions are changed in EPG5 mutant patient cells, such as mitochondrial membrane potential, mitochondrial morphology, impaired mitochondrial bioenergetics, mitochondrial Ca2+ overload, vulnerability to mPTP opening and activation of mtDNA-cGAS-STING-IFN pathway. The authors propose that these events contribute to EPG5 pathogenesis. However, the conclusions are not well supported by the data presented. Below are some concerns about the manuscript.

We thank Reviewer 4 for their constructive comments and thoughtful suggestions. We are concerned that the reviewer doesn't feel that the data presented support the conclusions and have therefore performed additional experiments and revised the manuscript to address these concerns as thoroughly as possible.

Major

Reviewer:
1. EPG5 is a tethering protein that facilitates fusion between autophagosomes and lysosomes, yet the authors only investigated its role in mitochondrial function. What is the mechanism by which EPG5, a protein primarily acting in another organelle system, regulates mitochondrial function? This is something the authors need to elucidate.

Authors:
Please see our detailed response to **Comment 1 from Reviewer #1**. **We have revised the Introduction (lines: 203-206) and Discussion (lines: 950-960) sections of the manuscript to more clearly address this question.**

In our recent article published in *Annals of Neurology* (Dafsari et al., 2025), we demonstrated how the primary autophagy defect caused by EPG5 deficiency directly impairs mitochondrial quality control pathways. These studies were performed in patient-derived fibroblasts carrying the Q336R and R1621G mutations, as well as in mouse embryonic fibroblasts (MEFs) from Epg5 knock-in mice harbouring the Q331R mutation, which is homologous to the human Q336R variant.

We showed that EPG5 deficiency causes a pronounced autophagosome–lysosome fusion defect (Dafsari et al., 2025: Fig. 6A–D, Supplementary Fig. 7A–B), consistent with the known role of EPG5 as a RAB7 effector ensuring autophagosome-lysosome fusion specificity. This

leads to a stall in mitophagy flux and impaired mitochondrial clearance, thereby permitting the accumulation of dysfunctional mitochondria. Assessment of mitophagy flux by immunoblotting (Dafsari et al., 2025: Fig. 6E–F), together with live-cell imaging of patient fibroblasts expressing the quantitative mitophagy reporter mt-Keima, revealed a marked impairment in PINK1/Parkin-dependent mitophagy (Dafsari et al., 2025: Fig. 6G–M).

In Q331R MEFs, quantitative analysis further demonstrated an accumulation of enlarged, swollen mitophagosomes by transmission electron microscopy (Dafsari et al., 2025: Fig. 4J– L), while flow cytometry showed a severely reduced rate of Parkin-dependent delivery of damaged mitochondria to lysosomes compared with wild-type MEFs (Dafsari et al., 2025: Fig. 4M–N). Consistent with the impaired mitochondrial bioenergetic capacity observed in Q336R and R1621G patient fibroblasts, measurements of mitochondrial oxygen consumption in Q331R MEFs revealed a significant reduction in both ATP-linked respiration and spare respiratory capacity relative to wild-type controls (Dafsari et al., 2025: Fig. 4O–Q).

These data provide direct evidence that EPG5 deficiency leads to mitochondrial dysfunction through a failure of autophagic removal of damaged mitochondria, thereby allowing the progressive accumulation of mitochondrial defects in patient-derived cells.

Reviewer:
2. A homozygous or heterozygous missense mutation affecting a single amino acid (such as Q336R or R1621G) can lead to undetectable protein expression and a reduction in EPG5 mRNA levels. What is the mechanism behind this phenomenon? In cells, if one overexpresses wild-type and mutant EPG5 (Q336R, R1621G), will the mutant EPG5 fail to be expressed?

Authors:
Please see our detailed response to **Comment 4 from Reviewer #1. We have now summarized our response in the Discussion section of the revised manuscript (lines: 974-983)**

Both transcriptional and post-translational mechanisms influence the overall abundance and stability of mutant EPG5 protein in patient-derived fibroblasts carrying missense and truncating variants. A previous report (Kane et al., 2016), along with recent findings from our group (manuscript submitted), confirms that the Q336R missense variant affects pre-mRNA splicing, leading to the generation of multiple aberrant splice isoforms. These aberrant isoforms account for >80% of residual transcripts, most of which harbour a premature

termination codon and are subsequently degraded by nonsense-mediated mRNA decay. Similarly, R1631Q, another homozygous founder missense variant in EPG5, disrupts mRNA splicing and produces an aberrant isoform with an in-frame deletion of 18 amino acids, with complete absence of the wild-type isoform (Vansenne et al., 2022). **Together, these findings indicate that Q336R and R1631Q mutations create null alleles through defective mRNA splicing.**

At the protein level, degradation of wild-type EPG5 is primarily regulated by the autophagy–lysosomal pathway (ALP), as shown above (**please see Figure 3 in response to Comment 4 from Reviewer #1**). In contrast, we did not observe accumulation of mutant EPG5 protein in patient-derived fibroblasts. This is likely due to the extremely low levels of residual *EPG5* transcripts available for translation and/or the rapid turnover of the mutant protein. These findings further suggest that ectopic expression of mutant *EPG5* cDNA, which bypasses defective pre-mRNA splicing and nonsense-mediated mRNA decay, may still result in rapid degradation via the ALP.

Reviewer:

3. The entire study investigates the effects of EPG5 deficiency on mitochondria using patient cells. However, given the small sample size (n=4) and individual variability between cells, and since the paper focuses on EPG5, the authors should generate EPG5-deficient (knockdown or knockout) cells in a healthy cell line, or rescue patient cells with EPG5 to rule out patient genetic background effects. This would allow them to compare the effects on mitochondria in the absence of EPG5 versus after its reintroduction. Specifically, they should examine mitochondrial calcium dynamics and the cGAS-STING-IFN response. This approach would better align with the theme of the paper and highlight the role of EPG5 in mitochondrial regulation.

Authors:

Please see our reply to the comment 3 of the Reviewer #1. The sample number is small as this devastating disease is (fortunately) incredibly rare.

We have addressed the effect of interpatient variability and the impact of wild type *EPG5* expression on mitochondrial function and Ca^{2+} signaling in iPSC-derived cortical neurons, by utilizing an isogenic control iPSC line in which the *EPG5* c.1007A>G (p.Q336R) mutation was corrected using CRISPR/Cas9, thereby restoring wild-type *EPG5* expression **(Fig. 7 and 8 of this manuscript)** in the mutant line and excluding variations between subjects.

Moreover, in order to investigate the rescue of the mitochondrial Ca^{2+} dynamics and the cGAS-STING-IFN response by wild type EPG5 in fibroblasts, we performed siRNA-mediated knockdown of *EPG5* in hTERT-immortalized skin fibroblasts, followed by ectopic expression of EPG5-GFP (immunoblotting) or untagged pCMV6-EPG5 (Airyscan imaging). These cell lines were subsequently analysed for cytosolic mtDNA release and activation of STING-dependent type-I IFN signaling and revealed both the *EPG5* knockdown recapitulated the effects of the pathogenic mutations and that these were reversed by ectopic expression of wild type *EPG5*.

These findings have now been incorporated into the Results section of the revised manuscript (lines: 697-728 and 732-741) and are presented in the revised Figure 5 and 6

Reviewer:
4. For the mitochondrial Ca^{2+} part, mitochondrial Ca^{2+} levels are higher in patient cells, but Calcium Green-5N data shows that mitochondrial calcium buffering is lower, making them more vulnerable to mitochondrial Ca^{2+} overload and mPTP opening. How can this be explained?

Authors:
The method used to demonstrate mitochondrial Ca^{2+} levels in **Fig. 2a and 2d** of this manuscript, measures the rate of mitochondrial Ca^{2+} uptake and the [Ca2+]m in response to the increase in the $[Ca^{2+}]_c$ by histamine in intact fibroblasts, while the Calcium Green-5N data **(Fig. 2k)** measures the maximal retention capacity of the exogenous $[Ca^{2+}]$ boluses and the mPTP opening in the isolated mitochondria from the fibroblasts using an in vitro set up described in the Calcium retention capacity assay of the Method section.

Reviewer:
5. In lines 264-266, the authors mention that they aim to investigate the role of MCU proteins in calcium regulation. However, they did not perform overexpression or further knockdown of MCU proteins to assess this. Instead, they used a CypD inhibitor.

Authors:
The **comments 5 and 6** refer to results described in lines 263–276, indicating that the downregulation of MICU1 and MICU2 sensitises EPG5-deficient patient fibroblasts to $[Ca^{2+}]_m$ overload and mPTP opening. To confirm this, we overexpressed MICU1 in patient fibroblasts as the MICU1 subunit in the MICU1-2 heterodimer gates opening of the MCU pore

(Supplementary Fig. 2e, f). A detailed response is provided below in response to comment 6.

Reviewer:

6. Throughout the entire paragraph from lines 263–276, the authors did not manipulate MICU1 or MICU2 expression, yet they drew conclusions about MICU1 and MICU2. To support these conclusions, the authors need to overexpress MICU proteins in patient cells.

Authors:

To directly address this point, we performed gain-of-function experiments to determine whether restoring MICU1 expression rescues mitochondrial Ca^{2+} handling in EPG5-deficient patient fibroblasts. Ectopic expression of MICU1-HA significantly reduced elevated resting mitochondrial Ca^{2+} levels in patient cells to values comparable with controls, consistent with restoration of MICU1 gatekeeping function **(Supplementary Fig. 2g, h)**. Upon histamine stimulation, MICU1-expressing patient fibroblasts exhibited a significantly reduced peak mitochondrial Ca^{2+} uptake **(Supplementary Fig. 2i, j)**, indicating re-establishment of the MCU Ca^{2+} uptake threshold.

Furthermore, isolated mitochondria from MICU1-expressing patient fibroblasts displayed a markedly increased Ca^{2+} retefntion capacity and tolerated significantly more Ca^{2+} pulses before mPTP opening compared with mitochondria from untreated patient cells **(Supplementary Fig. 2m, n)**.

Together, these data demonstrate that reduced MICU1 expression lowers the mitochondrial Ca^{2+} uptake threshold and increases susceptibility to Ca^{2+} overload and mPTP opening in EPG5-deficient cells, while restoration of MICU1 expression effectively rescues these defects. **These findings have now been incorporated into the Results section of the revised manuscript (lines: 383-406 and 441-447) and are presented in the revised Supplementary Figure 2.**

Reviewer:

7. It is well established that silencing MICU1 abolish mitochondrial calcium entry. How to explain the reduced MICU1, MICU2 accompanied by increased mitochondrial Ca2+ in this study.

Authors:

MICU1 and MICU2 together define the sigmoidal dependence of mitochondrial calcium entry on calcium concentration (inhibition at extramitochondrial [Ca^{2+}] < ~ 500nM, stimulation at higher Ca^{2+} levels), sensing the calcium concentration in the intermembrane space and regulating the opening of the MCU (Csordas et al., 2013; Logan et al., 2014; Patron et al., 2014). There is general consensus, from studies using MICU1 knockout mouse models and from patients who have mutations of MICU1, that genetic ablation of MICU1 leads to mitochondrial calcium overload in resting conditions (Antony et al., 2016; Bhosale et al., 2017; Liu et al., 2016; Patron et al., 2014). Analyses of cellular data obtained under supraphysiological calcium levels or strong agonist stimulation in MICU1-deficient cells have often produced conflicting results. Some studies have argued that knockdown of MICU1 does not alter mitochondrial calcium uptake following agonist-induced calcium release (Mallilankaraman et al., 2012). Other studies have reported that MICU1-deficient cells are impaired in this capacity (Csordas et al., 2013), while still others have demonstrated that the effects may depend on the strength of the agonist stimulation (de la Fuente et al., 2014).

It is likely that differences in experimental approaches including variations in the degree of residual MICU1 expression and intrinsic differences in the composition of the uniporter complex across different cell models may at least partially explain the divergent findings from our study.

The mitochondrial calcium overload that we have described in these cells is entirely consistent with and expected from the literature on MICU1 biology.

Reviewer:

8. In Figure 3, the authors need to block or reduce mitochondrial $Ca^{2}{+}$ to assess cytosolic mtDNA release. This is necessary to demonstrate that the release of cytosolic mtDNA and downstream activation of cGAS-STING are due to mitochondrial calcium overload.

Authors:

We acknowledge this important point. To address this, we performed additional experiments using Ru360, a selective MCU inhibitor, to directly test whether blocking mitochondrial $Ca^{2}{+}$ uptake reduces cytosolic mtDNA release in EPG5-deficient fibroblasts. Ru360 pretreatment markedly attenuated both the rate and amplitude of histamine-induced mitochondrial $Ca^{2}{+}$ uptake in control and patient cells (**(Supplementary Fig. 3e-g)**, confirming effective inhibition of MCU-dependent $Ca^{2}{+}$ entry. Live-cell imaging of Ru360-treated patient fibroblasts revealed a strongly reduced mitochondrial $Ca^{2}{+}$ signal, a mild and sustained decrease in $\Delta\Psi_m$, and,

importantly, a substantial reduction in the frequency of cytosolic mtDNA release following histamine stimulation **(Fig. 3h, Supplementary Fig. 3h, i and Supplementary movie 3)**.

Together, these data demonstrate that mitochondrial Ca^{2+} uptake is required for $[Ca^{2+}]_m$ overload–dependent mtDNA release and support our conclusion that mitochondrial Ca^{2+} overload is a key upstream trigger of cGAS–STING pathway activation in EPG5-deficient fibroblasts. **These findings have now been incorporated into the Results section of the revised manuscript (lines: 518-529) and are presented in the revised Figure 3 and supplementary Figure 3.**

Reviewer:

9. Figure 4H, and S4G-K, the patient cells have increased mitochondrial calcium, so authors should reduce mitochondrial calcium or block mitochondrial calcium, not using TG or histamine to further enhance mitochondrial calcium. The TG or histamine induced mitochondrial calcium overload is artificial. Authors should reduce mitochondrial calcium to detect cGAS-STING pathway in patient cells or EPG5 KO/KD cells.

Authors:

As suggested by the reviewer, we performed siRNA-mediated knockdown of *EPG5* in hTERT-immortalized fibroblasts and pharmacologically inhibited mitochondrial Ca^{2+} uptake using Ru360. EPG5 knockdown induced robust activation of STING-dependent type I IFN signaling, evidenced by increased IRF3 and STAT1 phosphorylation, elevated STING expression, and upregulation of interferon-stimulated genes **(Fig. 4j and Supplementary Fig. 4l-p)**. Importantly, Ru360 treatment significantly attenuated IRF3 and STAT1 activation, reduced STING expression, and lowered ISG mRNA levels in EPG5-deficient cells (**(Fig. 4j and Supplementary Fig. 4l-p)**.

These results demonstrate that excessive mitochondrial Ca^{2+} uptake and overload drive mtDNA-dependent activation of the cGAS–STING pathway and the type I IFN response in EPG5-deficient fibroblasts. **These data have now been incorporated into the Results section of the revised manuscript (lines: 597-606) and are presented in the revised Figure 4 and supplementary Figure 4.**

Reviewer:

10. Again, in Figure 5, the authors only inhibited mPTP but did not reduce mitochondrial Ca^{2+} levels. They need to lower mitochondrial Ca^{2+} to assess the STING-dependent IFN response.

Additionally, when mitochondrial calcium is further increased to induce overload, inhibition of mPTP followed by measurement of the cGAS-STING-IFN response is necessary. This approach is required to clearly demonstrate the effect of mitochondrial calcium on the STING-IFN pathway.

Authors:

Please see our responses to comments 8 and 9 above: we have shown that inhibition of mitochondrial Ca^{2+} uptake by Ru360 treatment effectively blocks mitochondrial Ca^{2+} uptake and consequently attenuates STING-dependent type-I IFN signaling in EPG5-deficient fibroblasts. Given that Ru360 prevents $[Ca^{2+}]_m$ overload and its downstream effects, we believe that further inducing mitochondrial Ca^{2+} overload and mPTP opening in Ru360-pretreated patient fibroblasts would not yield interpretable or meaningful results.

Reviewer:

11. Figures 6-8: The role of mtDNA release from mPTP opening in triggering the cGAS-STING-IFN response is well established. Since JPI-138 is a CypD inhibitor, its effect in improving the cGAS-STING-IFN pathway is predictable. The authors should examine the mitochondrial calcium-mtDNA-cGAS-STING-IFN axis in EPG5 knockout or rescue cells. In addition to patient cells, mPTP inhibitor effects on this pathway should also be tested in EPG5 KO/KD cells.

Authors:

We agree with the reviewer that mPTP opening has previously been implicated in mtDNA release and subsequent activation of the cGAS–STING–IFN pathway (Xian et al., 2022; Yu et al., 2020). However, direct mechanistic evidence defining the sequence of events particularly how mitochondrial Ca^{2+} dysregulation leads to mPTP opening, mtDNA release, and activation of innate immune signaling remains limited, and indeed the mechanisms of mtDNA release remain controversial **(Please see Discussion, lines: 1050-1067)**. Our study provides, for the first time, a detailed temporal analysis linking impaired mitochondrial Ca^{2+} handling to mtDNA release, cGAS–STING pathway activation, and downstream cell death in EPG5-deficient cells.

To further strengthen our conclusions, we examined the impact of restoring EPG5 expression on mitochondrial Ca^{2+} dynamics and the STING-dependent IFN response. Specifically, we performed siRNA-mediated knockdown of *EPG5* in hTERT-immortalized skin fibroblasts followed by ectopic expression of wild-type EPG5-GFP (for immunoblotting) or untagged pCMV6-EPG5 (for Airyscan imaging). These cell lines were subsequently analyzed for mtDNA

release **(Supplementary Fig. 7b-d)** and STING-IFN signaling **(Fig. 5l, m and Supplementary Fig. 6g-k)**. The results clearly demonstrate that re-expression of wild-type *EPG5* suppresses cytosolic mtDNA release and attenuates STING-dependent type-I IFN signaling in EPG5-deficient fibroblasts. **These data have now been incorporated into the Results section of the revised manuscript (lines: 697-728 and 732-741) and are presented in the revised Figure 5 and Supplementary Figure 6.**

Given that we have already established that JP1-138 treatment (a novel CypD inhibitor) effectively reduces cytosolic mtDNA release, dampens STING-dependent IFN responses, and improves mitochondrial bioenergetic function in EPG5-deficient fibroblasts, we believe that further testing mPTP inhibition in EPG5-siRNA cells would not provide additional mechanistic insight or strengthen the conclusions of the study.

Minor

Reviewer:

1. In Abstract, line 43, downregulation of MICU1/2. Authors only detected MICU1 and MICU2 proteins in Figure 2H.

Authors:

We apologise for this ambiguity. Due to word-limit constraints, we abbreviated MICU1 and MICU2 as MICU1/2. We have now revised the abstract to specifically refer to MICU1, as our study primarily focuses on MICU1 downregulation and its functional manipulation in EPG5-deficient patient fibroblasts.

Reviewer:

2. The reporter used to detect NADH is not clearly described.

Authors:

The redox state of the NAD(P)H pool is imaged through measurements of autofluorescence (i.e. label free), as NADH is itself fluorescent, as described in the Methods section. We have revised the relevant text in the **Results section (line: 295)** to clarify the measurement approach.

Reviewer:

3. The molecular weight of ATP5A is approximately 55 kDa, but in the paper it appears below 37 kDa (Fig. 2H, 7F).

Authors:

We thank the reviewer for highlighting this issue and apologize for the oversight. We have now corrected the figures to accurately reflect the ATP5A band based on the unprocessed blot data.

Reviewer:

4. The method for detecting mitochondrial complexes on a single blot in Figure 1K needs to be clearly explained.

Authors:

We have used the OxPhos Human WB Antibody Cocktail (45-8199), which contains a mixture of antibodies targeting each of the mitochondrial respiratory chain complexes. We have now revised the relevant text in the **Results section (lines: 301-312)** to clearly describe this approach.

Reviewer:

5. Figures 2F and 2D are inconsistent. According to the legend in Figure 2F, the “time when mitochondrial $Ca^{2}+$ rise was at half-maximal” is described, but the figure actually shows the time when mitochondrial $Ca^{2}+$ rise was at the maximum.

Authors:

We thank the reviewer for highlighting this inconsistency. We have now corrected the plot in **Fig. 2f** and correctly showed the peak $[Ca^{2+}]_m$ in control 1 mean trace in **Fig. 2d**.

Reviewer:

6. In Figure 4I-J, * labeling should be patient vs ctrl, not patient vs patient

Authors:

We have corrected the labelling to clearly indicate the comparison between patient and control samples.

Reviewer:

7. In Figure 4J, p-STAT1/STAT1 analysis doesn’t match WB results in Figure 4H.

Authors:

We apologise for the confusion. The plot in Fig. 4j represents the p-STAT1/STAT1 ratio normalised to the control 1 ratio, as described in the Fig. 4h legend.

Reviewer:

8. Missing protein molecular weight in Figure 5F and Figure S5A

Authors:

Added.

Reviewer:

9. All WB should be on the same membrane in Figure S5A.

Authors:

Done.

REFERENCES

Acin-Perez, R., Lechuga-Vieco, A.V., Del Mar Munoz, M., Nieto-Arellano, R., Torroja, C., Sanchez-Cabo, F., Jimenez, C., Gonzalez-Guerra, A., Carrascoso, I., Beninca, C., *et al.* (2018). Ablation of the stress protease OMA1 protects against heart failure in mice. Sci Transl Med *10*.

Antony, A.N., Paillard, M., Moffat, C., Juskeviciute, E., Correnti, J., Bolon, B., Rubin, E., Csordas, G., Seifert, E.L., Hoek, J.B., *et al.* (2016). MICU1 regulation of mitochondrial Ca(2+) uptake dictates survival and tissue regeneration. Nat Commun *7*, 10955.

Aoyagi, K., Ohara-Imaizumi, M., Itakura, M., Torii, S., Akimoto, Y., Nishiwaki, C., Nakamichi, Y., Kishimoto, T., Kawakami, H., Harada, A., *et al.* (2016). VAMP7 Regulates Autophagy to Maintain Mitochondrial Homeostasis and to Control Insulin Secretion in Pancreatic beta-Cells. Diabetes *65*, 1648-1659.

Bahat, A., Milenkovic, D., Cors, E., Barnett, M., Niftullayev, S., Katsalifis, A., Schwill, M., Kirschner, P., MacVicar, T., Giavalisco, P., *et al.* (2025). Ribonucleotide incorporation into mitochondrial DNA drives inflammation. Nature *647*, 726-734.

Baron, O., Boudi, A., Dias, C., Schilling, M., Nolle, A., Vizcay-Barrena, G., Rattray, I., Jungbluth, H., Scheper, W., Fleck, R.A., *et al.* (2017). Stall in Canonical Autophagy-Lysosome Pathways Prompts Nucleophagy-Based Nuclear Breakdown in Neurodegeneration. Curr Biol *27*, 3626-3642 e3626.

Bhosale, G., Sharpe, J.A., Koh, A., Kouli, A., Szabadkai, G., and Duchen, M.R. (2017). Pathological consequences of MICU1 mutations on mitochondrial calcium signalling and bioenergetics. Biochim Biophys Acta Mol Cell Res *1864*, 1009-1017.

Borsa, M., Lechuga-Vieco, A.V., Kayvanjoo, A.H., Jenkins, E., Yazicioglu, Y., Compeer, E.B., Richter, F.C., Rapp, S., Mitchell, R., Youdale, T., *et al.* (2025). Autophagy-regulated mitochondrial inheritance controls early CD8(+) T cell fate commitment. Nat Cell Biol.

Chakrabarty, Y., Yang, Z., Chen, H., and Chan, D.C. (2024). The HRI branch of the integrated stress response selectively triggers mitophagy. Mol Cell *84*, 1090-1100 e1096.

Csordas, G., Golenar, T., Seifert, E.L., Kamer, K.J., Sancak, Y., Perocchi, F., Moffat, C., Weaver, D., Perez, S.F., Bogorad, R., *et al.* (2013). MICU1 controls both the threshold and cooperative activation of the mitochondrial Ca(2)(+) uniporter. Cell Metab *17*, 976-987.

Dafsari, H.S., Deneubourg, C., Singh, K., Maroofian, R., Suprenant, Z., Kho, A.L., Ingham, N.J., Steel, K.P., Sheshadri, P., Baur, F., *et al.* (2025). Mutations in the Key Autophagy Tethering Factor EPG5 Link Neurodevelopmental and Neurodegenerative Disorders Including Early-Onset Parkinsonism. Ann Neurol *98*, 932-950.

de la Fuente, S., Matesanz-Isabel, J., Fonteriz, R.I., Montero, M., and Alvarez, J. (2014). Dynamics of mitochondrial Ca2+ uptake in MICU1-knockdown cells. Biochem J *458*, 33-40.
Gu, H., Shi, X., Liu, C., Wang, C., Sui, N., Zhao, Y., Gong, J., Wang, F., Zhang, H., Li, W., *et al.* (2019). USP8 maintains embryonic stem cell stemness via deubiquitination of EPG5. Nat Commun *10*, 1465.

Hooftman, A., Peace, C.G., Ryan, D.G., Day, E.A., Yang, M., McGettrick, A.F., Yin, M., Montano, E.N., Huo, L., Toller-Kawahisa, J.E., *et al.* (2023). Macrophage fumarate hydratase restrains mtRNA-mediated interferon production. Nature *615*, 490-498.

Kane, M.S., Vilboux, T., Wolfe, L.A., Lee, P.R., Wang, Y., Huddleston, K.C., Vockley, J.G., Niederhuber, J.E., and Solomon, B.D. (2016). Aberrant splicing induced by the most common EPG5 mutation in an individual with Vici syndrome. Brain *139*, e52.

Kim, J., Gupta, R., Blanco, L.P., Yang, S., Shteinfer-Kuzmine, A., Wang, K., Zhu, J., Yoon, H.E., Wang, X., Kerkhofs, M., *et al.* (2019). VDAC oligomers form mitochondrial pores to release mtDNA fragments and promote lupus-like disease. Science *366*, 1531-1536.

Kohlschmidt, N., Elbracht, M., Czech, A., Hausler, M., Phan, V., Topf, A., Huang, K.T., Bartok, A., Eggermann, K., Zippel, S., *et al.* (2021). Molecular pathophysiology of human MICU1 deficiency. Neuropathol Appl Neurobiol *47*, 840-855.

Konig, T., Troder, S.E., Bakka, K., Korwitz, A., Richter-Dennerlein, R., Lampe, P.A., Patron, M., Muhlmeister, M., Guerrero-Castillo, S., Brandt, U., *et al.* (2016). The m-AAA Protease Associated with Neurodegeneration Limits MCU Activity in Mitochondria. Mol Cell *64*, 148162.

Lee, S., Kalugotla, G., Ingle, H., Rodgers, R., Wu, C., Wang, Y., Li, Y., Yang, X., Zhang, J., Borella, N.R., *et al.* (2022). Intestinal antiviral signaling is controlled by autophagy gene Epg5 independent of the microbiota. Autophagy *18*, 1062-1077.

Liu, J.C., Liu, J., Holmstrom, K.M., Menazza, S., Parks, R.J., Fergusson, M.M., Yu, Z.X., Springer, D.A., Halsey, C., Liu, C., *et al.* (2016). MICU1 Serves as a Molecular Gatekeeper to Prevent In Vivo Mitochondrial Calcium Overload. Cell Rep *16*, 1561-1573.

Logan, C.V., Szabadkai, G., Sharpe, J.A., Parry, D.A., Torelli, S., Childs, A.M., Kriek, M., Phadke, R., Johnson, C.A., Roberts, N.Y., *et al.* (2014). Loss-of-function mutations in MICU1 cause a brain and muscle disorder linked to primary alterations in mitochondrial calcium signaling. Nat Genet *46*, 188-193.

Lu, Q., Yokoyama, C.C., Williams, J.W., Baldridge, M.T., Jin, X., DesRochers, B., Bricker, T., Wilen, C.B., Bagaitkar, J., Loginicheva, E., *et al.* (2016). Homeostatic Control of Innate Lung Inflammation by Vici Syndrome Gene Epg5 and Additional Autophagy Genes Promotes Influenza Pathogenesis. Cell Host Microbe *19*, 102-113.

Mallilankaraman, K., Doonan, P., Cardenas, C., Chandramoorthy, H.C., Muller, M., Miller, R., Hoffman, N.E., Gandhirajan, R.K., Molgo, J., Birnbaum, M.J., *et al.* (2012). MICU1 is an essential gatekeeper for MCU-mediated mitochondrial Ca(2+) uptake that regulates cell survival. Cell *151*, 630-644.

Nakahira, K., Haspel, J.A., Rathinam, V.A., Lee, S.J., Dolinay, T., Lam, H.C., Englert, J.A., Rabinovitch, M., Cernadas, M., Kim, H.P., *et al.* (2011). Autophagy proteins regulate innate immune responses by inhibiting the release of mitochondrial DNA mediated by the NALP3 inflammasome. Nat Immunol *12*, 222-230.

Patron, M., Checchetto, V., Raffaello, A., Teardo, E., Vecellio Reane, D., Mantoan, M., Granatiero, V., Szabo, I., De Stefani, D., and Rizzuto, R. (2014). MICU1 and MICU2 finely tune the mitochondrial Ca2+ uniporter by exerting opposite effects on MCU activity. Mol Cell *53*, 726-737.

Piano Mortari, E., Folgiero, V., Marcellini, V., Romania, P., Bellacchio, E., D'Alicandro, V., Bocci, C., Carrozzo, R., Martinelli, D., Petrini, S., *et al.* (2018). The Vici syndrome protein EPG5 regulates intracellular nucleic acid trafficking linking autophagy to innate and adaptive immunity. Autophagy *14*, 22-37.

Plotegher, N., Filadi, R., Pizzo, P., and Duchen, M.R. (2021). Excitotoxicity Revisited: Mitochondria on the Verge of a Nervous Breakdown. Trends Neurosci *44*, 342-351.

Singh, R., Bartok, A., Paillard, M., Tyburski, A., Elliott, M., and Hajnoczky, G. (2022). Uncontrolled mitochondrial calcium uptake underlies the pathogenesis of neurodegeneration in MICU1-deficient mice and patients. Sci Adv *8*, eabj4716.

Thornton, B.T., Hardinger, A.G., Pence, L., Prem Kumar, P., Connolly, N., Weir, S.J., and Vivian, J.L. (2025). Progressive neuroinflammation and deficits in motor function in a mouse model with an Epg5 pathogenic variant of Vici syndrome. bioRxiv.

Tsai, C.W., Rodriguez, M.X., Van Keuren, A.M., Phillips, C.B., Shushunov, H.M., Lee, J.E., Garcia, A.M., Ambardekar, A.V., Cleveland, J.C., Jr., Reisz, J.A., *et al.* (2022). Mechanisms and significance of tissue-specific MICU regulation of the mitochondrial calcium uniporter complex. Mol Cell *82*, 3661-3676 e3668.

Uoselis, L., Lindblom, R., Lam, W.K., Kung, C.J., Skulsuppaisarn, M., Khuu, G., Nguyen, T.N., Rudler, D.L., Filipovska, A., Schittenhelm, R.B., *et al.* (2023). Temporal landscape of mitochondrial proteostasis governed by the UPR(mt). Sci Adv *9*, eadh8228.

Vansenne, F., Fock, J.M., Stolte-Dijkstra, I., Meiners, L.C., van den Boogaard, M.H., Jaeger, B., Boven, L., Vos, Y.J., Sinke, R.J., and Verbeek, D.S. (2022). Phenotypic expansion of EGP5-related Vici syndrome: 15 Dutch patients carrying a founder variant. Eur J Paediatr Neurol *41*, 91-98.

Victorelli, S., Salmonowicz, H., Chapman, J., Martini, H., Vizioli, M.G., Riley, J.S., Cloix, C., Hall-Younger, E., Machado Espindola-Netto, J., Jurk, D., *et al.* (2023). Apoptotic stress causes mtDNA release during senescence and drives the SASP. Nature *622*, 627-636.

West, A.P., Khoury-Hanold, W., Staron, M., Tal, M.C., Pineda, C.M., Lang, S.M., Bestwick, M., Duguay, B.A., Raimundo, N., MacDuff, D.A., *et al.* (2015). Mitochondrial DNA stress primes the antiviral innate immune response. Nature *520*, 553-557.

Whitehead, M., Harvey, J.P., Sladen, P.E., Becchi, G., Singh, K., Sun, Y.J., Burgoyne, T., Duchen, M.R., Yu-Wai-Man, P., and Cheetham, M.E. (2025). Disruption of mitochondrial homeostasis and permeability transition pore opening in OPA1 iPSC-derived retinal ganglion cells. Acta Neuropathol Commun *13*, 28.

Xian, H., Watari, K., Sanchez-Lopez, E., Offenberger, J., Onyuru, J., Sampath, H., Ying, W., Hoffman, H.M., Shadel, G.S., and Karin, M. (2022). Oxidized DNA fragments exit mitochondria via mPTP- and VDAC-dependent channels to activate NLRP3 inflammasome and interferon signaling. Immunity *55*, 1370-1385 e1378.

Yamada, T., Ikeda, A., Murata, D., Wang, H., Zhang, C., Khare, P., Adachi, Y., Ito, F., Quiros, P.M., Blackshaw, S., *et al.* (2025). Dual regulation of mitochondrial fusion by Parkin-PINK1 and OMA1. Nature *639*, 776-783.

Yu, C.H., Davidson, S., Harapas, C.R., Hilton, J.B., Mlodzianoski, M.J., Laohamonthonkul, P., Louis, C., Low, R.R.J., Moecking, J., De Nardo, D., *et al.* (2020). TDP-43 Triggers Mitochondrial DNA Release via mPTP to Activate cGAS/STING in ALS. Cell *183*, 636-649 e618.

Yuan, C., Dong, H., Wu, C., Liu, J., Wang, Z., Wang, X., Ren, H., Wang, Z., and Lu, Q. (2025). EPG-5 regulates TGFB/TGF-beta and WNT signalling by modulating retrograde endocytic trafficking. Autophagy *21*, 1995-2008.

Zhong, Z., Umemura, A., Sanchez-Lopez, E., Liang, S., Shalapour, S., Wong, J., He, F., Boassa, D., Perkins, G., Ali, S.R., *et al.* (2016). NF-kappaB Restricts Inflammasome Activation via Elimination of Damaged Mitochondria. Cell *164*, 896-910.
